# Histone Modifications at the Intersection of Aging, Neuroinflammation and Metabolism in Alzheimer's Disease

**DOI:** 10.1111/acel.70785

**Published:** 2026-10-26

**Authors:** Junyi Liang, Huawen Cao, Xiumin Ren, Nan Sha, Xueqing Han, Ling Jiang, Bin Liu, Weiming Zhao

**Affiliations:** ^1^ Heilongjiang University of Chinese Medicine Harbin Heilongjiang Province China; ^2^ The First Affiliated Hospital of Jiamusi University Jiamusi Heilongjiang Province China

**Keywords:** Alzheimer's disease, astrocytes, cellular senescence, chromatin remodeling, histone post‐translational modifications, microglia, neuroinflammation

## Abstract

Aging, neuroinflammation and metabolic dysfunction are tightly intertwined in Alzheimer's disease (AD), but how transient pathological signals become persistent cellular states remains poorly understood. Histone post‐translational modifications (PTMs) provide a dynamic interface between extracellular stimuli, cellular metabolism and chromatin state. This Review examines how canonical histone modifications and emerging metabolite‐derived acylations regulate inflammatory and stress‐responsive programmes across microglia, astrocytes and neurons during aging and AD. We discuss how amyloid‐β, tau pathology, metabolic dysfunction and innate immune signaling converge on histone‐modifying machinery, and how reciprocal interactions between metabolism and chromatin may stabilize disease‐associated cell states. Particular attention is given to cellular senescence and neuroimmune memory, while emphasizing the context dependence of individual histone marks across cell types, genomic regions and disease stages. We further evaluate evidence for causality using three criteria: perturbation, persistence and reversibility. This framework distinguishes chromatin states that accompany ongoing pathology from those capable of sustaining pathological cell states beyond their initiating signals. Finally, we consider therapeutic strategies targeting histone‐modifying enzymes, chromatin readers and metabolic inputs to restore chromatin plasticity and promote resolution of chronic neuroinflammation.

## Graphical abstract

This review examines how histone modifications integrate aging, metabolic dysfunction and neuroinflammation to shape persistent, cell‐type‐specific chromatin states in Alzheimer's disease. By distinguishing causal regulation from association through perturbation, persistence and reversibility, it highlights metabolic–epigenetic feedback loops and therapeutic opportunities for restoring chromatin plasticity.

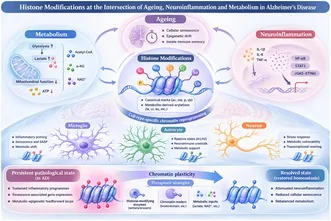

## 1 Introduction

Aging is the strongest risk factor for Alzheimer's disease (AD), in which neurodegeneration emerges from the convergence of proteotoxic stress, immune dysregulation, and metabolic dysfunction (Butovsky et al. 2026; Heneka et al. 2025). Neuroinflammation is now recognized as an active component of AD pathogenesis rather than simply a secondary response to amyloid‐β (Aβ) and tau pathology. During brain aging and AD, microglia, astrocytes, and neurons undergo persistent changes in inflammatory, stress‐responsive, and metabolic programmes, and senescence‐associated states have been implicated in the stabilization of these phenotypes (Hu et al. 2025; Wendeln et al. 2018). Yet continued exposure to pathological stimuli alone does not fully explain why inflammatory and stress‐response programmes can become persistent and maladaptive. A fundamental question is therefore how transient or recurrent pathological signals are converted into durable cellular states.

Chromatin regulation provides a potential mechanism for this persistence. Human and experimental studies have identified extensive, cell‐type‐specific epigenomic remodeling in AD, including alterations in chromatin accessibility, enhancer activity and transcriptional regulation (Ardura‐Fabregat et al. 2025; Green et al. 2024; Liu, Zhang, et al. 2025; Murphy, Hu, et al. 2025; Nativio et al. 2020). Recent single‐cell, multiregion profiling of postmortem AD brains has further revealed widespread changes in regulatory chromatin landscapes across neural and glial populations, linking epigenomic instability to cellular state transitions and disease‐associated phenotypes (Liu, Zhang, et al. 2025). Histone post‐translational modifications (PTMs) are central components of this regulatory system. Acetylation, methylation, phosphorylation and ubiquitination shape nucleosome organization, enhancer and promoter activity, and transcription‐factor recruitment, whereas emerging metabolite‐derived modifications, including lactylation, crotonylation and succinylation, provide additional routes through which cellular physiology can influence chromatin state (Chatila et al. 2024; Datta et al. 2018). Rather than functioning as isolated molecular marks, these modifications act within combinatorial and context‐dependent chromatin environments that can influence the magnitude, duration and reversibility of inflammatory and stress‐responsive transcription. The concept of innate immune memory provides a broader precedent for this principle, showing that prior inflammatory experience can establish durable transcriptional and chromatin states that alter subsequent cellular responses (Wendeln et al. 2018; Yücel and Gladyshev 2026).

Histone modifications are also intimately coupled to cellular metabolism. Many histone‐modifying enzymes require metabolic substrates or cofactors, including acetyl‐CoA, S‐adenosylmethionine, NAD+ and α‐ketoglutarate, thereby linking nutrient availability, mitochondrial function and redox state to chromatin regulation (Yücel and Gladyshev 2026). Conversely, histone modifications can reinforce metabolic and inflammatory programmes, creating reciprocal feedback between cellular metabolism and gene regulation. This coupling is particularly relevant to aging and AD, in which altered glucose utilization, mitochondrial dysfunction and redox imbalance reshape the metabolic environment in which chromatin states are established and maintained. Metabolite‐derived acylations provide illustrative examples of this interface, linking changes in cellular metabolites to transcriptional regulation. Histone lactylation, for example, has been associated with metabolic and inflammatory reprogramming in AD‐related microglia, including glycolysis‐associated H4K12 lactylation and senescence‐associated H3K18 lactylation (Cirovic et al. 2020; Noh et al. 2026; Novakovic et al. 2016). However, these observations should not be interpreted as evidence that any single histone modification is intrinsically pathogenic or protective. The consequences of a given PTM depend on its genomic location, interacting chromatin machinery, cellular context, metabolic state and disease stage.

These considerations raise a broader mechanistic question: which histone modifications actively determine pathological cell states rather than simply reflecting them? Equally important, to what extent do AD‐associated chromatin states persist independently of the initiating stimulus, and can they be experimentally reversed? These questions are difficult to resolve because most studies identify altered chromatin marks under conditions of ongoing pathology, providing evidence of association but not necessarily of persistence. We therefore distinguish three experimentally separable properties of a candidate pathological chromatin state: perturbation, whether altering the modification changes cellular or disease‐relevant phenotypes; persistence, whether the state is maintained beyond the initiating signal; and reversibility, whether an established state can be reset by targeted intervention. This framework helps distinguish transient chromatin responses from mechanisms capable of stabilizing chronic neuroinflammation.

Here, we examine histone PTMs as a dynamic chromatin interface connecting aging, neuroinflammation and metabolism in AD (Figure 1) (Nativio et al. 2020; Qin et al. 2024; Zheng et al. 2019). We first consider how canonical histone modifications and emerging metabolite‐derived modifications shape chromatin states associated with cellular senescence and inflammatory activation. We then examine how these mechanisms operate across microglia, astrocytes and neurons and how Aβ, tau, innate immune signaling and metabolic dysfunction converge on histone‐modifying pathways. Finally, we assess the evidence for causality, persistence and reversibility and consider therapeutic strategies targeting histone‐modifying enzymes, chromatin readers and the metabolic inputs that sustain pathological chromatin states. Rather than assigning fixed functions to individual histone marks, we emphasize the context dependence and combinatorial nature of histone modification and propose that restoring appropriate chromatin plasticity may represent a route toward resolving persistent neuroinflammatory states in AD.

**FIGURE 1:**
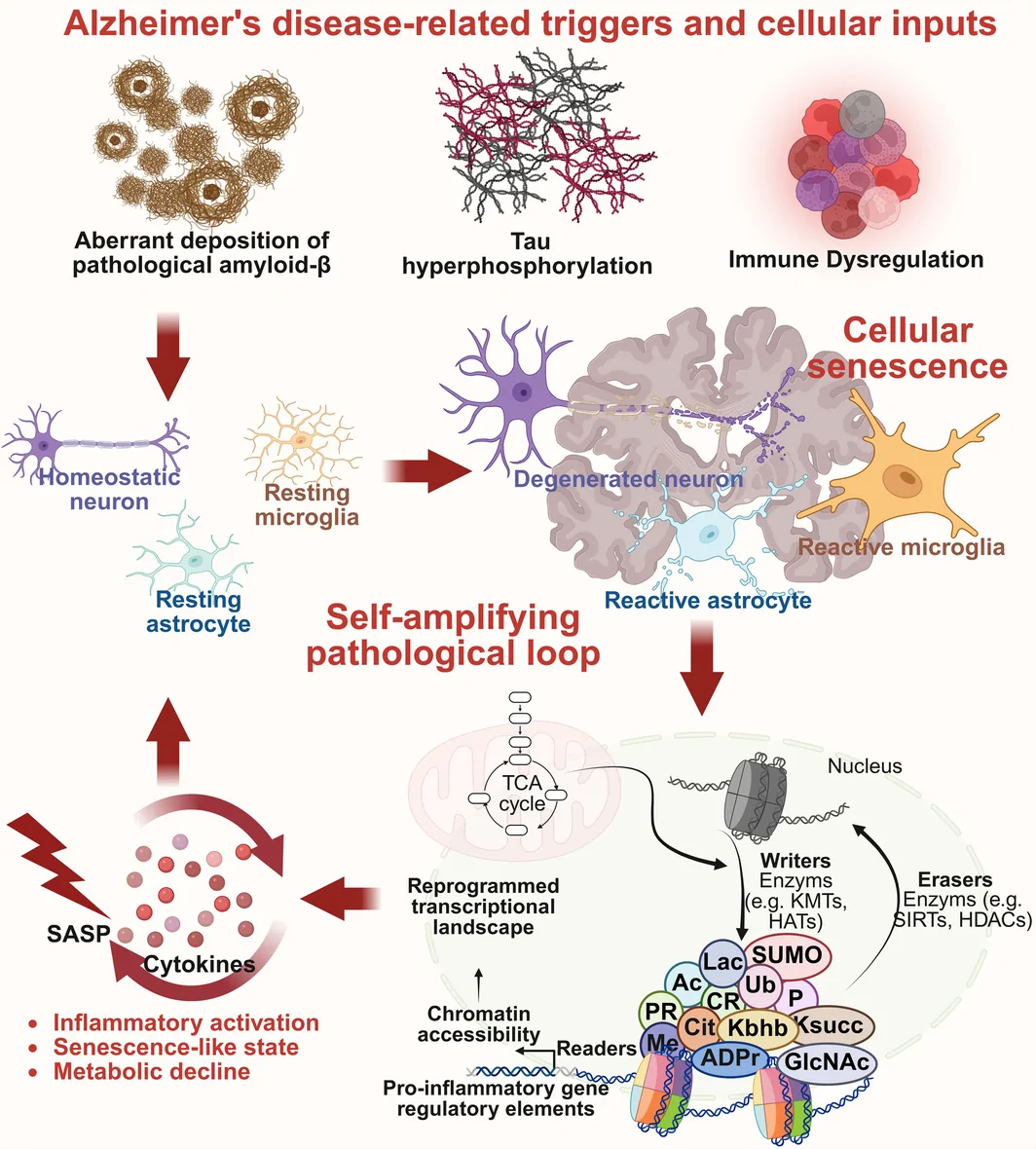
Self‐amplifying epigenetic reprogramming in aging, neuroinflammation, and AD. This schematic depicts how AD‐related triggers and cellular inputs—notably aberrant deposition of pathological amyloid‐β (Aβ), Tau hyperphosphorylation, and immune dysregulation—drive a self‐amplifying pathological loop. These triggers transition homeostatic neurons, resting microglia, and resting astrocytes into degenerated, reactive, and senescent states, driving SASP, cytokine release, inflammatory activation, and metabolic decline that feedback to exacerbate the upstream triggers. In the nucleus, metabolic reprogramming (e.g., the TCA cycle) fosters a reprogrammed transcriptional landscape, where writer enzymes (e.g., KMTs, HATs) and eraser enzymes (e.g., SIRTs, HDACs) dynamically regulate combinatorial histone modifications. Recognized by readers, these marks alter chromatin accessibility at pro‐inflammatory gene regulatory elements, perpetuating the neuroinflammatory cycle. Red arrows denote promoting or feedback effects; circular arrows indicate the self‐amplifying nature of the loop. This figure created in BioRender.com.

## 2 Histone PTMs as a Dynamic Chromatin System

Histone post‐translational modifications (PTMs) are one layer of chromatin regulation, operating alongside DNA methylation, histone variants, nucleosome remodeling, transcription‐factor binding and three‐dimensional genome organization (Duan et al. 2024; Wang, Liang, et al. 2026; Yao et al. 2024). They can influence histone–DNA interactions, nucleosome dynamics and the recruitment or exclusion of chromatin‐associated proteins. Histone PTMs should therefore not be treated as universal switches for transcriptional activation or repression. Their effects depend on the modified residue, modification state, abundance, genomic location, timing and cellular context (Huang et al. 2024; Lin et al. 2025; Lu et al. 2026; Park et al. 2024; Policarpi et al. 2024; Wu, Qu, and Liu 2024; Yamagishi et al. 2024; Zhou and Cole 2025). This framework provides the basis for examining how particular PTMs change in aging and disease in the following chapters.

### 2.1 The Landscape of Histone PTMs

The nucleosome, composed of an octamer containing two copies each of H2A, H2B, H3, and H4 wrapped by approximately 147 base pairs of DNA, is the fundamental structural unit of chromatin. Linker histone H1 contributes to nucleosome spacing and higher‐order chromatin organization. Histone tails and residues within the histone core can undergo a diverse range of enzymatically regulated PTMs, including acetylation, methylation, phosphorylation, ubiquitination, SUMOylation, O‐GlcNAcylation, citrullination, and ADP‐ribosylation, together with an expanding group of metabolite‐derived lysine acylations such as lactylation, crotonylation, and succinylation (Duan et al. 2024; Li, Qiu, et al. 2025; Wang, Liang, et al. 2026; Yao et al. 2024). These modifications can alter nucleosome properties or provide binding sites for regulatory proteins, influencing chromatin accessibility, transcription, DNA repair, and genome stability (Figure 2; Table 1).

**FIGURE 2:**
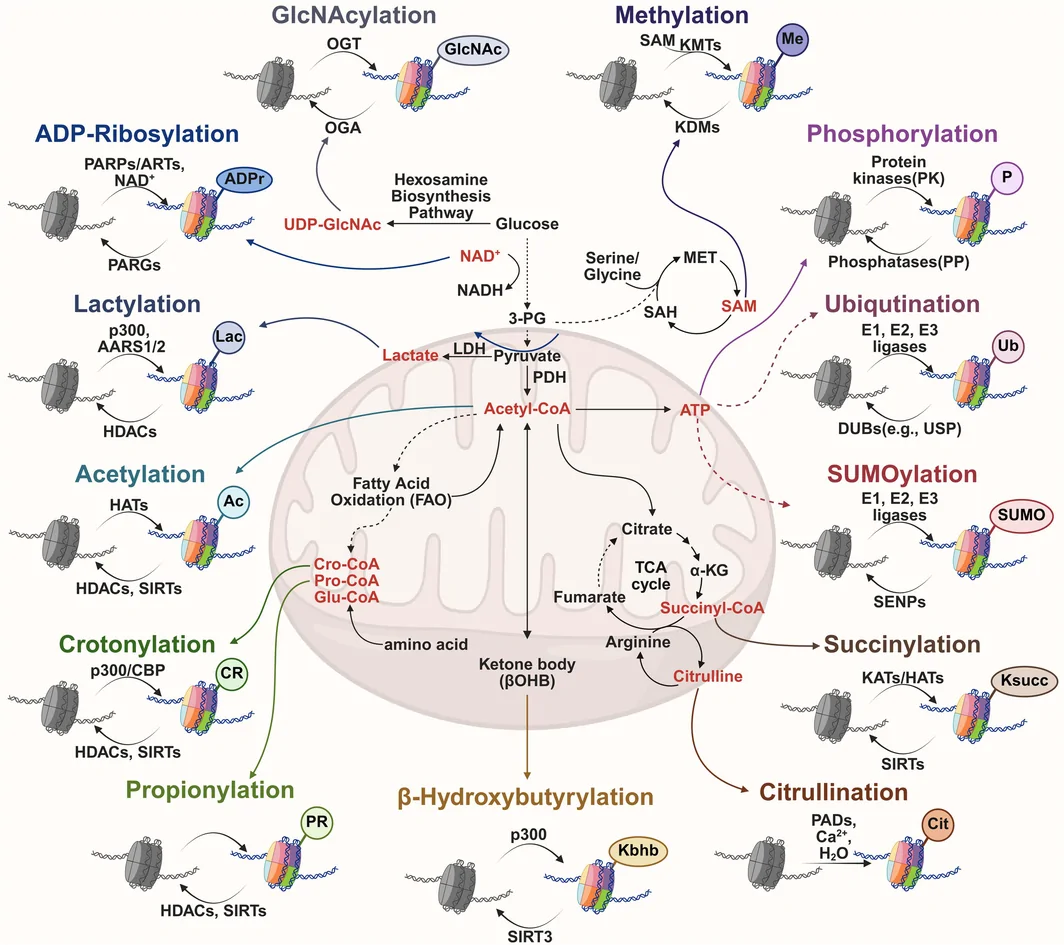
Metabolic regulation of histone post‐translational modifications. This schematic illustrates how central carbon and amino acid metabolic pathways converge to generate key metabolites, which serve as essential substrates and cofactors for diverse histone post‐translational modifications (PTMs), linking metabolic reprogramming to epigenetic dysregulation in neuroinflammation. Glucose, fatty acids, and amino acids act as primary metabolic resources, producing a wealth of intracellular intermediates. Notably, metabolites such as crotonyl‐CoA (Cro‐CoA) and propionyl‐CoA (Pro‐CoA) predominantly arise from fatty acid oxidation and amino acid metabolism, whereas lactate, β‐hydroxybutyrate (βOHB), and succinyl‐CoA primarily originate from glucose metabolism and the TCA cycle, highlighting a diverse metabolic sourcing for lysine acylation. Acetyl‐CoA, SAM, NAD^+^, and UDP‐GlcNAc serve as central hubs connecting these pathways to chromatin. The periphery depicts 13 distinct histone PTMs—including acetylation, lactylation, β‐hydroxybutyrylation, succinylation, crotonylation, propionylation, GlcNAcylation, methylation, phosphorylation, ubiquitination, SUMOylation, citrullination, and ADP‐ribosylation—along with their respective ‘writer’ (e.g., HATs, KMTs, p300/CBP, OGT) and ‘eraser’ (e.g., HDACs, SIRTs, KDMs, OGA) enzymes. In the aging and AD brain, metabolic dysregulation (e.g., glucose hypometabolism, declining NAD^+^ levels, mitochondrial dysfunction) profoundly alters the availability of these key metabolites, driving aberrant histone modification landscapes that transcriptionally activate neuroinflammatory programs. This figure created in BioRender.com.

**TABLE 1:** Representative histone PTM sites and their regulatory machinery.

Modification	Representative histone sites	Writers	Erasers	Readers	Representative chromatin functions	References
Acetylation (Kac)	H3K9ac, H3K14ac, H3K27ac, H4K16ac	p300/CBP; KAT2A/KAT2B (GCN5/PCAF); KAT8/MOF	HDAC1–3; SIRT1, SIRT2, SIRT6	Bromodomain‐containing proteins, particularly BET proteins (BRD2/3/4)	Generally promotes transcriptionally permissive chromatin; enhancer and promoter activation; increased chromatin accessibility	Miziak et al. (2024), Wu, Qu, and Liu (2024), Zhou and Cole (2025)
Methylation (Kme1/2/3)	H3K4me1/2/3, H3K9me2/3, H3K27me1/2/3, H3K36me1/2/3, H3K79me1/2/3, H4K20me1/2/3	SETD1A/B–COMPASS; EZH2–PRC2; SUV39H1/2, SETDB1; SETD2	LSD1/KDM1A; KDM4; KDM5; KDM6 families	PHD fingers; chromodomains/HP1; Tudor domains; PWWP domains	Context‐dependent activation or repression; promoter and enhancer specification; Polycomb repression; heterochromatin formation; transcriptional elongation and chromatin‐state memory	Huang et al. (2024), Park et al. (2024), Policarpi et al. (2024), Wu, Qu, and Liu (2024), Yamagishi et al. (2024)
Phosphorylation (ph)	H3S10ph, H3T3ph, H3S28ph, H2AXS139ph (γH2AX)	Aurora kinases; Haspin; MSK1/2; ATM/ATR and other signaling kinases	PP1, PP2A, PPM1D and other protein phosphatases	14–3‐3 proteins; BRCT‐domain‐containing and other phospho‐dependent proteins	Signal‐responsive chromatin regulation; mitotic chromosome condensation; transcriptional control; DNA‐damage signaling and repair	Lin et al. (2025), Lu et al. (2026), Wang, Liang, et al. (2026), Wu, Qu, and Liu (2024)
Ubiquitination (Ub)	H2AK119ub1, H2BK120ub1	RING1A/RING1B–PRC1; RNF20/RNF40–BRE1 and related E3 ligases	BAP1/PR‐DUB, USP16 and other DUBs for H2AK119ub1; USP22 and related DUBs for H2BK120ub1	RYBP/YAF2 and other ubiquitin/nucleosome‐recognition modules	Polycomb‐associated repression; transcription elongation; crosstalk with other histone modifications; DNA‐damage and genome‐maintenance functions	Ai et al. (2024), Ciapponi et al. (2024), Ge et al. (2023), Onishi et al. (2024), Oss‐Ronen et al. (2022)
SUMOylation	H4K12‐SUMO and additional core‐histone SUMO sites	SAE1/SAE2; Ubc9; PIAS and other SUMO E3 ligases	SENP family proteases	SUMO‐interacting motif (SIM)‐containing proteins; histone‐specific readers remain incompletely defined	Often associated with transcriptional repression and negative crosstalk with active histone marks; can also alter nucleosome–nucleosome interactions and DNA‐repair processes	Leonen et al. (2021), Ryu and Hochstrasser (2021)
O‐GlcNAcylation	H2BS112‐GlcNAc and additional O‐GlcNAc sites on core histones	OGT	OGA	Histone‐specific readers are not yet firmly established; O‐GlcNAc‐dependent chromatin complexes are emerging	Couples glucose–hexosamine metabolism to chromatin regulation; promotes H2BK120 ubiquitination; modulates transcription, chromatin organization and senescence‐associated programmes	Deng et al. (2026), Wu, Li, et al. (2024), Zhang et al. (2026)
ADP‐ribosylation (ADPr)	H2BS6ADPr, H3S10ADPr and additional Ser‐linked histone sites	PARP1/2–HPF1	PARG; ARH3 and other ADP‐ribose hydrolases	ALC1/CHD1L macrodomain; RNF114 and other ADPr/PAR‐binding proteins	Rapid and reversible chromatin relaxation; nucleosome mobilization; DNA‐damage signaling and repair; transient regulation of chromatin accessibility	Anagho et al. (2024), García Fernández et al. (2025), Longarini et al. (2023), Özdemir et al. (2024)
Citrullination	H3R2cit, H3R8cit, H3R17cit, H4R3cit	PADI4 and other PAD family enzymes	No established dedicated histone decitrullinase	No firmly established histone‐specific reader	Reduces the positive charge of arginine residues; promotes chromatin decondensation and transcriptional regulation; contributes to inflammatory chromatin responses	Love et al. (2025), Yao et al. (2024)
Lactylation (Kla)	H3K18la, H3K9la, H3K14la, H4K12la	p300/CBP; HBO1/KAT7; KAT2A	HDAC1–3; SIRT1, SIRT2 and SIRT3 have been reported as delactylases in site‐ and context‐dependent settings	BRG1/SMARCA4 for H3K18la; DPF2 for H3K14la; additional readers are emerging	Couples lactate metabolism to transcriptional regulation; modulates promoter/enhancer activity, metabolic adaptation, inflammatory responses and cell‐state transitions	Moreno‐Yruela et al. (2022), Niu et al. (2024), Sheng et al. (2026), Zhai et al. (2024), Zhang, Zhou, Zhang, et al. (2024), Zhu et al. (2025)
Crotonylation (Kcr)	H3K9cr, H3K18cr, H3K27cr, H4K8cr, H4K12cr	p300/CBP; KAT2A; selected MYST‐family KATs	HDAC1–3; SIRT1–3	YEATS‐domain proteins, including ENL/AF9 and YEATS2; DPF family proteins; recognition is site‐ and context‐dependent	Regulation of promoters and enhancers; frequently associated with transcriptional activation; links crotonyl‐CoA metabolism to chromatin state and inflammatory or stress‐responsive transcription	Gao et al. (2024), Li, Xiang, et al. (2024), Xie et al. (2024)
Succinylation (Ksucc)	H3K122succ and additional histone lysine sites	p300/CBP; other KAT activities have been implicated; site‐specific enzymology remains incompletely defined	SIRT5; additional sirtuin activities remain under investigation	Histone‐specific readers remain poorly defined	Reverses lysine charge; can destabilize nucleosome–DNA interactions and increase chromatin accessibility; links chromatin regulation to succinyl‐CoA/TCA metabolism	Weyh et al. (2024), Zorro Shahidian et al. (2021)

The establishment, removal and recognition of histone PTMs are commonly described in terms of three functional classes of proteins: writers, which install modifications; erasers, which remove them; and readers, which recognize modified residues and help translate them into downstream chromatin functions (Narita et al. 2019). These categories are not strictly discrete. Many chromatin‐modifying enzymes also act on non‐histone substrates, and their activity, localization and substrate engagement can be regulated by signaling pathways, protein stability and cellular state (Park et al. 2024; Wu, Qu, and Liu 2024; Yamagishi et al. 2024; Zhou and Cole 2025). Histone PTMs are therefore embedded within signaling networks rather than operating as autonomous molecular switches.

Different classes of PTMs can have distinct but overlapping effects. Acetylation is often associated with transcriptionally permissive chromatin, in part through changes in nucleosome interactions and the recruitment of acetyl‐lysine readers. Methylation can be associated with either active or repressed chromatin, depending on the modified residue and the degree of methylation (Miziak et al. 2024; Moraitis and Piperi 2025). Phosphorylation, ubiquitination, and SUMOylation further connect chromatin to signal‐responsive transcription, DNA damage responses, and genome maintenance (Ai et al. 2024; Ciapponi et al. 2024; Ge et al. 2023; Leonen et al. 2021; Mattiroli and Penengo 2021; Onishi et al. 2024; Oss‐Ronen et al. 2022; Ryu and Hochstrasser 2021). O‐GlcNAcylation and ADP‐ribosylation provide additional links between nutrient sensing, cellular stress, and chromatin regulation, whereas citrullination can modify histone charge and alter chromatin structure and transcriptional regulation (Anagho et al. 2024; Deng et al. 2026; García Fernández et al. 2025; Hananya et al. 2021; Longarini et al. 2023; Love et al. 2025; Özdemir et al. 2024; Wu, Li, et al. 2024; Zhang et al. 2026). Collectively, these modifications expand the regulatory repertoire of the nucleosome, but their biological outputs cannot be inferred from modification abundance alone.

Metabolite‐derived acylations represent a particularly important extension of this regulatory repertoire because they provide a biochemical route through which cellular metabolism can influence chromatin. Their formation reflects both the availability of metabolic intermediates and the activities of enzymes that install, remove or interpret the corresponding acyl groups (Han et al. 2025; Moreno‐Yruela et al. 2022; Yao et al. 2024). Mechanistic studies of histone lactylation have progressively expanded the underlying regulatory framework, including lactyltransferase activities, delactylases and candidate readers, while also clarifying that enzymatic and alternative lactylation pathways may coexist (Moreno‐Yruela et al. 2022; Niu et al. 2024; Sheng et al. 2026; Zhai et al. 2024; Zhang, Zhou, Zhang, et al. 2024). Histone lactylation is therefore best considered an emerging component of metabolic–chromatin coupling rather than an isolated lactate‐responsive switch. Similar principles apply to crotonylation and succinylation, which connect chromatin regulation to intermediary metabolic states and the availability of crotonyl‐CoA or succinyl‐CoA (Gao et al. 2024; Han et al. 2025; Li, Wang, et al. 2025; Weyh et al. 2024; Xie et al. 2024; Zhu et al. 2025; Zorro Shahidian et al. 2021). Importantly, metabolite abundance alone does not determine the genomic distribution or transcriptional consequence of a histone acylation; enzymatic targeting, chromatin context and competing modifications are also required to specify its functional output.

### 2.2 From Individual Marks to Chromatin States

The function of a histone PTM cannot be inferred from its presence at a single residue alone. Multiple modifications may coexist within the same nucleosome or regulatory region and influence one another through cis and trans crosstalk (Jiang et al. 2026; Kawaf et al. 2024; Li, Ren, et al. 2022). Such interactions can affect the installation, removal, or recognition of neighboring modifications, producing combinatorial chromatin states. Proteomic and mechanistic studies increasingly support this view, showing that histone modifications often occur in combinations rather than as independent events (Jiang et al. 2026; Kawaf et al. 2024; Li, Ren, et al. 2022).

One form of crosstalk occurs when different modifications compete for the same residue. H3K27 acetylation and H3K27 methylation, for example, are mutually exclusive at that lysine. H3K27ac is commonly associated with active regulatory elements, whereas H3K27me3 is associated with Polycomb‐mediated repression (Pasini et al. 2010; Tie et al. 2009). Crosstalk can also occur between adjacent residues. H3S28 phosphorylation, for instance, can influence the status and interpretation of neighboring H3K27 modifications, including in contexts where Polycomb‐associated repression is altered (Chen, Yue, et al. 2026; Li, Ding, et al. 2026; Sun et al. 2024). These examples illustrate how the effect of a modification may depend on the modification state of neighboring residues, rather than on a single mark considered in isolation.

The consequences of PTM crosstalk are also shaped by the regulatory proteins present at a genomic locus. At promoters and enhancers, combinations of histone modifications act alongside transcription factors and chromatin remodelers to influence transcriptional potential (Miziak et al. 2024; Moraitis and Piperi 2025). The same PTM may therefore be associated with different outcomes depending on its location, the surrounding chromatin state, and the proteins available to interpret it.

Cell identity and cell state add further layers of context. Distinct transcription‐factor repertoires, enhancer landscapes, and patterns of chromatin accessibility influence both where modifying enzymes act and how a given modification is interpreted (Hernandez et al. 2023; Li, Ren, et al. 2022). Thus, a histone PTM need not have the same consequence in every cell type or at every stage of a biological process.

Histone PTMs are best viewed as components of dynamic chromatin states rather than as isolated marks. Their effects emerge from the interplay between modification type, genomic location, temporal dynamics, regulatory machinery, and cellular context. This perspective shifts the question from whether a mark is simply increased or decreased to how combinations of PTMs contribute to the establishment, maintenance, or resolution of a cellular state. The next chapter considers how these principles apply to histone modifications in aging.

## 3 Histone and Chromatin Remodeling During Cellular Senescence: A Mechanistic Bridge to Chronic Neuroinflammation

Cellular senescence is a heterogeneous, stress‐responsive state defined by durable changes in cell‐cycle control, chromatin organization, transcription and metabolism, often accompanied by a senescence‐associated secretory phenotype (SASP) (Chandra et al. 2012; Narita et al. 2003; Sadaie et al. 2013; Shah et al. 2013; Zhang et al. 2005). Its chromatin component is not a fixed histone code. Classical studies established senescence‐associated heterochromatin foci (SAHF) and retinoblastoma‐dependent repression of E2F targets, whereas genome‐wide analyses showed that repressive marks such as H3K9me3 and H3K27me3 are redistributed rather than uniformly gained or lost (Chandra et al. 2012; Narita et al. 2003; Zhang et al. 2005). The central issue is therefore not whether senescence adds a stereotyped histone signature, but how cells reorganize chromatin so that proliferation, stress responses and inflammatory transcription become durably reweighted.

This distinction is particularly important in AD, in which biological aging, metabolic dysfunction, proteostasis failure and chronic neuroinflammation coexist. A useful framework is to treat senescence as one route to chromatin persistence, rather than as the obligatory identity of every affected neural cell. In this view, the relevant unit is a stress‐stabilized chromatin state whose persistence depends on the interaction of nuclear architecture, enhancer activity, histone‐modification turnover and metabolic substrate supply (Figure 3, Table 2).

**FIGURE 3:**
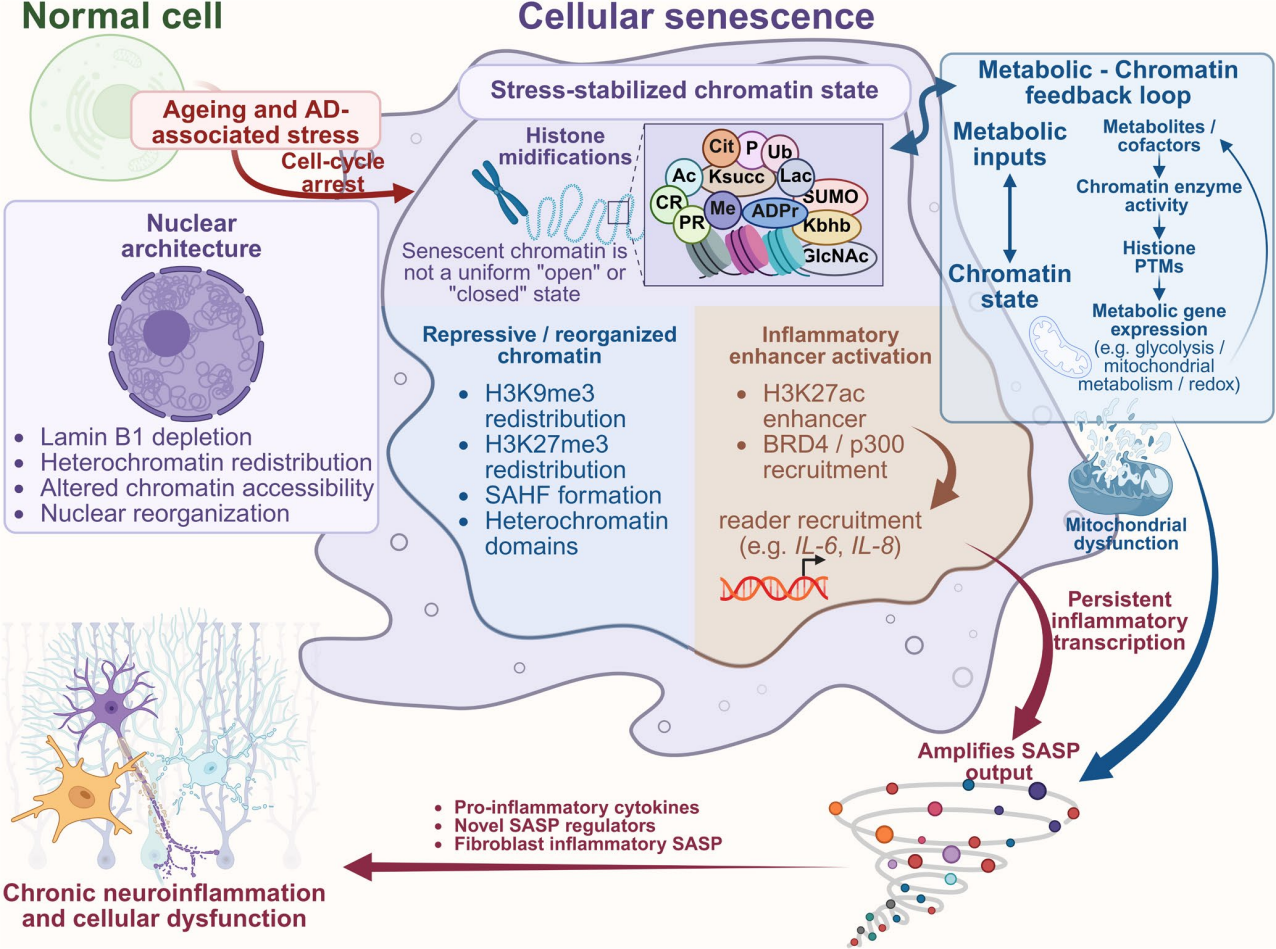
Stress‐stabilized chromatin states and metabolic feedback in aging and AD. This schematic depicts how aging and AD‐associated stress induces cell‐cycle arrest and cellular senescence, leading to a stress‐stabilized chromatin state. In this state, nuclear architecture is altered, characterized by Lamin B1 depletion, heterochromatin redistribution, altered chromatin accessibility, and nuclear reorganization. Senescent chromatin is not a uniform “open” or “closed” state, but rather encompasses combinatorial histone modifications, featuring both repressive/reorganized chromatin domains and inflammatory enhancer activation. A metabolic–chromatin feedback loop further amplifies this process, wherein metabolites and cofactors modulate chromatin enzyme activity, subsequently influencing histone PTMs and metabolic gene expression, leading to mitochondrial dysfunction. This persistent inflammatory transcription amplifies SASP output, ultimately driving chronic neuroinflammation and cellular dysfunction. This figure was created in BioRender.com.

**TABLE 2:** Chromatin and histone mechanisms in cellular senescence and metabolic state.

Chromatin mechanism (key regulator/mark)	Chromatin/molecular effect	Functional outcome	Experimental evidence	References
Heterochromatin formation (H3K9me3, H3K27me3, SAHF)	Repressive chromatin redistributes and compacts at heterochromatic and proliferation‐associated loci	Durable cell‐cycle arrest and senescence	Human fibroblasts; imaging, ChIP‐based and genomic analyses	Chandra et al. (2012), Narita et al. (2003), Sadaie et al. (2013), Shah et al. (2013), Zhang et al. (2005)
Histone supply and nucleosome loss	Lysosomal chromatin extrusion and reduced histone biosynthesis alter the histone template; telomere damage destabilizes telomeric chromatin	Loss of chromatin substrate alongside redistribution of existing marks	Human diploid fibroblasts; chromatin‐extrusion assays, histone quantification and telomere‐dysfunction models	Ivanov et al. (2013), O'Sullivan et al. (2010)
Gene‐body histone code (H3K79me3; H4R3me2s/2as)	H3K79me3 spreads across gene bodies, whereas H4R3me2s is replaced by H4R3me2as during replicative senescence	Gene upregulation and a functional histone signature of replicative senescence	Replicatively senescent fibroblasts; ChIP‐seq and perturbation of DOT1L/PRMT1/PRMT5/JMJD6	Suter et al. (2026)
Polycomb repression and non‐cell‐autonomous control (EZH2/H3K27me3; NOTCH)	EZH2/H3K27me3 represses selected senescence and inflammatory loci; NOTCH signaling between senescent and neighboring cells alters chromatin organization	Context‐dependent modulation of senescence and the SASP	Cellular senescence, immune and tumor models; genetic, pharmacological and co‐culture approaches	Chibaya et al. (2023), Ito et al. (2018), Parry et al. (2018), Zhang et al. (2018)
Histone variants and architectural proteins (H2A.J; HMGB2; macroH2A1)	H2A.J promotes inflammatory transcription; HMGB2 limits repressive chromatin spreading at SASP loci; macroH2A1 constrains paracrine senescence	Enhancement or braking of the SASP depending on the regulator	Human and mouse cells; knockdown, overexpression, ChIP‐seq and co‐culture	Aird et al. (2016), Chen et al. (2015), Contrepois et al. (2017)
SWI/SNF remodeling (ARID1A)	ARID1A deficiency attenuates KRAS‐induced senescence	Chromatin‐remodeller composition influences senescence entry	Pancreatic intraepithelial neoplasia and human cells; genetic loss‐of‐function	Liu et al. (2021)
SASP enhancer activation (H3K27ac/BRD4; NF‐κB)	H3K27ac marks inflammatory enhancers and BRD4 sustains enhancer‐driven transcription	Sustained SASP expression and paracrine signaling	Oncogene‐induced senescence; ChIP‐seq, transcriptional profiling and BRD4 inhibition	Chien et al. (2011), Tasdemir et al. (2016)
Enhancer licensing (HDAC4–AP‐1/p300; MLL1)	HDAC4 degradation releases an AP‐1/p300‐driven enhancer programme; MLL1 is required for SASP gene expression	Selective activation of senescent enhancer repertoires and SASP competence	Human and mouse senescent cells; HDAC4 and MLL1 perturbation and enhancer profiling	Capell et al. (2016), Di Giorgio et al. (2021)
Histone lactylation (H3K18la; H4K12la)	Lactate‐dependent histone lactylation links glycolytic state to transcription; H3K18la associates with inflammatory programmes in senescent microglia, whereas H4K12la participates in AD‐associated microglial glycolytic feedback	Context‐dependent inflammatory or metabolic state reinforcement	Activated macrophages; senescent microglia; 5xFAD models and human AD brain	Pan et al. (2022), Wei et al. (2023), Zhang et al. (2019)
Cytoplasmic chromatin–cGAS–STING and metabolic control	Mitochondrial dysfunction promotes CCF formation; CCFs activate cGAS–STING, whereas glucose limitation or metformin suppresses CCF formation through AMPK‐dependent control of ALIX	SASP amplification and metabolic control of innate immune activation	Human cells; mouse senescence models; aged mice	Dou et al. (2017), Kumazawa et al. (2026), Vizioli et al. (2020)
Acetyl‐CoA–H3K27ac coupling (mitochondrial metabolism; ACSS2)	Mitochondrial acetyl‐CoA supplies H3K27ac at SASP loci; ACSS2 couples acetate to neuronal histone acetylation	SASP execution and hippocampal memory	Senescent human cells; aged mice; mouse hippocampus	Martini et al. (2026), Mews et al. (2017)
NAD+–sirtuin signaling	SIRT1 restrains NF‐κB through RelA/p65 deacetylation and contributes to epigenetic control of the SASP; disrupted brain NAD+ homeostasis is associated with AD severity and is pharmacologically reversible in mouse models	Reversible metabolic constraint on inflammatory and AD‐associated states	Senescent cells; microglia; AD mouse models and human brain	Chaubey et al. (2026), Chen et al. (2005), Hayakawa et al. (2015), Xu et al. (2020)
Metabolite‐dependent acylation and dioxygenase activity	Crotonyl‐CoA and succinyl‐CoA support histone acylation; β‐hydroxybutyrate modulates HDAC activity and histone β‐hydroxybutyrylation; α‐ketoglutarate regulates 2‐oxoglutarate‐dependent dioxygenases	Metabolic control of chromatin modification and senescence plasticity	Human and mouse cells; biochemical and metabolic perturbation models	Akakura and Tabibzadeh (2026), Sabari et al. (2015), Shimazu et al. (2013), Wang et al. (2017), Xie et al. (2016)
LKRSDH‐dependent histone regulation	H3R17me2 and H3K27me3 jointly regulate dilp loci and contribute to age‐related circadian changes	Age‐associated transcriptional and behavioral change	*Drosophila* aging model; RNA‐seq and ChIP‐seq	Lv et al. (2024)
Redox–epigenetic coupling	Redox state alters chromatin‐enzyme activity and intersects with DNA‐damage signaling	Coupling of metabolic stress to chromatin remodeling	Human cells and experimental aging/neurodegeneration models	Balamurli et al. (2024), Wang, Zhu, et al. (2026)
Histone code complexity	Distinct histone proteoforms and modification combinations generate context‐dependent outputs, limiting interpretation of single‐mark measurements	Constraint on reductionist epigenome readouts	Human cells and integrative aging studies	Malone et al. (2026), Suter et al. (2026)

### 3.1 Establishment of Senescence‐Associated Chromatin States

The establishment of senescence involves large‐scale reorganization of chromatin architecture rather than a uniform gain or loss of individual histone modifications. In classical oncogene‐induced senescence, SAHF formation and retinoblastoma‐dependent chromatin remodeling contribute to repression of E2F target genes, whereas macroH2A‐containing chromatin and HIRA‐dependent histone deposition further support senescence‐associated heterochromatin domains (Narita et al. 2003; Zhang et al. 2005). Genome‐wide analyses nevertheless show that H3K9me3 and H3K27me3 can be redistributed into different nuclear compartments without a simple one‐to‐one relationship between the abundance of a repressive mark and physical compaction (Chandra et al. 2012). Senescent chromatin is therefore better understood as a spatially reorganized regulatory landscape than as a globally more repressive genome.

Loss of nuclear‐lamina integrity adds a structural layer to this transition. Lamin B1 depletion is accompanied by redistribution of heterochromatic domains, altered chromatin accessibility and region‐specific changes in histone modifications (Sadaie et al. 2013; Shah et al. 2013). The nucleosomal substrate itself also changes: senescent cells can extrude chromatin fragments that are processed through lysosomal pathways, while persistent telomere damage suppresses histone biosynthesis and destabilizes telomeric chromatin (Ivanov et al. 2013; O'Sullivan et al. 2010). These observations highlight an important interpretative point for AD studies: a change in bulk histone abundance can reflect altered histone supply or nuclear organization rather than a locus‐specific change in chromatin function.

Specific histone combinations further demonstrate the dependence of senescent chromatin on genomic context. In replicative senescence, loss of promoter‐associated H4R3me2s and redistribution of H3K79me3 across gene bodies accompany transcriptional changes; perturbation of DOT1L, PRMT1, PRMT5, or JMJD6 alters cellular lifespan and selected senescence‐associated gene responses (Suter et al. 2026). Thus, activating and repressive histone states can change simultaneously, and the same modification may have different consequences depending on the genomic compartment in which it is deposited.

Polycomb‐mediated repression provides another example. EZH2, the catalytic component of PRC2, deposits H3K27me3 and helps maintain transcriptionally repressed chromatin; during senescence, reduced EZH2 activity can promote locus‐specific H3K27me3 loss and induction of senescence‐associated genes (Ito et al. 2018). In other senescence‐associated contexts, EZH2 inhibition can instead reshape inflammatory secretory programmes (Chibaya et al. 2023). NOTCH‐mediated communication between senescent and neighboring cells can further alter heterochromatin organization and secretory output (Parry et al. 2018). In macrophages and microglia, Ezh2‐dependent H3K27me3 repression of Socs3 supports TLR‐driven inflammatory transcription (Zhang et al. 2018). The implication is that H3K27me3 loss or gain should never be interpreted in isolation from cell type, stimulus, and locus.

Histone variants, chromatin remodellers and architectural proteins add further layers. H2A.J accumulates during senescence and aging and promotes inflammatory gene expression, whereas HMGB2 maintains accessibility at selected SASP loci by limiting encroachment of repressive chromatin (Aird et al. 2016; Contrepois et al. 2017). ATP‐dependent chromatin remodeling also contributes to the establishment of senescence: loss of the SWI/SNF‐associated subunit ARID1A attenuates oncogene‐induced senescence (Liu et al. 2021). Together, these findings indicate that senescence is built through coordinated changes in histone modifications, histone supply, histone variants, nucleosome organization and chromatin‐remodeling activity rather than through a universal epigenetic endpoint.

### 3.2 Chromatin Control of the SASP

The SASP is a major route by which senescent cells alter their environment, but its composition varies with cell identity, inducing stimulus and duration (Chien et al. 2011). Persistent cytokine and chemokine production is therefore not simply the output of a single transcription factor. Instead, SASP expression emerges from the interaction between inflammatory signaling and a chromatin state that determines enhancer accessibility, transcriptional amplitude, and the capacity of inflammatory loci to remain active over time.

NF‐κB is a central regulator of many SASP components, but sustained SASP transcription also depends on enhancer remodeling. H3K27ac accumulation at inflammatory enhancers and BRD4 recruitment reinforce transcription, and BRD4 inhibition suppresses SASP expression and paracrine signaling (Chien et al. 2011; Tasdemir et al. 2016). Additional chromatin changes reinforce this state: HDAC4 degradation releases an AP‐1–p300 programme at selected enhancers and super‐enhancers, whereas MLL1 contributes to SASP gene expression (Capell et al. 2016; Di Giorgio et al. 2021). The combined evidence places enhancer activity, rather than promoter occupancy alone, at the centre of persistent inflammatory transcription.

Chromatin architecture can determine whether inflammatory loci remain accessible. HMGB2 selectively occupies SASP‐associated regions and limits repressive chromatin spreading, whereas H2A.J accumulation promotes inflammatory transcription (Aird et al. 2016; Contrepois et al. 2017). MacroH2A1 also participates in paracrine senescence and SASP regulation (Chen et al. 2015). Conversely, H3K27me3 can constrain inflammatory transcription in some contexts; in macrophages and microglia, Ezh2‐dependent H3K27me3 represses Socs3 and sustains TLR‐driven inflammatory programmes (Zhang et al. 2018). The same modification can therefore function as a brake or as part of an inflammatory state depending on the transcriptional network in which it is embedded.

Metabolite‐derived histone modifications provide a further route to persistent secretory states. Histone lactylation was first linked to glycolytic metabolism in activated macrophages, and H3K18 lactylation in senescent microglia has subsequently been associated with enhanced NF‐κB signaling and increased IL‐6 and IL‐8 production in aging‐ and AD‐related models (Wei et al. 2023; Zhang et al. 2019). Senescent cells can also generate cytoplasmic chromatin fragments after mitochondrial dysfunction; in experimental senescence, low‐dose histone deacetylase inhibition attenuates chromatin‐fragment formation and the SASP (Vizioli et al. 2020). These findings suggest that the SASP is stabilized by a network of chromatin and innate‐immune processes rather than by one histone event.

### 3.3 Metabolic Inputs Into the Senescence‐Associated Chromatin State

Senescence and metabolism are reciprocally coupled. Chromatin‐modifying enzymes depend on metabolic substrates, cofactors, and redox conditions, whereas chromatin states regulate metabolic gene programmes (Malone et al. 2026). Importantly, metabolite abundance measured in the whole cell is not equivalent to the concentration experienced by a nuclear enzyme. Subcellular compartmentalization, local production, and consumption, enzyme recruitment, and modification turnover all determine whether a metabolic perturbation reaches a particular chromatin substrate.

Mitochondrial carbon metabolism illustrates this principle. In senescent cells, a mitochondrial pyruvate‐citrate‐acetyl‐CoA pathway supports histone acetylation, including H3K27ac at SASP‐associated loci; inhibition of this pathway reduces chromatin accessibility and inflammatory gene expression (Martini et al. 2026). In post‐mitotic neurons, ACSS2 can couple acetate metabolism to local acetyl‐CoA production, histone acetylation and memory‐associated transcription (Mews et al. 2017). NAD+‐dependent sirtuin activity provides a complementary route: reduced SIRT1 abundance or activity is associated with enhanced acetylation at selected inflammatory loci and a stronger SASP, while microglial SIRT1 can also restrain NF‐κB through deacetylation of RelA/p65 (Chen et al. 2005; Hayakawa et al. 2015; Xu et al. 2020). The latter effects involve non‐histone substrates as well as histones and should not be conflated.

Glycolysis provides another route through lactate. In AD‐associated microglia, H4K12 lactylation participates in a positive‐feedback circuit involving PKM2 and glycolytic gene expression, whereas H3K18 lactylation is associated with senescence‐associated inflammatory transcription (Pan et al. 2022; Wei et al. 2023). Other metabolic pathways provide additional inputs: crotonyl‐CoA supports histone crotonylation, succinyl‐CoA supports histone succinylation, β‐hydroxybutyrate inhibits selected histone deacetylases and can support histone β‐hydroxybutyrylation, and α‐ketoglutarate regulates 2‐oxoglutarate‐dependent dioxygenase activity (Akakura and Tabibzadeh 2026; Sabari et al. 2015; Shimazu et al. 2013; Wang et al. 2017; Xie et al. 2016). These pathways illustrate the biochemical breadth of metabolic–chromatin coupling without implying that any single metabolite dictates a uniform chromatin state.

Metabolic effects on chromatin are also evident outside mammalian senescence. LKRSDH‐dependent changes in H3R17me2 and H3K27me3 at insulin‐like peptide loci contribute to age‐related circadian changes in *Drosophila*, illustrating how altered intermediary metabolism can reshape defined histone states at aging‐responsive loci (Lv et al. 2024). Redox metabolism can further modify the activity of chromatin enzymes and the cellular response to DNA damage (Balamurli et al. 2024). Histone‐based aging clocks provide a complementary systems‐level approach for estimating biological age, although their cell‐type‐specific utility in AD remains to be established Niu et al. (2026).

The relationship is bidirectional. Mitochondrial dysfunction, altered carbon flux and changes in NAD+, acyl‐CoA or lactate availability can change the biochemical environment of chromatin enzymes, while chromatin‐dependent transcription can feed back to metabolic programmes (Martini et al. 2026; Wang, Zhu, et al. 2026). Cytoplasmic chromatin fragments (CCFs) provide a further bridge between metabolic stress and innate immunity: CCFs can activate cGAS–STING and promote inflammatory signaling in senescence, while a recent Nature Aging study showed that glucose limitation or metformin suppresses CCF formation through AMPK‐dependent control of the ESCRT‐associated protein ALIX, reducing cGAS‐mediated inflammation in aged mice (Dou et al. 2017; Kumazawa et al. 2026). In AD, where mitochondrial dysfunction, impaired energy metabolism and neuroinflammation coexist, this coupling provides a plausible route for stabilizing stress responses (Chaubey et al. 2026; Wang, Zhu, et al. 2026). Whether the same circuitry operates in specific CNS cell types and AD stages remains an open question.

### 3.4 Senescence‐Associated Chromatin States in the Aging and AD Brain

Evidence for senescence‐associated states in the CNS has increased substantially, but the evidence is strongest in glial compartments and should not be generalized to every neural cell. In human AD brain, single‐nucleus transcriptomic and imaging analyses identified the most complete canonical senescence programme in microglia within the cell types examined, with more partial senescence‐associated changes in oligodendroglia and astrocytes and little evidence for a canonical programme in neurons (Fancy et al. 2024). Experimental studies independently identified senescent TREM2‐expressing microglia in aging and AD models and a senescence‐like microglial subset within tau‐associated disease‐associated microglia (Ng et al. 2023; Rachmian et al. 2024). By contrast, evidence for a canonical senescence programme in post‐mitotic neurons remains limited (Wu, Sun, and Chow 2024). The field therefore has stronger support for senescence‐associated glial states than for a universal neuronal senescence state.

Microglia provide the clearest connection between senescence‐associated states and chronic neuroinflammation. Human microglial models exposed to mitochondrial proteotoxic stress show metabolic rewiring, S‐adenosylmethionine depletion and a senescent phenotype with altered inflammatory communication (Perez J et al. 2026). In parallel, recent work shows that aging‐associated loss of the histone chaperone DAXX promotes chromatin decompaction at retrotransposable elements, microglial reactivity, cell‐cycle re‐entry and DNA damage, followed by the emergence of microglia with senescence‐associated features; sustained senescence in this model depends on promyelocytic leukemia protein (Yan et al. 2026). These findings place mitochondrial stress and heterochromatin maintenance upstream of persistent microglial dysfunction, while leaving open whether one conserved histone signature links these processes across human AD.

Aging also alters the chromatin landscape on which AD pathology develops. Single‐cell epigenomic work in the aging mouse brain has identified cell‐type‐specific loss of heterochromatin integrity and dysfunction of lineage transcription factors, while recent single‐cell multi‐omic analysis has shown that transposon demethylation and three‐dimensional genome remodeling occur in a cell‐type‐specific manner during brain aging (Amaral et al. 2026; Zeng et al. 2026). Such changes matter because retroelement derepression, altered enhancer accessibility and changes in genome architecture can modify the threshold at which inflammatory or stress‐responsive genes are activated. They should, however, be distinguished from senescence‐specific alterations unless the relevant senescence phenotype is independently established.

Neuronal chromatin instability provides a second route to chronic glial activation. Chronic loss of neuronal heterochromatin can derepress endogenous retroelements and provoke reactive gliosis, whereas tau‐associated chromatin relaxation and loss of piRNA‐mediated repression connect proteinopathy to neuronal genome regulation (Frost et al. 2014; Newman et al. 2025; Sun et al. 2018). Border‐associated macrophages can also induce paracrine microglial senescence, indicating that senescence‐associated states may propagate through cell–cell signaling rather than arise exclusively within the initially stressed cell (Hu et al. 2025). Evidence for H3K9me3 loss and retroelement activation in peripheral aging tissues provides mechanistic precedent but should not be treated as direct evidence for an AD‐specific CNS pathway (Liu, Molchanov, et al. 2025).

Astrocytes undergo substantial aging‐ and disease‐associated transcriptional remodeling, but direct evidence that a defined histone PTM establishes a stable astrocytic senescence programme in AD remains limited (Fancy et al. 2024; Perez J et al. 2026). Neurons are even more distinct because they are post‐mitotic and do not undergo classical proliferative senescence; senescence‐like neuronal phenotypes should therefore be separated conceptually from canonical cell‐cycle arrest (Wu, Sun, and Chow 2024). Senescence is thus best considered one component of the aging brain chromatin landscape, not a synonym for all persistent histone reprogramming in AD.

Taken together, the evidence supports a model in which senescence contributes to, but does not fully define, persistent chromatin dysfunction in the aging and AD brain. The transition from transient stress responses to durable inflammatory states appears to require coordination among chromatin architecture, enhancer regulation, histone turnover, metabolic state, and innate immune sensing. The major unresolved question is which of these features are causal and persistent within specific human CNS cell types, rather than merely correlated with disease stage or altered cellular composition.

## 4 Cell‐Type‐Specific Histone Reprogramming in AD

Histone modifications are not deployed uniformly across the CNS. Each cell type has a distinct repertoire of lineage‐defining transcription factors, enhancers, writers, erasers, and readers, as well as distinct metabolic and inflammatory inputs. Consequently, the same PTM can mark a reparative state in one cell and a persistent inflammatory state in another. Cell type is therefore not a descriptive annotation added after the fact; it is a determinant of how a histone modification is written, interpreted, and functionally propagated (Figure 4) (Butovsky et al. 2014; Gosselin et al. 2017; Green et al. 2024; Hammond et al. 2019; Lavin et al. 2014; Saeki and Ozato 2025).

**FIGURE 4:**
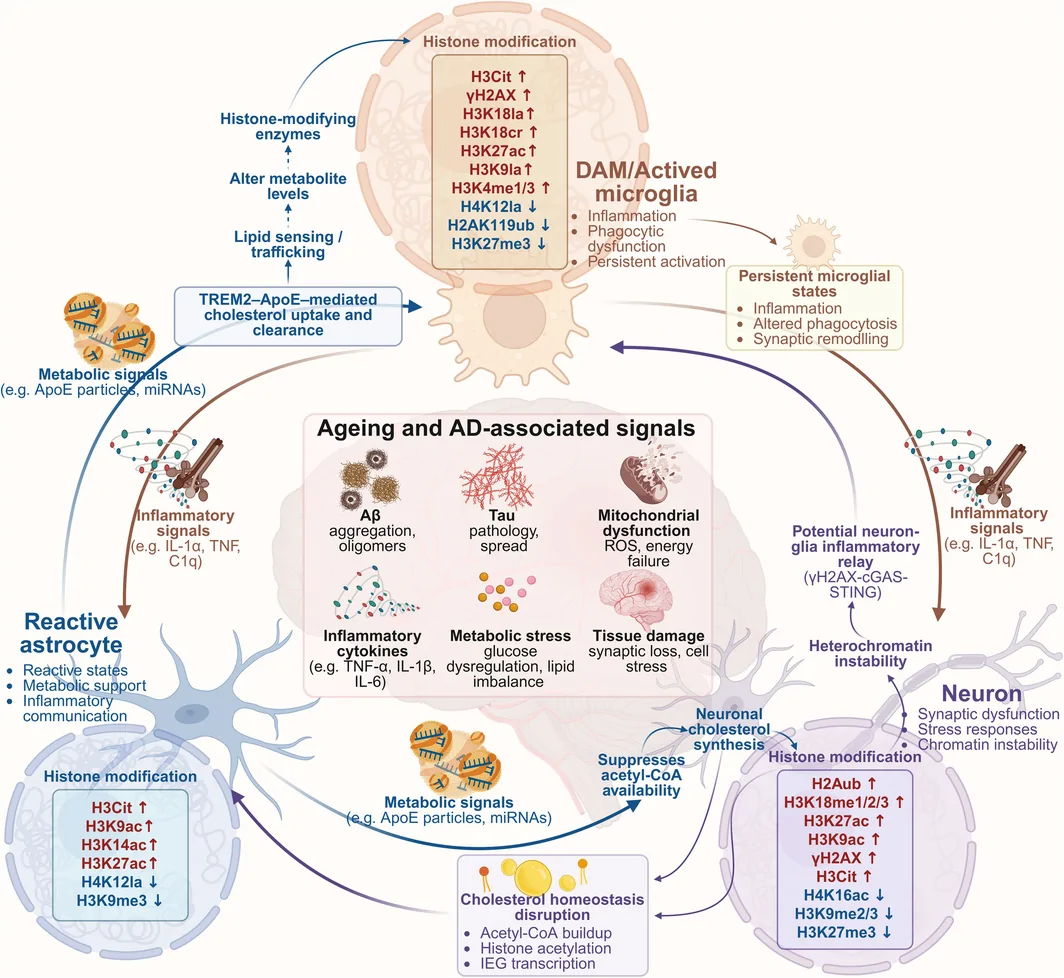
Cell‐type‐specific epigenetic remodeling and neuroinflammatory crosstalk in aging and AD. Aging and AD‐associated signals—including Aβ aggregation, Tau pathology, mitochondrial dysfunction, and metabolic stress—drive cell‐type‐specific epigenetic remodeling and neuroinflammatory crosstalk among microglia, astrocytes, and neurons. These signals induce distinct shifts in activating and repressive histone modification landscapes across these cell types, promoting persistent microglial reactivity, altered astrocytic metabolic communication, and neuronal synaptic dysfunction and chromatin instability. Mechanistically, metabolic signals and TREM2–ApoE‐mediated cholesterol uptake modulate histone‐modifying enzymes, while a neuro‐glial inflammatory relay induces heterochromatin instability. Neuronal cholesterol synthesis suppresses acetyl‐CoA availability, disrupting histone acetylation and immediate early gene (IEG) transcription. Ultimately, these interconnected epigenetic and metabolic feedback loops perpetuate neuroinflammation and neurodegeneration. This figure was created in BioRender.com.

### 4.1 Microglia: Highly Plastic Chromatin States Under Inflammatory and Metabolic Stress

Microglia provide the strongest example of context‐dependent chromatin plasticity in AD. Homeostatic identity is maintained by an enhancer network involving PU.1, SALL1, and MEF2C and reinforced by TGF‐β‐dependent signaling (Butovsky et al. 2014; Gosselin et al. 2017; Hammond et al. 2019; Saeki and Ozato 2025). The surrounding tissue environment is integral to this regulatory state: human and mouse microglia progressively remodel their transcriptional programmes when removed from the CNS or exposed to injury‐associated cues (Gosselin et al. 2017; Hammond et al. 2019; Lavin et al. 2014). In primary human microglia from 150 donors, transcriptomic and chromatin‐accessibility analyses linked genetic variation at 21 ad risk loci to microglial regulatory networks and refined several loci to candidate target genes, placing AD susceptibility within a cell‐specific regulatory context rather than treating risk variants as isolated expression changes (Kosoy et al. 2022).

Spatial context adds another layer of complexity. Integrated fate mapping, single‐cell transcriptomics, epigenomic profiling, and histology in an AD mouse model showed that plaque‐associated and non‐plaque‐associated microglia occupy distinct, dynamic states with different chromatin‐accessibility profiles and can transition during amyloid deposition (Ardura‐Fabregat et al. 2025). This finding has an important methodological implication: bulk measurements of histone PTMs cannot readily distinguish a true within‐cell change from altered cell composition or state redistribution. Cell‐type‐resolved and spatially resolved chromatin profiling is therefore essential for defining AD‐associated histone states.

Inflammatory activation remodels enhancer‐associated histone marks. H3K27ac is consistently implicated in microglial enhancer activation, while H3K4me1 provides an additional indicator of enhancer priming (Huang et al. 2023; Wendeln et al. 2018). In the experimental brain‐training paradigm, inflammatory priming produced long‐lasting H3K4me1 changes together with altered H3K27ac patterns, and microglia‐specific Hdac1/2 deletion attenuated the trained response (Wendeln et al. 2018). These findings support durable enhancer reprogramming as a mechanism of inflammatory memory. Equivalent persistent histone memory after amyloid‐β exposure in AD, however, remains to be established; current evidence is stronger for state‐dependent enhancer remodeling than for a validated amyloid‐specific histone memory programme (Nirakis et al. 2025; Valiukas et al. 2025).

Histone lactylation illustrates the same principle at a different biochemical layer. In aging‐associated senescent microglia, H3K18 lactylation is associated with NF‐κB activation and SASP‐factor production (Wei et al. 2023). In AD itself, H4K12la is elevated in plaque‐adjacent microglia in both 5xFAD mice and human AD brain, is enriched at promoters of glycolytic genes, and participates in a positive‐feedback circuit linking glycolysis, histone lactylation, and PKM2; pharmacological inhibition of PKM2 attenuates microglial activation, whereas microglia‐specific *Pkm2* ablation improves spatial learning and memory in 5xFAD mice (Pan et al. 2022). By contrast, H3K18la can support a neuroprotective microglial programme through Plxnb2 after ischaemic stroke, whereas H4K12la promotes PD‐1 transcription and facilitates microglial repair after spinal cord injury (Hu et al. 2024; Li, Wang, et al. 2025; Wang, Zhou, et al. 2025). In cerebral ischaemia, microglial SMEK1 deficiency increases lactate production through the PDK3–PDH pathway, elevates H3K9la, and promotes transcription of glycolytic genes such as Ldha and Hif1α, providing a direct example of a metabolic feedback loop in which a change in glycolytic flux is linked to a site‐specific histone modification (Si et al. 2024). Conversely, oxygen–glucose deprivation has been associated with H3K9la‐dependent pro‐inflammatory programmes in microglia, and a Parkinson disease model linked increased glycolysis‐derived lactate to microglial activation through histone lactylation (He et al. 2024; Qin et al. 2025). These observations argue against assigning a fixed biological direction to lactylation as a modification class.

A recent spinal cord injury study further emphasizes why cell identity must be specified. Lactate accumulation increased H3K9la in lesion‐infiltrating macrophages and circulating monocytes rather than resident microglia. Integrated CUT&Tag and RNA‐seq implicated TXNIP as a direct H3K9la‐associated target and connected this axis to NLRP3 activation, mitochondrial dysfunction and tissue inflammation; selective inhibition of macrophage H3K9la improved functional recovery (Shi et al. 2026). This study provides strong mechanistic evidence for a lactate–histone–inflammation pathway, but in a distinct myeloid compartment and disease context.

Repressive chromatin can also constrain inflammatory programmes in microglia. In an adolescent depression model, microglial PCGF1 overexpression increased RING1B/H2AK119ub and EZH2/H3K27me3 occupancy at the MMP10 promoter, suppressing MMP10 transcription and attenuating microglia‐mediated neuroinflammation. This study illustrates that increased deposition of repressive marks can be protective in one inflammatory context and that their effects depend on the genomic target rather than on the modification alone (Li, Du, et al. 2025).

Microglial lipid metabolism adds another layer of state control. The TREM2‐APOE axis regulates lipid sensing and disease‐associated microglial programmes, and disturbed lipid trafficking is linked to metabolically stressed states with altered phagocytic and homeostatic function (Cheng and Ho 2025; Jung et al. 2025; Krasemann et al. 2017; Sprenger et al. 2025; Traetta et al. 2025). Changes in lipid handling also modify the metabolic context in which acetyl‐CoA, NAD+, S‐adenosylmethionine, α‐ketoglutarate, and lactate are generated or consumed (Jung et al. 2025; Rahimpour et al. 2024). The resulting interaction between lipid state and chromatin is an important candidate mechanism because microglial metabolic transitions can change both the substrate supply to histone enzymes and the transcription factors that recruit those enzymes.

Other histone‐associated modifications expand the repertoire. Microglial H3K18 crotonylation is increased in 5xFAD and Aβ42‐injected mouse models, localizes predominantly to microglia and promotes STAT1 expression with downstream inflammatory and cognitive phenotypes (Weng et al. 2026). In a Parkinson disease model, γH2AX was linked to an α‐synuclein–STING–type I interferon axis, but this should be interpreted as disease‐specific evidence rather than as proof of an analogous AD neuronal pathway (Wang, Sun, et al. 2025). Likewise, H3Cit detected after stroke can be delivered by neutrophil extracellular traps and act extracellularly on microglia through AIM2, rather than representing a resident microglial nuclear programme (Chen, Ni, et al. 2025; Seol et al. 2025). The source, compartment, and molecular substrate of a histone PTM are therefore essential to interpreting its function.

Aging resets the baseline on which these microglial programmes operate. Age‐related loss of heterochromatin integrity and lineage‐factor function can precede disease‐associated state transitions, while senescent microglia can overlap phenotypically with disease‐associated microglia in experimental tauopathy (Amaral et al. 2026; Ng et al. 2023; Rachmian et al. 2024). Human microglial xenograft studies further show that APOE isoforms can reshape the transcriptional and epigenomic landscape of human microglia in an AD brain environment (Murphy, Hu, et al. 2025). These observations support a model in which aging does not simply add inflammatory stimuli; it alters the chromatin and metabolic starting state from which microglia respond to amyloid, tau, lipids and tissue damage.

Persistence is the critical criterion for distinguishing adaptive activation from epigenetic memory. Acute enhancer activation can support debris clearance and repair, whereas chronic disease‐associated stimulation can maintain complement activity, cytokine production, and synaptic remodeling (Parkhurst et al. 2013; Wang et al. 2020). Experimental immune training can leave durable histone‐associated changes in microglia, and APOE isoforms can reshape the transcriptional and chromatin‐accessibility landscape of human microglia engrafted into an AD mouse brain (Murphy, Hu, et al. 2025; Wendeln et al. 2018). Neither study, however, establishes that an AD‐associated histone PTM persists after removal of the initiating stimulus. Demonstrating such persistence in human AD‐relevant microglia remains a major experimental opportunity.

The same principle extends beyond resident microglia to other immune cells at the CNS interface. In experimental autoimmune encephalomyelitis, pharmacological inhibition of EED reduced H3K27me3 in dendritic cells, derepressed WNT pathway genes, and suppressed dendritic‐cell migration, thereby alleviating neuroinflammation. This study is not an AD model, but it illustrates how cell‐specific Polycomb states can regulate inflammatory trafficking in the CNS environment (Hong et al. 2025).

### 4.2 Astrocytes: Reactive Chromatin States and Intercellular Metabolic Control

Astrocytes undergo extensive transcriptional and metabolic remodeling during neuroinflammation, but the chromatin logic of these states is less completely defined than in microglia. Reactive astrocytes are heterogeneous and shaped by local tissue signals, including microglial cytokines such as IL‐1α, TNF, and C1q (Lee et al. 2024; Li, Huo, et al. 2025; Liddelow et al. 2017; Morales Pantoja and Mintz 2026; Wang, Qiao, et al. 2025). Microglia‐induced pathological conversion therefore provides an important model for asking whether glial reactivity can be encoded beyond the initiating inflammatory signal.

Histone acetylation is among the best‐characterized chromatin features of reactive astrocytes. Microglia‐induced pathological conversion has been associated with a stable increase in H3K9/K14ac, a transient increase in H3K27ac and a reduction in H3K9me3 (Villarreal et al. 2021). This temporal separation is informative: not every activation‐associated histone change is equally persistent, and stable H3K9/K14ac does not by itself establish a senescent state. Pharmacological HDAC inhibition can modify glial inflammatory responses, but these interventions affect both histone and non‐histone substrates and therefore require cautious mechanistic interpretation (Mancino et al. 2025; Pinheiro, Diniz, et al. 2025). Importantly, the consequences of HDAC modulation differ across neural progenitors, mature neurons and glial populations, so cell‐type‐aware dosing is required in the astrocyte compartment as elsewhere (Diniz et al. 2025). A primary study in cultured astrocytes and in mice receiving intracerebroventricular Aβ oligomers showed that HDAC inhibition (LASSBio‐1911) reduces astrocyte reactivity, enhances astrocytic synaptogenic potential and improves synaptic and memory outcome (Diniz et al. 2024).

Astrocytes can also regulate neuronal chromatin indirectly through metabolic coupling. Astrocyte‐derived ApoE particles deliver microRNAs that suppress neuronal cholesterol biosynthesis, increasing acetyl‐CoA availability and promoting neuronal histone acetylation and immediate‐early‐gene transcription; ApoE4 is less effective than ApoE3 in this pathway (Li, Zhang, et al. 2021). This is a striking example of an intercellular metabolic‐to‐chromatin relay: the cell that changes the metabolic environment is not the cell in which the histone modification is ultimately measured. Broader work on cell‐type‐specific brain cholesterol metabolism places this pathway within a larger division of metabolic labor across neural cell types (Li, Zhang, and Liu 2022).

Astrocytic epigenetic memory has been demonstrated in experimental CNS autoimmunity. Disease‐associated astrocytes retain a reactivatable inflammatory programme in which ACLY‐derived acetyl‐CoA and p300‐dependent chromatin regulation contribute to inflammatory transcription (Lee et al. 2024). Because this work derives from experimental autoimmune encephalomyelitis and related CNS inflammatory settings rather than AD, it should be regarded as a mechanistic precedent for persistence rather than direct evidence of an AD astrocyte programme. A further example comes from experimental subarachnoid hemorrhage, in which BRD4‐dependent regulation of histone lactylation, particularly H4K8la, influences astrocyte polarization; silencing BRD4 reduces H4K8la and aggravates A1‐like polarization and neuronal injury (Zhang, Zhou, Lu, et al. 2024). Comparable cell‐type‐resolved evidence in AD remains sparse (Hu et al. 2026).

Metabolic regulation of astrocytic chromatin provides a complementary mechanistic link. In LPS‐stimulated astrocytes and an MPTP Parkinson disease model, PHGDH‐dependent serine synthesis sustains NADH and thereby facilitates histone acetylation at inflammatory cytokine promoters; PHGDH inhibition reduces promoter acetylation and pro‐inflammatory cytokine production (Lv et al. 2025). Similarly, in LPC‐induced demyelination, SIRT6 restrains astrocytic H3K9ac and CHI3L1 expression; SIRT6 inhibition increases H3K9ac occupancy at the CHI3L1 promoter and worsens remyelination, whereas astrocyte‐specific SIRT6 overexpression has the opposite effect (Du et al. 2024). These examples show that metabolic and deacetylase pathways can converge on astrocytic histone acetylation, while also underscoring that the relevant causal chain may be pathway‐ and locus‐specific.

### 4.3 Neurons: Activity‐Dependent Histone Regulation, Inflammatory Stress and Chromatin Instability

Neurons are post‐mitotic and therefore do not undergo classical proliferative senescence, yet they are highly dependent on dynamic chromatin regulation. Their relevant phenotypes include altered activity‐dependent transcription, changes in histone acetylation, DNA‐damage‐associated histone phosphorylation, and loss of heterochromatic integrity. Senescence‐like neuronal features may occur under selected pathological conditions, but these should not be equated with canonical cell‐cycle arrest (Wu, Kupferschmid, et al. 2025).

Activity‐dependent chromatin regulation is a physiological baseline against which pathological changes should be interpreted. Learning and synaptic stimulation recruit CREB, CBP/p300, MEF2 and SRF to immediate‐early and plasticity‐associated genes, while neuronal enhancers acquire activating histone acetylation that supports rapid transcriptional responses (Chen et al. 2019; Khan, Carretero‐Rey, et al. 2025; Malik et al. 2014; Singh and Paramanik 2024). HDAC2 elevation, by contrast, is associated with repression of plasticity‐associated genes and impaired cognitive function in experimental neurodegeneration (Asadi et al. 2025; Chen et al. 2019; Gräff et al. 2012; Malik et al. 2014; Singh and Paramanik 2024). These observations emphasize that increased acetylation is not intrinsically pathological; its functional effect depends on genomic location, neuronal state and the transcriptional programme engaged.

Neuronal chromatin is also shaped by glial metabolic signals. Astrocyte‐derived ApoE‐dependent suppression of neuronal cholesterol synthesis increases acetyl‐CoA availability and promotes neuronal histone acetylation, including H3K27ac at immediate‐early genes (Li, Zhang, and Liu 2022; Li, Zhang, et al. 2021). A separate lead‐exposure model showed that sodium butyrate increased ACSS2‐dependent H3K9ac and BDNF expression and improved neuroinflammatory and cognitive phenotypes (Li, Liu, et al. 2024). These studies illustrate two different routes to neuronal acetylation: intercellular metabolic coupling and pharmacological modulation of acetate utilization. Neither should be assumed to represent the dominant pathway in AD without cell‐type‐specific validation.

Neuronal histone citrullination provides an additional example of metabolic–epigenetic coupling that is mechanistically distinct from inflammatory CitH3 released by neutrophils. After ischaemic stroke, PLA2G2E‐derived lipid metabolism generates DGLA in peri‐infarct neurons, increases PADI4 expression and promotes histone citrullination‐associated transcription of recovery genes; scRNA‐seq and epigenetic profiling linked this axis to endogenous neural repair (Nakamura et al. 2023). This observation is particularly informative for the present framework because it connects a lipid‐derived signal to a neuronal histone PTM and a functional outcome, while also illustrating that histone citrullination can be reparative rather than uniformly inflammatory.

DNA‐damage‐associated histone marks provide another potential bridge between neuronal stress and neuroimmune signaling. γH2AX is a canonical marker of DNA double‐strand break responses in stressed neurons (Rahman et al. 2025; Scheijen et al. 2025). Microglial cGAS deletion and pharmacological STING blockade each alleviate AD‐associated phenotypes in experimental models, establishing the relevance of cGAS‐STING signaling in AD but not proving that neuronal γH2AX directly activates this pathway (Chung et al. 2024; He et al. 2025). The mechanistic gap is therefore precise: the field needs experiments that connect a defined neuronal DNA‐damage state to cytosolic DNA sensing, cell–cell signaling and downstream microglial responses (Wang, Sun, et al. 2025).

Heterochromatin is similarly vulnerable to aging and proteinopathy. H3K9me2, H3K9me3 and H3K27me3 contribute to repression of inappropriate programmes and genome stability, whereas aging and neurodegeneration can alter the distribution and integrity of these repressive states (Amaral et al. 2026; Katbe et al. 2026; Kumar and Beck 2025; Newman et al. 2025). Loss of heterochromatic control can derepress transposable elements, while tau pathology has been linked to global chromatin relaxation and altered histone acetylation (Frost et al. 2014; Klein et al. 2019; Kumar and Beck 2025; Min et al. 2015; Sun et al. 2018). These changes provide a plausible route by which proteinopathy can convert a normally plastic neuronal epigenome into a persistently dysregulated state.

Human AD brain studies provide the strongest evidence that disease‐associated histone reprogramming is not synonymous with cellular senescence. Regional loss of H4K16ac has been reported in AD, integrated multi‐omics studies identify gains in H3K27ac and H3K9ac, and histone acetylome‐wide analyses identify AD‐associated H3K27ac differences in the entorhinal cortex (Marzi et al. 2018; Nativio et al. 2018, 2020). Tau pathology is likewise associated with large‐scale changes in histone acetylation, and experimental manipulation of histone acetylation can modify tau‐mediated neurodegeneration (Klein et al. 2019; Min et al. 2015). A 2026 single‐nucleus epigenomic atlas of the adult human CNS further demonstrated strong cell‐type specificity of H3K27ac and H3K27me3 and identified primed, developmentally reminiscent chromatin states in adult oligodendroglia and astrocytes, but not microglia (Kabbe et al. 2026). This resource is not an AD cohort, but it reinforces the need to interpret disease‐associated chromatin through cellular identity. Together, these findings argue that human AD chromatin should be interpreted through cell type, regional anatomy and disease context rather than through a single global histone signature.

Histone H2A ubiquitination provides an additional chromatin link between DNA damage and proteostatic stress. In Brap loss‐of‐function mouse brain, increased H2A ubiquitination accompanies persistent DNA damage, altered proteostasis, senescence and neuroinflammation, and elevated H2A ubiquitination has also been detected in human AD brain tissue (Guo et al. 2022). The experimental model supports a mechanistic connection between H2A ubiquitination and aging‐associated pathology, whereas the human AD finding remains associative.

A final layer is provided by PTM diversity. A 2026 study of G9a inhibition with the SAM‐competitive compound FLAV‐27 showed therapeutic effects in both SAMP8 and 5xFAD models, accompanied by multi‐omic reprogramming of H3K9me2/H3K18me1–3‐associated repression (Bellver‐Sanchis et al. 2026). The study strengthens the case that histone methylation is functionally relevant in AD models, but because G9a regulates multiple genomic targets, the causal contribution of any individual histone residue remains unresolved. Similarly, recent PD work identified CBP/p300‐dependent H3K27ac at the PDLIM7 locus as a regulatory node that mitigates neuroimmune dysregulation in 6‐OHDA models (Zhang and Yin 2026). Such studies broaden the mechanistic landscape while reinforcing the need to separate direct site‐level causality from pharmacological or pathway‐level effects.

Neuronal chromatin is therefore best viewed as an emergent component of a multicellular regulatory system in which glial state, neurotrophic signaling and lipid metabolism can reshape neuronal transcription and synaptic function (Li, Zhang, and Liu 2022; Li, Zhang, et al. 2021; Liddelow et al. 2017; Parkhurst et al. 2013). Across microglia, astrocytes and neurons, the available evidence converges on three principles. First, the same PTM can support inflammatory, reparative or adaptive programmes depending on residue, locus, cell identity and disease context. Second, metabolic and extracellular signals can impose chromatin states across cellular boundaries, so cell‐autonomous measurements may underestimate the contribution of neuron–glia coupling. Third, the field still lacks sufficient locus‐resolved and longitudinal evidence to determine which disease‐associated PTM states persist after the initiating stimulus is removed. Future studies should therefore combine single‐cell or spatial chromatin profiling with metabolic tracing and cell‐type‐specific genetic perturbation to distinguish state‐associated marks from causal, persistent chromatin mechanisms in AD (Table 3).

**TABLE 3:** Representative site‐specific histone modifications associated with inflammatory, reparative and maladaptive states in CNS disease.

Histone PTMs/site	Disease model/perturbation	Change	Cellular source/compartment	Functional consequence	References
H3K18la	MCAO mouse model	↑	Microglia	Promotes a neuroprotective programme involving Plxnb2 and attenuates neuroinflammation	Li, Wang, et al. (2025)
H4K8la	SAH mouse model; astrocytic BRD4 silencing	↓ after knockdown	Astrocytes	Aggravates A1‐like polarization, neuronal injury and impaired recovery	Zhang, Zhou, Lu, et al. (2024)
H3K9la	MCAO/R; microglial SMEK1 deficiency	↑	Microglia	Activates glycolytic gene transcription and reinforces lactate–glycolysis feedback	Si et al. (2024)
H4K12la	Spinal cord injury; lactate treatment	↑	Microglia	Induces PD‐1 transcription and promotes microglia‐mediated repair	Hu et al. (2024), Wang, Zhou, et al. (2025)
H3K9la	Spinal cord injury; lactate accumulation	↑	Lesion‐infiltrating macrophages and circulating monocytes	Activates TXNIP–NLRP3 signaling, mitochondrial dysfunction and inflammation	Shi et al. (2026)
H3K27me3	Adolescent depression model; microglial PCGF1 overexpression	↑	Microglia	Represses MMP10 and attenuates neuroinflammation and depressive‐like behavior	Li, Du, et al. (2025)
H3K27me3	Experimental autoimmune encephalomyelitis; EED inhibition	↓	Dendritic cells	Upregulates WNT pathway genes, reduces dendritic‐cell migration and alleviates CNS inflammation	Hong et al. (2025)
H3K9me2/H3K18me	SAMP8 and 5xFAD; FLAV‐27	↓ after FLAV‐27	Brain tissue	Reprogrammes G9a‐associated H3K9me2/H3K18me repression and improves memory and synaptic phenotypes	Bellver‐Sanchis et al. (2026)
H3K9ac/H3K27ac	LPS‐stimulated astrocytes; PHGDH inhibition	↓	Astrocytes	Reduces acetylation at Il1b and Il6 regulatory regions and suppresses inflammatory cytokine production	Lv et al. (2025)
H3K9ac	Lead‐exposed mouse model; sodium butyrate	↑	Neural tissue	Restores BDNF expression and improves neuroinflammatory and cognitive phenotypes	Li, Liu, et al. (2024)
H3K4me1/H3K27ac	Inflammatory training; LPS‐primed microglia	Altered, durable	Microglia	Supports long‐lasting inflammatory priming; Hdac1/2 deletion attenuates the trained response	Wendeln et al. (2018)
H3K9ac/H3K56ac	LPC‐induced demyelination; SIRT6 perturbation	Altered	Astrocytes	Regulates astrocytic CHI3L1 through H3K9ac and modulates the lesion microenvironment and remyelination	Du et al. (2024)
H3K27ac	6‐OHDA Parkinson disease model; CBP/p300 activation	↑ at target locus	Dopaminergic neurons/substantia nigra	Activates PDLIM7 transcription and attenuates neuroimmune dysregulation	Zhang and Yin (2026)
Histone citrullination	Ischaemic stroke mouse model	↑	Peri‐infarct neurons	Promotes transcription of recovery‐associated genes downstream of the PLA2G2E–DGLA–PADI4 pathway	Nakamura et al. (2023)
H3Cit	tMCAO; NET formation	↑ extracellular	NETs/extracellular space	Engages AIM2‐dependent microglial pyroptosis and post‐stroke inflammation	Chen, Ni, et al. (2025)
H2Aub	Brap loss‐of‐function mouse brain; human AD cortex	↑	Cortical tissue	Links persistent DNA damage to proteostasis defects, senescence and neuroinflammation; elevated in human AD tissue	Guo et al. (2022)
H2AX Ser139 (γH2AX)	Rotenone‐induced Parkinson disease model	↑	Neuronal/brain tissue	Associated with the α‐synuclein–STING–type I interferon axis	Wang, Sun, et al. (2025)
H3K18cr	5xFAD and Aβ42‐injected mouse models	↑	Microglia	Promotes STAT1 expression, inflammatory activation and cognitive dysfunction	Weng et al. (2026)

## 5 Metabolic Control of Histone Post‐Translational Modifications

Metabolism regulates histone PTMs by supplying substrates and cofactors to modifying enzymes and by altering the biochemical conditions that determine their activity. This relationship is not deterministic. Whole‐cell metabolite abundance does not necessarily predict the nuclear pool, and substrate availability alone does not determine which genomic loci will be modified. Enzyme recruitment, compartmentalization, chromatin accessibility and modification turnover together determine the transcriptional output (Malone et al. 2026; Martini et al. 2026; Wang, Zhu, et al. 2026). The most useful way to interpret metabolic‐epigenetic coupling in AD is therefore as a set of constrained biochemical routes, each of which must be validated at the level of cell type, compartment and locus.

### 5.1 Acetyl‐CoA Supply and Histone Acetylation

Acetyl‐CoA integrates glucose oxidation, mitochondrial citrate export, acetate metabolism and other carbon sources. In senescent cells, mitochondrial pyruvate‐citrate‐acetyl‐CoA metabolism sustains histone acetylation at SASP‐associated loci; inhibition of this pathway reduces H3K27ac, chromatin accessibility and inflammatory gene expression (Martini et al. 2026). This study is important because it links a defined metabolic route to a chromatin phenotype and a downstream inflammatory programme rather than simply correlating metabolite abundance with histone acetylation.

Nuclear acetate metabolism provides a complementary route. ACSS2 can localize to chromatin in neurons, increase local acetyl‐CoA availability and support histone acetylation required for long‐term memory (Mews et al. 2017). In astrocyte‐neuron communication, ApoE‐dependent suppression of neuronal cholesterol synthesis similarly increases acetyl‐CoA availability and neuronal histone acetylation (Li, Zhang, et al. 2021). These findings demonstrate that acetyl‐CoA pools are functionally compartmentalized and can be supplied by different metabolic routes in different cell types.

For AD, this distinction matters because increased acetyl‐CoA is not equivalent to a pathogenic acetylation state. A causal mechanism requires identification of the acetyltransferase, the modified genomic loci, and the transcriptional programme that links the metabolic change to disease‐relevant phenotypes. The same metabolic intervention could also change protein acetylation, mitochondrial function, or ATP balance independently of histone regulation.

### 5.2 Lactate Metabolism and Histone Lactylation

Histone lactylation provides a direct biochemical route through which glycolytic metabolism can influence chromatin. Lactylation was first described in activated macrophages, where glycolysis‐derived lactate was linked to H3K18 lactylation and induction of reparative gene expression (Wei et al. 2023; Zhang et al. 2019). The enzymatic framework has since expanded, with evidence for distinct lactyl‐CoA‐generating activities, lactyltransferase mechanisms and Class I HDAC delactylases (Liu, Ren, et al. 2025; Moreno‐Yruela et al. 2022; Zhu et al. 2025). Much of this mechanistic work comes from non‐CNS systems, so it establishes biochemical precedent rather than proving an equivalent pathway in AD brain cells.

Metabolic regulation can occur upstream of lactate itself. BCAP‐dependent metabolic reprogramming promotes aerobic glycolysis and lactate production and thereby supports histone lactylation during the transition of macrophages toward a reparative state (Irizarry‐Caro et al. 2020). The relevant conclusion is that BCAP‐linked metabolic flux can tune histone lactylation and macrophage state. Lactylation also occurs on non‐histone proteins, and its consequences differ from those of histone lactylation. In pro‐inflammatory macrophages, lactylation of PKM2 at K62 stabilizes the tetrameric form of the enzyme, increases pyruvate kinase activity, and reduces its nuclear distribution, thereby restraining inflammatory metabolic adaptation and favoring a reparative phenotype (Wang, Yang, et al. 2022). PKM2 lactylation should therefore be assessed separately from histone lactylation.

Microglial studies illustrate why residue and context matter. In AD‐associated microglia, H4K12 lactylation is enriched at glycolytic gene promoters and participates in a positive‐feedback circuit involving PKM2 and glucose metabolism (Pan et al. 2022). In senescent microglia, H3K18 lactylation is associated with NF‐κB activation and increased expression of SASP factors such as IL‐6 and IL‐8 (Wei et al. 2023). The different residues, cell states, and metabolic settings preclude treating these findings as one generalized lactate‐driven inflammatory pathway.

Other CNS models reinforce this context dependence. H4K12 lactylation can promote reparative responses after spinal cord injury, H3K18 lactylation can support neuroprotection after ischaemic stroke, and H3K9 lactylation has been linked to inflammatory programmes in cerebral ischaemia and oxygen–glucose deprivation (He et al. 2024; Hu et al. 2024; Li, Wang, et al. 2025; Si et al. 2024; Wang, Zhou, et al. 2025). A Parkinson disease model likewise connected enhanced glycolysis‐derived lactate to microglial activation through histone lactylation (Qin et al. 2025). These studies establish site‐specific biochemical precedent but cannot substitute for direct validation in human AD tissue.

A central causal challenge is to distinguish chromatin‐mediated effects from the broader consequences of changing glycolysis or lactate. Such interventions simultaneously alter ATP production, redox state, mitochondrial function, and signaling. A convincing lactylation mechanism would therefore need to connect metabolic flux to the relevant nuclear substrate pool, identify the specific histone site and genomic targets, and show that perturbing the site or its writer/eraser changes the disease‐relevant phenotype.

### 5.3 NAD+, Redox Metabolism and Sirtuin‐Dependent Histone Regulation

NAD+ connects metabolism to chromatin through sirtuin‐dependent deacetylation and through mitochondrial and DNA‐damage pathways. During senescence and aging, reduced SIRT1 activity is associated with increased acetylation at selected inflammatory loci and enhanced SASP expression (Hayakawa et al. 2015; Xu et al. 2020). In microglia, SIRT1 can also deacetylate RelA/p65 and limit NF‐κB‐dependent inflammatory responses and amyloid‐β‐associated toxicity (Chen et al. 2005). These actions include non‐histone targets, so a change in SIRT1 activity should not automatically be interpreted as a histone‐specific mechanism.

Brain NAD+ homeostasis is disrupted in human AD, and pharmacological restoration of NAD+ homeostasis can reverse multiple disease‐associated phenotypes in advanced mouse AD models (Chaubey et al. 2026). These findings support the disease relevance of NAD+ metabolism but do not establish histone acetylation as the dominant mediator. NAD+ restoration can affect sirtuins, mitochondrial function, oxidative stress, and DNA‐damage responses in parallel. The mechanistic question is therefore whether a defined NAD+‐sirtuin‐histone circuit contributes measurably to the therapeutic phenotype in the relevant CNS cell type.

Redox state provides a complementary connection between metabolism and chromatin. Reactive oxygen species and antioxidant capacity can alter chromatin‐regulatory enzymes while also contributing to DNA damage (Balamurli et al. 2024; Rahman et al. 2025). The same oxidative shift can therefore produce transcriptional effects through chromatin enzymes, DNA lesions, inflammatory signaling, or mitochondrial dysfunction. Disentangling these routes is essential before assigning a specific histone PTM a causal role.

### 5.4 S‐Adenosylmethionine and α‐Ketoglutarate‐Dependent Histone Methylation

Histone methylation is controlled by opposing methyltransferase and demethylase activities. S‐adenosylmethionine supplies methyl groups for histone methyltransferases, whereas α‐ketoglutarate supports JmjC‐domain histone demethylases (Akakura and Tabibzadeh 2026; Malone et al. 2026). Metabolic perturbations can therefore influence methylation from both sides of the deposition‐removal balance. As with acetyl‐CoA, however, a global metabolite measurement does not establish a global histone outcome.

Mitochondrial stress provides a relevant example. In human microglial models, activation of the mitochondrial unfolded protein response is associated with metabolic rewiring, S‐adenosylmethionine depletion and a senescent phenotype with altered inflammatory communication (Perez J et al. 2026). This observation links mitochondrial proteotoxic stress to methyl‐donor availability in a human microglial system, but it does not by itself identify which histone methylation sites change or whether those changes are required for the senescent phenotype.

Alpha‐ketoglutarate provides a complementary route through JmjC demethylases and TET enzymes. Experimental senescence models in which the α‐ketoglutarate‐TET axis is perturbed show altered chromatin states and reduced senescence plasticity, with reversal after restoration of the relevant metabolic pathway (Akakura and Tabibzadeh 2026). Because α‐ketoglutarate acts on both histone and DNA demethylation systems, effects attributed to “epigenetic regulation” should not automatically be assigned to histone methylation alone.

The outstanding problem is therefore specificity. The field needs measurements that distinguish methyl‐donor depletion from altered enzyme activity, define which methyltransferases or demethylases are rate limiting, and determine whether the resulting histone marks alter defined enhancers, promoters, or heterochromatic domains in AD‐relevant cells.

### 5.5 Other Acyl Donors: Crotonyl‐CoA, Succinyl‐CoA, Malonyl‐CoA and Ketone Bodies

Intermediary metabolism supplies a broader repertoire of acyl donors beyond acetyl‐CoA and lactate. Crotonyl‐CoA supports p300‐dependent histone crotonylation and transcriptional activation (Sabari et al. 2015). Succinyl‐CoA supports histone succinylation, and KAT2A coupled to the α‐ketoglutarate dehydrogenase complex catalyzes H3K79 succinylation; H3K122 succinylation has also been linked to nucleosome destabilization (Wang et al. 2017; Zhang et al. 2011; Zorro Shahidian et al. 2021). These findings establish a direct biochemical route from TCA‐cycle metabolism to histone acylation, but the neuroinflammatory consequences of these marks remain much less defined than those of acetylation or lactylation.

Histone malonylation provides a further potential connection to lipid metabolism. Malonyl‐CoA is generated by acetyl‐CoA carboxylase 1, while SIRT5 and KAT2A have been implicated in the regulation of histone malonylation (Peng et al. 2011; Zhang et al. 2023). Evidence for histone malonylation in neuroinflammation remains sparse. Non‐histone malonylation, such as GAPDH K213 malonylation in inflammatory signaling, should therefore be regarded as biochemical context rather than as direct evidence for a histone mechanism (Galván‐Peña et al. 2019).

Ketone metabolism illustrates a related but distinct route. Beta‐hydroxybutyrate can inhibit Class I histone deacetylases and can also serve as a substrate for histone lysine β‐hydroxybutyrylation (Shimazu et al. 2013; Xie et al. 2016). These mechanisms should not be conflated: changing deacetylase activity alters pre‐existing acetylation states, whereas installing β‐hydroxybutyrylation creates a different chemical signal with distinct reader and locus requirements.

### 5.6 From Metabolic Association to Causal Chromatin Regulation

Metabolic‐chromatin coupling provides a plausible mechanism through which persistent metabolic stress may stabilize cellular states, but metabolic association alone does not establish causality. A rigorous causal chain should connect four levels: a defined metabolic flux or substrate pool; the relevant writer, eraser or reader; a specific histone PTM at disease‐relevant loci; and a downstream transcriptional or cellular phenotype. The strongest evidence should also demonstrate perturbation and, where the model permits, persistence after the initiating stimulus is removed.

This standard is particularly important in AD. Changes in glycolysis, lactate, NAD+, oxidative stress and TCA‐cycle intermediates can alter ATP production, redox balance, protein modification and signaling independently of chromatin. Microglia, astrocytes and neurons also differ in metabolic flux, nuclear substrate availability and chromatin enzyme expression. A metabolic‐PTM pathway established in macrophages, fibroblasts, cancer cells or non‐AD injury models is therefore best described as mechanistic precedent until it is validated in the appropriate CNS population.

The most informative experiments will combine isotope tracing or compartment‐resolved metabolomics with locus‐resolved histone profiling and targeted perturbation of writers, erasers, or substrate‐generating pathways. Cell‐type‐specific genetic manipulation and single‐cell or spatial readouts will be particularly valuable because bulk tissue measurements cannot resolve whether a histone change reflects altered cell composition or an intracellular state transition. Finally, withdrawal and longitudinal experiments are needed to determine whether an altered metabolic‐histone circuit persists beyond continuous disease exposure. This progression from association to flux, locus, perturbation, and persistence provides a practical framework for testing whether metabolism merely accompanies AD chromatin remodeling or helps maintain it.

## 6 Histone Post‐Translational Modifications in AD Pathology: Proteinopathy–Chromatin–Neuroimmune Feedback

Aβ plaques and tau neurofibrillary tangles are conventionally positioned upstream of the molecular and cellular changes that accompany AD. Increasing evidence instead supports a bidirectional relationship between proteinopathy and chromatin: Aβ and tau can reshape histone modifications and chromatin organization, whereas chromatin‐dependent transcription can influence amyloidogenic processing, neuronal resilience, and inflammatory competence.

### 6.1 Amyloid‐β: Bidirectional Coupling Between Amyloidogenic Processing and Chromatin

Aβ‐related pathology can be coupled to chromatin‐dependent regulation of amyloidogenic processing. In N2a cells expressing Swedish mutant APP, p300 binds the PS1 and BACE1 promoters and increases local H3 acetylation; pharmacological inhibition of p300 reduces promoter acetylation together with PS1 and BACE1 expression (Lu et al. 2014). More recent work in APP/PS1 mice showed that the histone deacetylase inhibitors valproic acid and WT161 reduce BACE1 and PSEN1 expression, increase ADAM10, and attenuate pathological and cognitive phenotypes (Zhang, Wang, Ye, et al. 2024). HDAC3 has also been shown to occupy the APP promoter, and its inhibition induces transcriptional programmes associated with neuroregeneration in AD models (Davis et al. 2024). Together, these studies support a role for histone acetylation in regulating APP processing, but pharmacological HDAC inhibition acts on multiple substrates and therefore does not establish any single histone residue as the sole mediator.

The relationship is not uniformly pro‐amyloidogenic. In induced neurons derived from individuals carrying an APP duplication, lowering CBP and p300 decreases H3K27ac and suppresses a set of homeostatic amyloid‐reducing genes, resulting in increased extracellular Aβ1–42 (Xu et al. 2024). Thus, an increase in H3K27ac detected in AD tissue cannot be interpreted as uniformly pathogenic; at some loci, acetylation may sustain compensatory or protective transcriptional programmes. Soluble Aβ can itself perturb neuronal chromatin. Across neural cell models, primary neurons, Tg2576 mice and human AD neocortex, Aβ exposure is associated with changes in H3K14ac, H3S10 phosphorylation and H3K9 dimethylation, indicating that amyloid stress can affect multiple layers of the histone modification landscape (Lithner et al. 2013). Whether these changes are adaptive, pathogenic or context‐dependent remains unresolved.

A second route connects Aβ pathology to metabolic chromatin signaling. In a 2024 ad study, reduction of the mitochondrial tricarboxylic‐acid‐cycle enzyme IDH3β increased lactate, histone lactylation and PAX6 expression, establishing a positive‐feedback loop in which PAX6 further suppressed IDH3β and was associated with tau hyperphosphorylation, synaptic impairment and cognitive deficits (Xin Wang et al. 2024). This study does not concern a single histone site as an isolated Aβ effector, but it provides a mechanistic example of how altered metabolism can become coupled to chromatin‐dependent transcription within AD pathology.

Microglia provide a more direct pathology‐associated example of this metabolic–chromatin interface. In 5xFAD mice and human AD brain, H4K12 lactylation is increased in plaque‐adjacent microglia and enriched at promoters of glycolytic genes; interference with the lactate–H4K12la–PKM2 circuit attenuates microglial activation and AD‐associated phenotypes (Pan et al. 2022). A distinct H3K18 lactylation programme has been linked to senescent microglia, NF‐κB activity and production of IL‐6 and IL‐8 (Wei et al. 2023). More recently, H4K12la was shown to promote NLRP3 transcription in a cigarette‐smoke‐accelerated AD model, coupling elevated lactate to microglial autophagy and inflammatory activation (Wang, Xia, et al. 2025). These studies should not be collapsed into a single “histone lactylation programme”: the residues, initiating stimuli and causal interventions differ. Their convergence instead suggests that metabolic changes generated by or superimposed on proteinopathy can alter the transcriptional threshold of responding microglia.

### 6.2 Tau: Distributed Chromatin Remodeling and Loss of Transcriptional Constraint

Tau is associated with a broader chromatin footprint than can be captured by any single promoter. In 669 aged human prefrontal cortex samples, tau burden was associated with altered H3K9ac across 5990 of 26,384 chromatin domains, whereas genome‐wide association with Aβ was substantially weaker (Klein et al. 2019). The tau‐associated changes occurred in large genomic domains linked to three‐dimensional chromatin organization and nuclear‐lamina association, and analogous patterns were detected in mouse tau models and tau‐expressing induced neurons (Klein et al. 2019). These data establish a widespread association between tau burden and histone acetylation, but they do not by themselves establish causality.

Targeted perturbations provide more specific examples in which chromatin repression constrains tau‐related pathology. BMI1, a component of Polycomb repressive complexes, is reduced in sporadic AD brain and in AD patient‐derived neurons; BMI1 deficiency is accompanied by reduced H2A monoubiquitination, increased MAPT expression and activation of GSK3β‐ and p53‐associated pathways, together with Aβ secretion, tau phosphorylation and neurodegenerative phenotypes (Flamier et al. 2018). The mechanistic inference is therefore that BMI1‐dependent chromatin repression is protective in this model; H2A ubiquitination should not be regarded as the sole downstream effector. The long non‐coding RNA NEAT1 provides a complementary route from RNA regulation to tau‐associated chromatin control: in SH‐SY5Y cells and APP/PS1 mice, NEAT1 recruits p300 to the FZD3 promoter and maintains H3K27ac, whereas NEAT1 loss reduces FZD3 expression, alters Wnt signaling and increases tau phosphorylation (Zhao et al. 2020).

Human AD tissue further argues against a uniform histone signature. Regional changes in H4K16ac were reported in aging and AD brain, whereas integrated multi‐omics identified disease‐associated gains in H3K27ac and H3K9ac, and acetylome‐wide profiling of entorhinal cortex identified 4162 ad‐associated H3K27ac peaks (Marzi et al. 2018; Nativio et al. 2018, 2020). Recent work in tauopathy adds another layer: tauopathic hippocampal neurons show histone hyperacetylation at neuronal enhancers linked to glutamatergic regulation, together with dysregulated cholesterol biosynthesis; correction with the epi‐drug CSP‐TTK21 counteracts enhancer hyperacetylation and improves object memory (Paiva et al. 2024). A complementary 2025 study found that tau reshapes the transcriptional landscape toward intermediate AD stages and is accompanied by progressive loss of heterochromatin‐associated features in cellular and human AD material (Siano et al. 2025). Collectively, these findings favor a model of region‐, locus‐ and disease‐stage‐specific chromatin remodeling rather than a single global “AD histone state”.

Astrocytic chromatin regulation provides a further route by which tau pathology can be amplified outside neurons. HDAC7 is increased in astrocytes from AD patients and PS19 tau mice; astrocyte‐specific genetic or pharmacological inhibition of HDAC7 enhances tau uptake and lysosomal degradation by preserving acetylation of TFEB at K310, restoring its nuclear translocation and lysosomal biogenesis, and improving tau pathology and cognitive performance (Ye et al. 2025). This study is important because it links a defined chromatin enzyme to a cell‐type‐specific protein‐clearance programme and, in turn, to tau burden.

Histone methylation is also emerging as a functionally relevant regulator of AD‐associated proteopathy. A brain‐penetrant G9a inhibitor, MS1262, improved memory and noncognitive phenotypes in multiple AD mouse models and reversed expression or phosphorylation of several AD‐associated proteins (Xie et al. 2025). A second study of the selective G9a inhibitor FLAV‐27 reported reduced Aβ and phosphorylated tau aggregation, improved neuritic complexity and cognitive or synaptic phenotypes in both 5xFAD and SAMP8 models, accompanied by multi‐omic remodeling of H3K9me2/H3K18me‐associated repression (Bellver‐Sanchis et al. 2026). These pharmacological studies strengthen the case for G9a‐dependent repressive chromatin as a disease‐relevant node, but because G9a has multiple molecular outputs, they should not be interpreted as proof that H3K9me2 or H3K18me2 at a particular locus is itself the singular driver of proteopathy.

### 6.3 Convergence on Chromatin States That Control Inflammatory Competence

Once proteinopathy has altered neuronal and glial states, chromatin‐regulatory systems can determine whether the ensuing immune response remains transient or becomes self‐reinforcing. Repressive methylation provides one example. In an AD mouse model, reduced JMJD3 was associated with increased H3K27me3, lower BDNF expression and exacerbated mitochondrial‐stress and Aβ‐related phenotypes; increasing JMJD3 or BDNF partially reversed these abnormalities (Wang, Zhang, et al. 2022). A related mechanistic precedent from Parkinson disease showed that microglial Jmjd3 is required for H3K27me3 removal during a beneficial microglial state transition (Tang et al. 2014). Together, these studies support a state‐ and context‐dependent role for the JMJD3–H3K27me3 axis rather than a universal inflammatory interpretation of the mark.

Acetylation offers more locus‐resolved examples in AD. In early AD brain and an hAPP knock‐in model, reduced FZD1 and FZD7 expression accompanies loss of promoter‐associated H4K16ac and increased recruitment of nuclear SIRT2; SIRT2 inhibition restores H4K16ac and FZD1/FZD7 expression, linking aberrant deacetylation to impaired Wnt signaling (Palomer et al. 2022). Complementary work in APP/PS1 mice showed that selective Class I HDAC inhibition reduces microglial inflammatory changes in the entorhinal cortex (Wang et al. 2023). Because the latter intervention targets an enzyme class rather than a single histone residue, it supports the tractability of chromatin regulation without assigning the phenotype to one PTM.

Metabolic acylation and innate immune signaling provide another convergence. H4K12la at plaque‐adjacent microglial glycolytic promoters participates in a lactate–PKM2 feedback loop, while H3K18la is associated with inflammatory activation in senescent microglia and H4K12la can promote NLRP3 transcription in a stress‐accelerated AD model (Pan et al. 2022; Wang, Xia, et al. 2025; Wei et al. 2023). In 2026, increased microglial H3K18 crotonylation was linked experimentally to STAT1 expression and cognitive dysfunction in both 5xFAD and Aβ42‐injection models; elevating crotonylation increased microgliosis and inflammatory cytokines, whereas STAT1 inhibition attenuated these effects (Weng et al. 2026). The distinction between residues is mechanistically important: “histone acylation” is not itself the regulatory unit, and the direction of effect depends on the modified residue, cell state, genomic context and initiating metabolic signal.

A particularly direct link between histone acetylation and innate immunity was reported in 2026. KAT7 and its associated H3K14ac mark were elevated in microglia from 5xFAD mice and human AD brains. Integrative epigenomic and transcriptomic analyses identified Cmpk2 as a KAT7‐responsive target; loss of KAT7 reduced Cmpk2 transcription, impaired mitochondrial DNA replication and cytosolic mtDNA release, and consequently suppressed cGAS–STING and NLRP3 signaling. Microglia‐specific Kat7 deletion or pharmacological inhibition reduced neuroinflammation and Aβ burden and improved synaptic plasticity and cognition in 5xFAD mice (Liu et al. 2026). This study provides a much more direct mechanistic bridge between a defined histone acetylation programme and microglial mitochondrial innate immunity than is available from pathway‐level cGAS–STING studies alone.

Another 2026 study identified the histone demethylase PHF2 as an AD‐associated regulator of inflammatory genes. PHF2 was increased in AD postmortem tissue, AD patient‐derived iPSC neurons and 5xFAD brain; bidirectional manipulation coupled with ChIP‐seq and transcriptional analyses linked PHF2 to inflammatory and neurodegeneration‐related genes including Stat3, Nfkbia, Nfkb2, Tnfrsf1a, IL6st, Notch2 and Csf1. Phf2 knockdown in 5xFAD reduced microglial and astrocytic activation and restored glutamatergic synaptic function (Yang, Ren, et al. 2026). The study places chromatin enzyme activity upstream of a distributed inflammatory transcriptional programme, although it does not imply that all PHF2‐responsive genes are regulated through one histone site.

Genome instability provides a second route into innate immune activation. Loss of BRAP increases H2A mono‐ and poly‐ubiquitination, persistent DNA damage, proteostasis defects, senescence and neuroinflammation, and increased H2A ubiquitination has been detected in human AD brain tissue (Guo et al. 2022). Separately, microglia‐specific cGAS deletion and STING blockade attenuate Aβ‐associated pathology and inflammatory signaling in experimental AD models (Chung et al. 2024; He et al. 2025). A 2025 PNAS study further showed that excessive Aβ engulfment by microglia causes mitochondrial stress and cytosolic mtDNA release, activating cGAS–STING; the resulting inflammatory response impairs further Aβ clearance, creating an Aβ–STING negative‐feedback loop that can be interrupted experimentally (Tian et al. 2025). These studies establish the biological relevance of the cGAS–STING branch but, before the KAT7 work, did not identify a defined histone lesion upstream of the mitochondrial DNA signal. The newer KAT7 study therefore closes an important mechanistic gap, while the precise contribution of chromatin damage itself remains context‐dependent.

Finally, recent epigenomic studies argue that inflammatory chromatin states are spatially and genetically heterogeneous. In a mouse AD model, plaque‐associated and non‐plaque‐associated microglia displayed distinct chromatin accessibility states, with non‐plaque microglia representing a dynamic population that can transition toward plaque‐associated states (Ardura‐Fabregat et al. 2025). In human microglia xenografted into APPNL‐G‐F brains, APOE isoforms produced widespread genotype‐dependent differences in both transcription and chromatin accessibility, indicating that disease‐associated microglial state is shaped by genotype as well as local pathology (Murphy, Hu, et al. 2025). These observations provide an important framework for interpreting histone PTMs: the same residue cannot be assumed to have the same transcriptional consequence in every microglial population or anatomical niche.

### 6.4 From Feedback Loops to Testable Models of Persistent Disease

Taken together, the evidence supports a nested feedback architecture rather than a linear “proteinopathy → inflammation” pathway. Aβ‐related stress can alter chromatin‐dependent regulation of APP processing and neuronal transcription; tau can impose broader and spatially organized chromatin changes; and proteinopathic signals can reshape the metabolic and inflammatory states of glial cells. Histone PTMs then influence the transcriptional thresholds that govern amyloid processing, proteostasis, synaptic signaling, cellular resilience, and inflammatory competence, thereby feeding back on the tissue environment in which Aβ and tau accumulate. The relevant regulatory unit is therefore not an isolated histone mark but a chromatin state embedded within a cellular and metabolic state.

The strength of evidence across this network is uneven. The most direct AD evidence now comes from locus‐ or cell‐resolved studies of p300‐dependent APP processing, CBP/p300‐dependent amyloid‐reducing programmes, SIRT2–H4K16ac control of Wnt receptors, H4K12la‐associated microglial metabolic feedback, H3K18cr–STAT1 signaling, HDAC7–TFEB control of astrocytic tau clearance and KAT7–H3K14ac control of mitochondrial innate immunity (Liu et al. 2026; Lu et al. 2014; Weng et al. 2026; Xu et al. 2024; Ye et al. 2025). Human epigenomic studies establish reproducible changes in H4K16ac, H3K27ac and H3K9ac and demonstrate that tau‐associated chromatin remodeling is extensive, but these association studies do not by themselves establish causality (Klein et al. 2019; Marzi et al. 2018; Nativio et al. 2018, 2020; Pan et al. 2022). Pharmacological studies of G9a, HDACs and other chromatin enzymes provide convergent functional evidence but necessarily require caution because these regulators have multiple genomic and non‐genomic substrates (Bellver‐Sanchis et al. 2026; Wang et al. 2023; Xie et al. 2025; Zhang, Wang, Ye, et al. 2024).

Recent studies also broaden the metabolic–epigenetic interface beyond lactylation. In 3xTg‐AD mice and human AD tissue, β‐hydroxybutyrate and H3K9 β‐hydroxybutyrylation are reduced; restoring HMGCS2 or replenishing β‐hydroxybutyrate increases H3K9bhb at promoters of NMDA receptor subunits, Syn1, and axon‐related genes and improves synaptic plasticity and cognition (Yu et al. 2026). This finding reinforces a central principle of this section: metabolic intermediates can act as chromatin substrates, but their functional consequences remain locus‐ and cell‐state‐specific.

The central unresolved question is whether any AD‐associated histone modification functions as a persistent memory variable rather than as a contemporaneous readout of disease stress. Demonstrating such a role requires more than detecting a changed mark. It requires cell‐type‐ and locus‐resolved measurements; perturbation of the relevant writer or eraser; evidence that the modification changes a defined transcriptional programme; and demonstration that the altered state persists, or is reproducibly re‐established, after the initiating proteinopathic stimulus is removed. Longitudinal and spatial profiling combined with metabolic tracing, cell‐type‐specific perturbation and residue‐resolved genetic manipulation could therefore move the field from cataloging AD‐associated chromatin changes toward identifying which chromatin states actually store, amplify or propagate neurodegenerative stress.

## 7 Therapeutic Potential and Limitations

Interventions targeting histone PTMs during AD pathological progression are moving from concept to therapeutic testing. Given the chronic nature of AD pathological states, their cell‐type specificity, and the progressive decline in reversibility with disease progression, simply blocking cytokines or clearing protein aggregates is often insufficient to dismantle established pathological circuits (Amniouel et al. 2025; Li, Qiu, et al. 2025; Olawade et al. 2025) By contrast, resetting pathological chromatin toward homeostasis offers a complementary strategy. This chapter discusses five classes of interventions—small‐molecule epigenetic modulators, metabolic–epigenetic interventions, natural products, CRISPR‐based epigenome editing, and lifestyle and dietary approaches—and evaluates the therapeutic potential and limitations of each (Figure 5; Table 4).

**FIGURE 5:**
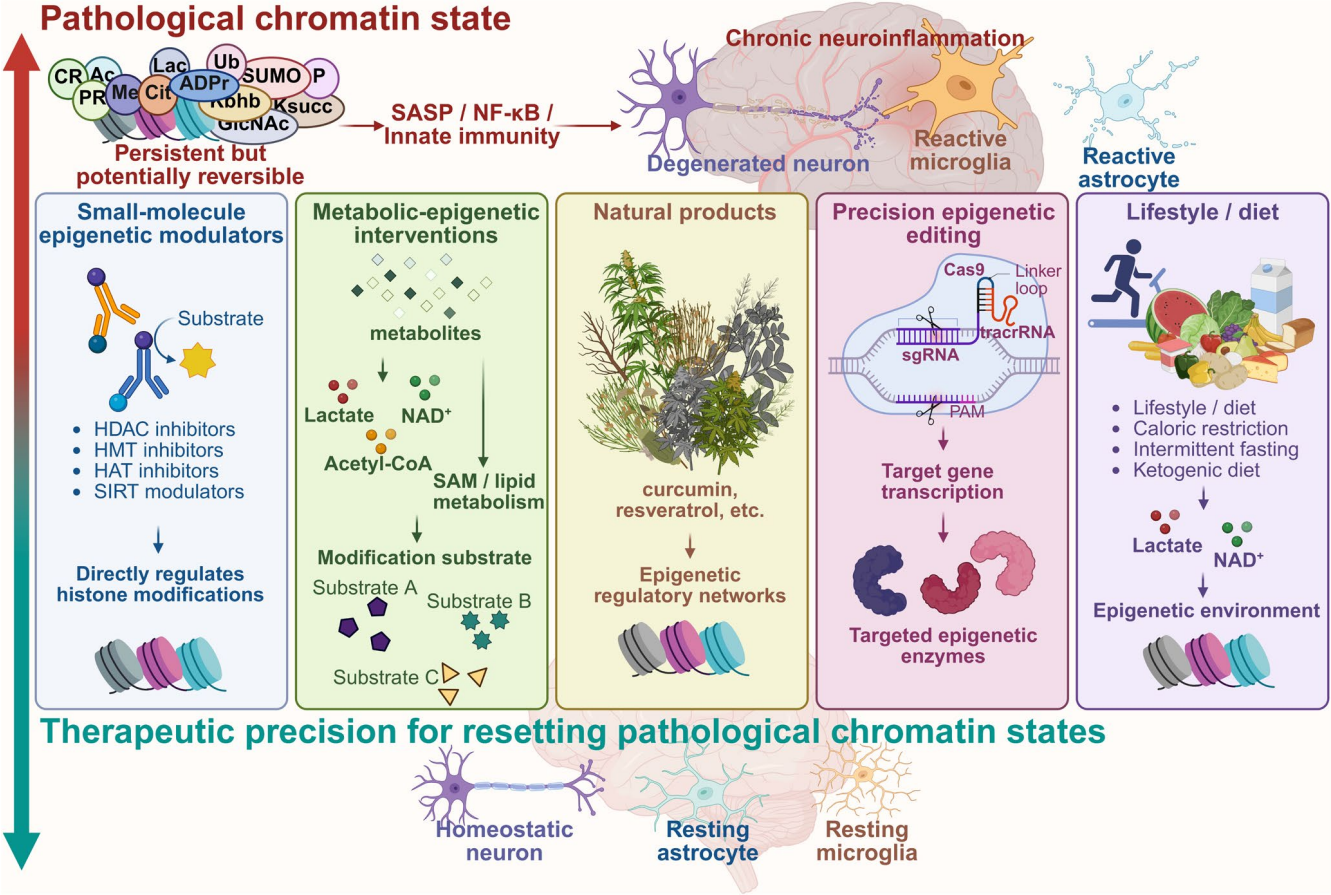
Therapeutic for resetting pathological chromatin states. This schematic depicts a translational framework in which multi‐tiered therapeutic interventions converge to reverse a persistent, yet potentially reversible, pathological chromatin state and restore brain homeostasis. The pathological state is characterized by nucleosomes bearing aberrant combinatorial histone modifications that drive inflammatory signaling pathways, ultimately leading to chronic neuroinflammation accompanied by degenerated neurons, reactive microglia, and reactive astrocytes. Five distinct therapeutic strategies are highlighted to reset these aberrant epigenetic landscapes, including small‐molecule epigenetic modulators, metabolic‐epigenetic interventions, natural products, precision epigenetic editing using CRISPR/Cas9, as well as lifestyle and dietary interventions. Ultimately, these precision strategies aim to restore tissue homeostasis—characterized by resting microglia, resting astrocytes, and healthy neurons—by ameliorating aberrant histone post‐translational modifications. This figure was created in BioRender.com.

**TABLE 4:** Epigenetic therapeutics evaluated in preclinical and clinical studies for neurodegenerative diseases.

Strategy class	Agent/intervention	Primary target	Disease and model	Epigenetic change	Key outcome	Translational stage	References
Small‐molecule epigenetic modulators	Simvastatin	HDAC2 (indirect)	AD (Aβ₁₋₄_2_ ICV mouse)	↑ H4K5ac at Bdnf promoter	↑ cognition, ↑ BDNF, ↑ neurogenesis, ↑ LTP	Preclinical	Cai et al. (2026)
	AGK2 (or other SIRT2 inhibitor)	SIRT2	AD (human early hippocampus; AD mice)	Restores H4K16ac at Fzd1/Fzd7 promoters	↑ Fzd1/Fzd7 expression, restores Wnt signaling, potential synapse protection	Preclinical	Palomer et al. (2022)
	BG45	Class I HDAC (HDAC3 selective)	AD (APP/PS1 mice, entorhinal cortex)	↑ H3K9K14ac, ↑ H3ac; ↓ HDAC1/2/3 expression	↓ microgliosis/astrogliosis, ↓ Aβ deposition, ↓ p‐tau, ↓ IL‐1β/TNF‐α, ↑ synaptic proteins, ↑ CREB/BDNF pathway	Preclinical	Wang et al. (2023)
	FLAV‐27	G9a (EHMT2)	AD ( *C. elegans* ; SAMP8 mouse; 5xFAD mouse)	↓ H3K9me2, ↓ H3K18me‐mediated repression; global H3K9me2 reprogramming	↓ Aβ and p‐tau aggregation, ↑ neuritic complexity (in vitro); ↑ mobility, ↑ lifespan, ↑ mitochondrial respiration ( *C. elegans* ); ↑ memory, ↑ social behavior, ↑ synaptic structure, ↓ ferroptosis vulnerability, normalizes plasma/brain biomarkers (SMOC1, H3K9me2, p‐Tau181)	Preclinical (first‐in‐class, in vivo efficacy)	Bellver‐Sanchis et al. (2026)
	GSK126	EZH2	AD (Aβ₁₋₄_2_‐induced rat model)	↓ H3K27me3 at SOD1 promoter	↑ SOD1, ↓ ROS, ↑ cognition	Preclinical	Zhong et al. (2026)
	BCI‐121	Smyd3 (H3K4me3 writer)	Tauopathy (P301S Tau mice; AD human PFC)	↓ H3K4me3 (at Fbxo2 gene)	↓ Fbxo2 expression → ↑ NMDAR function, ↑ cognition, restores synaptic deficits	Preclinical	Williams et al. (2023)
	WDR5‐0103	WDR5‐MLL interaction (H3K4me3 writer)	AD (human PFC postmortem; AD mouse models)	↓ H3K4me3 at promoters of upregulated genes (e.g., Sgk1)	↓ tau hyperphosphorylation, ↑ glutamatergic synaptic function, ↑ memory, ↓ Sgk1 expression	Preclinical	Cao et al. (2020)
	UNC0642	G9a/GLP (H3K9me2 writer)	AD (5XFAD mouse)	↓ H3K9me2, ↓ 5‐mC, ↑ 5‐hmC	↑ cognition, ↓ ROS, ↓ IL‐6/TNF‐α, ↓ GFAP, ↑ NRF2/Hmox1, ↑ BDNF/NGF/SYN, ↓ Aβ plaques	Preclinical	Griñán‐Ferré et al. (2019)
	GSK‐J4	KDM6A/B (H3K27me3 demethylase), KDM5B/C (H3K4me3 demethylase)	PD (6‐OHDA rat; cell models)	↑ H3K4me3 (via KDM5 inhibition)	↓ labile iron, ↑ ferroportin‐1, ↓ oxidative stress, rescues dopaminergic neurons, improves motor function	Preclinical	Mu et al. (2020)
Metabolic–epigenetic interventions	NR/NMN	NAD^+^ metabolism; SIRT3/SIRT6; PARP1; mitochondrial function	AD (3xTgAD/Polβ^+^/^−^ mice; older adults with MCI)	↑SIRT3/6, ↓SOD2, ↓acetylation, PARylation; ↑global DNA methylation in PBMCs	Increased brain NAD^+^/NADH ratio, reduced pTau, neuroinflammation, DNA damage, and apoptosis, improved cognition and hippocampal LTP, without affecting Aβ deposition	Phase II clinical	Hou et al. (2018), Orr et al. (2024)
	PKM2 inhibition (shikonin, compound 3 K; Pkm2 knockdown/microglia‐specific knockout)	PKM2; glycolysis/H4K12la/PKM2 positive feedback loop	AD (5XFAD mice; isolated microglia; BV2 cells; AD patient brain tissue)	↓H4K12la levels; reduced H4K12la enrichment at Hif‐1α, Pkm, and Ldha promoters	↓lactate, Pan Kla, and H4K12la; ↓expression of glycolytic genes; attenuated microglial activation and neuroinflammation	Preclinical	Pan et al. (2022)
	Tributyrin (oral butyrate prodrug)	Gut microbiome → butyrate → HDAC inhibition	AD (3 × Tg‐AD mice)	↑ H3K9ac, ↑ H3K14ac in hippocampal neurons	Restores H3K9/K14ac; attenuates tau hyperphosphorylation, oxidative stress, memory deficits, neuromuscular dysfunction	Preclinical	Chilton et al. (2024)
Natural products	PPV‐6 (polysaccharides from *Basella alba* )	SHH (indirect, via downregulation)	AD (Aβ_2_₅₃₅–treated rat cortical neurons)	↓ p‐Histone H3 (Ser10) as marker of cell cycle re‐entry	↓ neuronal apoptosis, ↓ cell cycle markers (cyclin D1, PCNA), ↑ viability, protects mitochondrial function	Preclinical	Hou et al. (2024)
	Curcumin	Multiple (HDAC, DNMT, HAT, JMJD3)	AD (various models)	↑ H3K27ac, ↓ DNA methylation, ↓ H3K27me3 (via JMJD3 upregulation)	↓ Aβ, ↓ inflammation, ↑ BDNF, improved MSR, anti‐apoptosis	Preclinical	Li, Wang, et al. (2021), Wang, Zhang, et al. (2022)
	Hydroxytyrosol	HDAC6 (indirectly down‐regulated by miRNA‐124)	AD (oAβ₄_2_‐induced mouse model; organotypic hippocampal slice culture)	↑miRNA‐124, ↓HDAC6	Improves impulsive decision‐making, alleviates anxiety‐like behaviors, transcriptional activation of CRTC1	Phase II clinical	Fabiani et al. (2021)
	Resveratrol	SIRT1 activation; Aβ, Tau, NF‐κB, PI3K/Akt/GSK3β, Wnt, TLR4/NF‐κB/STAT	AD (SH‐SY5Y; N2a, PC12; BV2; primary microglia;3xTg‐AD; APP/PS1; Tg6799; STZ rats; patients with mild‐to‐moderate AD)	↑SIRT1 activation (NAD^+^‐dependent histone deacetylase); ↓HDAC activity	Reduces Aβ aggregation and plaque burden, decreases Tau phosphorylation, exerts anti‐inflammatory and antioxidant effects, improves synaptic and cognitive function, enhances autophagy and mitochondrial function; a Phase II trial showed safety and BBB penetration, with reduced CSF Aβ40 but greater brain volume loss than placebo	Phase II clinical	Puranik et al. (2025)
	Microglial SR knockout (conditional, Lyz2‐Cre)	Serine racemase (SR)	AD (5 × FAD mice, male‐specific)	↑ H3K18la in plaque‐associated microglia	↑ anti‐inflammatory factors, ↑ phagocytosis, ↓ Aβ plaques, ↑ spatial memory (male); sex‐specific effect	Preclinical	Zhou et al. (2026)
Precision epigenome editing	dCas9‐mediated Rnf20 upregulation (CRISPR activator)	Rnf20 (H2B ubiquitin E3 ligase)	Aged hippocampus (male rats, age‐related memory decline model)	↑ H2B monoubiquitination (H2Bubi)	↑ memory retention (contextual fear conditioning), alters transcriptome (51 learning‐related genes)	Preclinical	Kincaid et al. (2025)
	KMT2B knockdown (siRNA/shRNA)	KMT2B (H3K4me3 writer)	HHcy (rat model; N2a cells; primary hippocampal neurons)	↓ H3K4me3 → restoration of H3K14ac (via ANP32A downregulation)	Restores histone acetylation, synapse‐associated protein expression, and synaptic plasticity	Preclinical	Chai et al. (2024)
	CBP/p300 knockdown (siRNA/shRNA)	CBP/p300 (H3K27ac writers)	AD (iPSC‐neurons from APP duplication patients)	↓ H3K27ac at enhancers of homeostatic amyloid‐reducing genes	↓ expression of protective genes, ↑ secretion of Aβ(1–42), loss of compensatory program	Preclinical	Xu et al. (2024)
	Aerobic exercise (treadmill, 8 weeks)	ADRB2 → AMPKα → p300 nuclear translocation	AD (APP/PS1 mice; human NHANES cohort)	↑ H4K5ac, ↑ H4K12ac	↓ Aβ pathology, ↑ synaptic plasticity, ↑ cognition, ↑ synaptic gene (GluN1) transcription	Preclinical	Chai et al. (2026)
Lifestyle and dietary interventions	β‐OHB supplementation/HMGCS2 upregulation (e.g., via ketogenic diet)	HMGCS2 (β‐OHB synthesis), β‐OHB (substrate)	AD (3xTg‐AD mice; human AD postmortem)	↑ H3K9bhb (β‐hydroxybutyrylation) at promoters of NMDA receptor subunits, Syn1, and axon‐related genes	↑ synaptic plasticity, ↑ NMDA receptor expression, ↑ cognition, restores H3K9bhb levels	Preclinical	Yu et al. (2026)
	Dietary restriction	Nutrient‐sensing pathways (indirect)	Aging (mouse brain)	Partially restores age‐related loss of H3K27me3, H3R2me2, H3K79me3, H4K20me2; also alters H3K27ac, H4K16ac, H4R3me2, H3K56ac	Slows brain aging via epigenetic mechanisms	Preclinical	Gong et al. (2015)

### 7.1 Small‐Molecule Epigenetic Modulators

Small‐molecule epigenetic modulators remain the most extensively studied class of strategies in AD. They are currently divided into four main categories: HDAC inhibitors (Luchsinger et al. 2016), histone methyltransferase (HMT) inhibitors (Griñán‐Ferré et al. 2019), histone acetyltransferase (HAT) inhibitors (Neal and Richardson 2018), and SIRT modulators (Moussa et al. 2017). Among these, HDAC inhibitors have been studied in greater depth, whereas the other categories remain exploratory.

The cognitive improvement effects of pan‐HDAC inhibitors in AD animal models have been replicated in numerous studies, which have found restoration of marks such as H3K9ac and modulation of non‐histone substrates including NF‐κB p65 (Diniz et al. 2024; McClarty et al. 2026; Palomer et al. 2022; Wang et al. 2023). However, their clinical translation is limited by the lack of isoform selectivity, lack of cell‐type targeting, and the inability to separate histone from non‐histone effects (Joshi et al. 2025; Li, Izhar, and Kanekiyo 2025). The consequences of HDAC modulation also differ markedly among neural progenitor cells, mature neurons, and glial cells, supporting cell‐type‐aware dosing strategies (Diniz et al. 2025). Pan‐HDAC inhibition is often accompanied by higher neurotoxicity, whereas HDAC6‐selective inhibition shows a better safety window in multiple models; HDAC2 inhibition is associated with improved synaptic plasticity, HDAC3 overactivity with neuronal toxicity, and HDAC5 inhibition may impair memory consolidation (Rodrigues et al. 2020). Therefore, simply pursuing broad‐spectrum HDAC inhibition does not align with the therapeutic logic of cell‐type and chromatin‐state heterogeneity in AD, and pan‐HDAC inhibitors may best serve as pharmacological probes (Asadi et al. 2025; Li, Izhar, and Kanekiyo 2025).

Isoform‐selective inhibitors show better benefit–risk profiles in preclinical studies. The HDAC3 inhibitor RGFP966 ameliorates memory impairment in 5xFAD mice by promoting a microglial M1‐to‐M2 phenotypic switch and increasing the secretion of factors that normalize degenerating axons; its mechanism involves HDAC3 occupancy at the APP promoter and regulation of neuroregeneration‐related transcriptional programs (Davis et al. 2024). The HDAC6 inhibitors Tubastatin A and ACY‐1215 combine anti‐inflammatory activity, microtubule stabilization, and improved axonal transport in AD mouse models, restoring axonal transport function by increasing α‐tubulin acetylation (Pulya et al. 2021; Zhang et al. 2014). Multitarget design offers another optional development path: hybrid compounds combining GPR40 agonism with HDAC6 inhibitory activity can modulate tubulin acetylation, ERK phosphorylation, and microglial immune responses in cellular models (Pinheiro, de Chirico, et al. 2025).

In terms of clinical progress, the most advanced test remains the VostatAD01 study of vorinostat (SAHA) (NCT03056495). This Phase Ib trial enrolled nine AD patients before being terminated due to insufficient recruitment (Bondarev et al. 2021; Gupta et al. 2020). The reason for termination is important for interpretation: the trial provided logistical information on feasibility and safety but neither establishes inefficacy nor unacceptable toxicity. From the clinical pipeline, in addition to vorinostat, HDAC‐related agents such as EVP‐0334, RDN‐929, and CKD‐504 have entered early trials for CNS indications, with RDN‐929 targeting the HDAC‐CoREST complex and CKD‐504 being a selective HDAC6 inhibitor. However, it should be noted that the vast majority of human safety data for HDACi come from oncology trials, and the maximum tolerated dose, brain target occupancy, and long‐term safety for CNS indications remain undefined (Rodrigues et al. 2020). All of these compounds still lack cell‐type specificity, and global acetylation gains risk off‐target gene activation; progress hinges on pharmacodynamic biomarkers and patient stratification (Paniri et al. 2024; Rocha et al. 2026).

HMT inhibitors have also achieved mechanistic depth. Among them, GSK126 (EZH2), UNC0642 (G9a), and related agents have been reported to reduce H3K27me3/H3K18me/H3K9me2 and improve synaptic plasticity (Bellver‐Sanchis et al. 2026; Cao et al. 2020; Griñán‐Ferré et al. 2019; Li, Zhao, et al. 2022; Mu et al. 2020; Wang et al. 2023; Williams et al. 2023; Zhang, Wang, Zhou, et al. 2024; Zhong et al. 2026), although most studies are limited to a single model, small sample sizes, and lack BBB or long‐term safety data. For p300/CBP, two mechanistically distinct strategies must be distinguished: C646 inhibits the catalytic acetyltransferase domain, whereas CCS1477 (inobrodib) is a p300/CBP bromodomain inhibitor that blocks reader function rather than acetylation writing (Di Fede et al. 2025; Maity et al. 2021). Thus, the decrease in histone acetylation after CCS1477 treatment reflects loss of bromodomain‐mediated recruitment rather than inhibition of catalytic activity. Both show modest anti‐senescence activity in vitro but face selectivity and toxicity hurdles.

SIRT2 inhibition represents the sirtuin strategy with the most clearly defined locus‐resolved evidence in AD. The SIRT2 inhibitor AGK2 restored H4K16ac levels at the Fzd1/Fzd7 promoters, reactivated Wnt signaling, and rescued synaptic deficits in AD models, including in vivo experiments (Palomer et al. 2022). The therapeutic appeal of this pathway lies in the relative completeness of its causal chain. However, clinical development of SIRT2 inhibitors remains limited: no AD clinical trials have been conducted, and isoform‐specific risk–benefit has not been systematically evaluated. Because sirtuins depend on NAD^+^, indirect modulation of sirtuin activity via NAD^+^ precursors may offer a more physiological route of intervention, but the cell‐type‐resolved effects of this strategy remain unclear.

### 7.2 Metabolic–Epigenetic Interventions

The preceding analysis has established the close relationship between metabolism and epigenetics. The core of metabolic–epigenetic strategies lies in exploiting the direct dependence of histone modifications on metabolic substrates and cofactors (Chilton et al. 2024; Han et al. 2025; Voukia et al. 2026; Yu et al. 2026; Zhang, Yang, Li, and Zhang 2025). Specific interventions include limiting lactate production to constrain lactylation, supplementing NAD^+^ precursors (NMN, NR), and enhancing ABCA1 to restore ApoE lipidation and correct cholesterol–acetyl‐CoA coupling (AlHayani et al. 2025; Chen et al. 2023; Wang, Wang, et al. 2026). A further advantage is that lactate and NAD^+^ can be monitored in blood and CSF, and NR/NMN have demonstrated preliminary safety in mild cognitive impairment (Wu, Kupferschmid, et al. 2025; Zhang, Yang, Yu, et al. 2025), making this the clinically most proximate class.

The countervailing weakness, however, is pleiotropy: the relationship between metabolite supply and chromatin state is not a simple matter of “substrate abundance translating directly into increased modification.” Compartmentalization of nuclear metabolite pools, the efficiency of enzyme recruitment, and the rates of modification turnover jointly determine transcriptional output. One framework has proposed a “lactate signal decoding” model that integrates the lactate/pyruvate ratio, cytosolic NADH/NAD^+^ status, LDH isoenzyme background, proton‐coupled monocarboxylate transport, HCAR1 signaling, and enzymatic/non‐enzymatic lactylation, emphasizing that AD progression may involve a loss of coordination among lactate transport, oxidation, receptor signaling, pH control, and covalent modification rather than an absolute increase in lactate itself (Zaman et al. 2026). Similarly, NAD^+^ precursor supplementation has shown evidence of ameliorating AD phenotypes in preclinical models, and NR have entered early‐stage clinical evaluation in individuals with mild cognitive impairment, yet their mechanism of action is difficult to attribute to a single histone deacetylation event (Orr et al. 2024; Wang et al. 2021). Sirtuin substrates include histones and non‐histone proteins such as RelA/p65, PGC‐1α, and TFEB, and restoration of NAD^+^ levels also affects mitochondrial function and DNA damage responses (Vinten et al. 2025). Moreover, chronic manipulation of lactate, NAD^+^, or lipid metabolism engages numerous physiological processes and lacks cell‐type resolution (Mogol et al. 2024). These interventions are therefore best deployed as biomarker‐guided adjuncts rather than standalone disease modifiers.

### 7.3 Natural Products

Natural products generally refer to secondary metabolites and endogenous bioactive molecules derived from plants, microorganisms, or marine organisms, encompassing diverse chemical classes such as polyphenols, terpenoids, and alkaloids. Their high structural diversity and broad target range have made them both an important source of drug leads and frequently used pharmacological probes for dissecting signaling pathways and epigenetic regulatory networks. In AD research, natural polyphenols such as hydroxytyrosol, curcumin, and resveratrol have been shown to modulate HDACs, SIRT1, and inflammatory pathways in cellular and animal models, though largely through pleiotropic mechanisms (Fabiani et al. 2021; Hou et al. 2024; Jiao et al. 2025; Li, Wang, et al. 2021; Puranik et al. 2025; Wang, Zhang, et al. 2022). With respect to clinical evidence, the progress of different compounds varies markedly: hydroxytyrosol currently has only preclinical AD evidence, with no completed clinical trial in AD patients (Fabiani et al. 2021); the high‐bioavailability curcumin formulation Theracurmin (180 mg/day for 18 months) improved memory in non‐demented adults and reduced PET‐detected amyloid and tau accumulation (NCT01383161), whereas conventional curcumin formulations failed in a mild‐to‐moderate AD trial (NCT00099710) (Liu et al. 2022; Perluigi et al. 2024); a Phase II trial of resveratrol reported reductions in Aβ and tau deposition in patients with mild‐to‐moderate AD, but the overall evidence has not shown consistent cognitive benefit (Turner et al. 2015). These discrepancies suggest that formulation, dose, exposure level, and study population significantly influence outcomes, and that positive imaging or biomarker signals alone are insufficient to demonstrate that a chromatin mechanism mediates the clinical effect.

The common bottleneck for clinical translation of natural products remains poor oral bioavailability, rapid metabolism, limited BBB penetration, and substantial interindividual pharmacokinetic variability (Chen, Guan, et al. 2025; Puranik et al. 2025). Therefore, a more reasonable current positioning is to regard natural products as mechanistic discovery tools for mapping epigenetic regulatory networks rather than as prioritized AD therapeutic candidates (Kumari and Zeng 2026). Fisetin, for example, has provided conceptual validation for regulatory axes such as HDAC3–NRF2 (Jo et al. 2025). Future advances in nanoparticle delivery, structural optimization, and standardized formulations may improve their drug‐likeness, but in AD they still require rigorous target engagement, locus‐resolved chromatin readouts, and clinical endpoint validation (Kumbhar et al. 2025).

### 7.4 Epigenome Editing

CRISPR/dCas9‐based epigenome editing represents an intervention logic conceptually distinct from all the strategies discussed above: rather than modulating enzyme activity or metabolite levels, it directs effector domains—such as the p300 acetyltransferase core, the KRAB repressor domain, or the DNMT3A methyltransferase domain—to specific genomic loci. On this basis, it provides the most precise theoretical framework for cell‐type‐ and locus‐specific chromatin manipulation (Khan et al. 2026; Zhou et al. 2026). Fusion of dCas9 with epigenetic effector domains enables targeted regulation of inflammatory genes and enhancers in animal models, demonstrating feasibility for remodeling pathological chromatin states (Chai et al. 2024; Kantor et al. 2024; Khan et al. 2026; Kincaid et al. 2025; Xu et al. 2024). PROTAC‐based protein degradation further offers a complementary strategy for selectively eliminating pathogenic epigenetic enzymes (Rodrigues et al. 2022; Singh et al. 2026).

In AD, APOE4‐targeted epigenome therapy is a representative case: snRNA‐seq‐guided, APOE‐targeted, microglia‐specific epigenome therapy aims to precisely and specifically reduce APOE4 expression in microglia without altering the DNA sequence (Yassine et al. 2025). The design logic of this strategy is directly related to the findings in Section 6.3 regarding APOE isoform‐dependent remodeling of the transcriptional and chromatin accessibility landscape in human microglia: if the pathogenic effects of APOE4 are mediated in part through a specific chromatin state established in microglia, then chromatin editing targeted to the APOE4 locus may offer a better benefit–risk profile than systemic APOE4 reduction.

Broader CRISPR‐based epigenome editing tools are being extended to neuroinflammatory and proteinopathy pathways. One group evaluated the therapeutic potential of CRISPR‐Cas9 and next‐generation gene editing technologies, including base editors and prime editors, in AD, covering targeting of APOE4, APP, PSEN1, PSEN2, and MAPT, as well as strategies using CRISPR interference/activation and epigenome editing tools for allele‐specific correction, gene silencing, and transcriptional regulation (Khan et al. 2026).

However, this field remains largely preclinical. Future efforts must overcome CNS delivery barriers, including engineered AAV vectors, lentiviral systems, lipid nanoparticles, polymeric nanocarriers, exosome delivery, receptor‐mediated transcytosis, immune‐evasive vector design, and focused ultrasound‐mediated BBB modulation (Dou et al. 2025; Onohuean et al. 2025; White et al. 2025).

### 7.5 Lifestyle and Diet Management

Exercise and dietary interventions indirectly affect nuclear metabolite pools and chromatin enzyme activity by altering systemic metabolic status, and can delay age‐related neuroinflammation (Chai et al. 2026; Gong et al. 2015; Li, Deng, et al. 2025). The strength of this mode of action lies in its physiological and systemic character, but its limitation is the lack of cell‐type resolution—the metabolic signals influenced by exercise or diet (lactate, β‐OHB, NAD^+^, acetyl‐CoA) reach neurons, microglia, and astrocytes with distinct kinetics and amplitudes, and the cell‐type distribution of their chromatin consequences is heterogeneous (Pang et al. 2026; Wang, Feng, et al. 2026).

Ketone body metabolism provides a concrete example linking lifestyle interventions to specific chromatin modifications. Ketogenic diets and ketone bodies act by inhibiting oxidative stress and modulating histone and non‐histone PTMs (Ravussin et al. 2015). In a randomized trial in mild cognitive impairment, a ketogenic beverage was found to aid cognitive improvement, possibly related to histone β‐hydroxybutyrylation (Fortier et al. 2021; Zhou et al. 2022); ketogenic regimens also modulate autophagy, neurotransmission, and gut microbiota to reduce neuroinflammation (Jang et al. 2023). However, caution is warranted: no clinical study has directly demonstrated that a ketogenic diet alters β‐hydroxybutyrylation levels at specific genomic loci in AD patients, let alone that such changes are causal mediators of cognitive improvement. Lifestyle measures are therefore adjuncts for epigenetic control, but effect sizes, optimal timing, and combination with pharmacological therapy remain to be systematically defined.

The study of exercise‐induced chromatin effects presents a different picture. Exercise raises circulating lactate levels, and lactate may exert neuroprotective effects after crossing the BBB (Gomez‐Pinilla and Thapak 2024); in PD, aquatic exercise improved motor performance and was accompanied by increased peripheral BDNF and histone H4 acetylation, but the small sample size precludes causal conclusions (Monteiro‐Junior et al. 2015). The difficulty in interpreting this finding lies in the fact that the relationship between exercise‐induced lactate elevation and the lactate–lactylation inflammatory program in microglia under AD pathology is complex and potentially opposite in direction. In the physiological context of exercise, lactate as an oxidative substrate and signaling molecule may promote neuroplasticity; in the pathological context of AD, lactate produced by glycolytic reprogramming in microglia drives inflammatory programs through H4K12la (Pan et al. 2022; Zong et al. 2025). Thus, the two therapeutic recommendations—“exercise increases lactate” and “inhibit lactate production to reduce neuroinflammation”—may not be contradictory, but rather reflect the functional heterogeneity of lactate signaling across different cell types, metabolic environments, and timescales. This recognition further reinforces the core argument of this review: changes in metabolite concentration alone are not a sufficient basis for therapeutic decision‐making and must be evaluated in the context of cell type, genomic localization, and downstream transcriptional consequences.

## 8 Translation and Unresolved Questions

Translating histone‐modification biology into AD diagnostics and therapies requires, above all, a shift from association to causation. The senescence‐centred framework developed in the preceding sections makes that requirement concrete: a mark is mechanistically meaningful only if it can be shown to initiate senescence entry, to sustain SASP output, or neither—if it merely reports these states (Alkhammash and Alotaibi 2025; Liu, Zhang, et al. 2025). We therefore treat three evidentiary tests as the standard for the claims reviewed here. First, perturbation: does locus‐specific alteration of a mark change senescence‐associated transcription? Second, persistence: does the resulting chromatin state survive withdrawal of the initiating stimulus—bona fide epigenetic memory—or does it exist only under continued stimulation? Third, reversibility: does erasing the mark restore homeostatic glial function? Most of the evidence discussed in this Review satisfies none of these tests fully; the technologies and biomarkers considered below, and the agenda that closes this section, are organized around closing that gap (Barberis and Xie 2026; Subasinghe et al. 2025; Yang, Lin, et al. 2026).

### 8.1 Emerging Technologies: From Descriptive Maps to Causal Inference

Cellular heterogeneity in the AD brain is now resolvable at the level of individual nuclei and of tissue neighborhoods: single‐nucleus ATAC‐seq and RNA‐seq map cell‐type‐specific programmes, spatial transcriptomics registers them against plaques and vessels, and CUT&Tag and low‐input ChIP‐seq profile marks such as H3K27ac, H3K4me3 and H3K27me3 from limited material (Barberis and Xie 2026; Kaya‐Okur et al. 2019). Single‐cell multiregion epigenomics has already shown that chromatin rewiring tracks AD progression and cognitive resilience across brain regions (Liu, Zhang, et al. 2025), and spatial‐CUT&Tag extends mark profiling to intact tissue sections at near‐cellular resolution (Deng et al. 2025). These maps are necessary but not sufficient, because they document association rather than causation. Two complementary developments address the gap. Locus‐targeted editing with CRISPR–dCas9 effectors can deposit or erase a mark at a single regulatory element and read out the transcriptional consequence; in the adult mouse brain this approach has now provided direct causal evidence that editing one locus is sufficient to alter a learned behavior, reversibly within the same animal (Coda et al. 2025). In AD models specifically, targeted methylation of the APP promoter with dCas9–Dnmt3a reduced APP expression, lowered Aβ and attenuated cognitive impairment in vivo, establishing that the approach engages disease‐relevant loci (Park et al. 2022). Spatial metabolomics—MALDI and DESI imaging—can register metabolic gradients of lactate, acetyl‐CoA precursors and lipids against chromatin landscapes around plaques, linking the metabolic nodes of Section 5 to histology (Qiao et al. 2026; Yang et al. 2025). The near‐term priority is integration: perturbation experiments performed in aged, human‐cell‐relevant systems, with mark occupancy and metabolite abundance measured in the same cells. Sample quality, cost and the lack of shared analytical standards remain the main constraints (Khan, Arslanturk, and Draghici 2025).

### 8.2 Biofluid Biomarkers: From Direct Detection to Indirect Surrogates

Histone marks are nuclear and inaccessible in the living brain, so their direct clinical use as biomarkers is limited (Deng et al. 2025); measurable surrogates are the realistic path. Three classes are being pursued, each with a distinct evidentiary status. Brain‐derived extracellular vesicles—microglial and neuronal exosomes isolated from plasma or CSF—carry cell‐type‐specific protein cargo and potentially chromatin‐associated signatures, and microfluidic isolation of neuron‐ and astrocyte‐derived vesicles has already distinguished Lewy body pathology from AD (Huang et al. 2022; Yang, Lin, et al. 2026). Metabolic intermediates form a second, more tractable class: lactate, NAD^+^‐related metabolites and acetyl‐CoA‐linked lipid species report the same nodes that drive histone acylation and deacylation, and oral nicotinamide riboside has been shown in humans to raise NAD^+^ and to alter biomarkers in neuron‐enriched plasma vesicles (Francia et al. 2025; Pourshafie et al. 2026; Vreones et al. 2023). Circulating nucleosomes and cell‐free‐chromatin assays form the third: cell‐free chromatin state tracing can now infer tissue of origin and therapy response from peripheral blood, providing proof of principle that chromatin information is accessible outside the brain (Chen, Meng, et al. 2026; Main et al. 2025). None of these readouts yet reports a specific histone mark at a defined locus in a defined glial cell type, and source specificity, analyte stability and assay standardization remain the decisive hurdles (Aydın et al. 2025). The protein‐based tests against which any chromatin‐derived marker will be judged are already demanding: plasma %p‐tau217 matches or exceeds clinically approved CSF assays for both amyloid and tau pathology (Barthélemy et al. 2024). A realistic trajectory is therefore a composite panel in which vesicle cargo, metabolite ratios and cell‐free chromatin features complement Aβ42/40, p‐tau217 and NfL rather than replace them.

### 8.3 Comparative Perspectives Across Neurodegenerative Diseases

Chronic neuroinflammation with epigenetic remodeling is not unique to AD: Parkinson's disease, Huntington's disease, amyotrophic lateral sclerosis, and multiple sclerosis all feature glial activation and altered histone‐mark patterns (Li and Barres 2018). Cross‐disease comparison separates shared programmes from disease‐specific signatures. Despite distinct initiating triggers—Aβ and tau, α‐synuclein, TDP‐43, mutant huntingtin—these disorders converge on microglial and astrocytic enhancer reprogramming: H3K27ac gains, increased accessibility at inflammatory loci and transcription‐factor reorganization, with TREM2–APOE, NF‐κB, and interferon pathways as central nodes (Jung et al. 2025; Li, Chen, et al. 2026; Murphy, Ye, et al. 2025). Metabolism‐driven marks, notably lactylation and crotonylation, recur across models, implying shared inflammatory chemistry (Arancibia‐Opazo et al. 2023). Inherited risk sits upstream of these shared programmes: in AD cortex, integrated multi‐omics has linked specific histone‐acetylation and DNA‐methylation alterations to AD risk loci, indicating that genetic susceptibility and chromatin state are not independent (Nativio et al. 2020). A dual‐layer picture follows: shared epigenetic programmes are the highest‐priority targets because interventions against them are most likely to generalize, whereas disease‐specific signatures enable precision stratification (Gopalakrishna et al. 2026; Roy et al. 2025).

The same regulatory logic extends beyond neurodegeneration. Cellular senescence and SASP‐driven inflammation are shared features of chronic inflammatory disease outside the CNS—rheumatoid arthritis, atherosclerosis, inflammatory bowel disease and chronic obstructive pulmonary disease—and histone PTMs have been implicated in each. Metabolite‐derived acylation provides a concrete parallel: in CD4+ T cells from patients with rheumatoid arthritis, succinyl‐CoA accumulation drives BRD2 succinylation and represses the proteasomal subunit PSMB5, coupling a metabolic intermediate to an inflammatory cell fate through chromatin (Wu, Su, et al. 2024). Macrophage histone lactylation, first defined in peripheral innate immunity, likewise operates in both tissue‐reparative and inflammatory programmes across organs (Zhang et al. 2019). Two transferable principles follow. First, the enzymes that install and remove inflammation‐relevant marks—p300/CBP, HDACs, sirtuins and the lysine demethylases—are common to these conditions, so pharmacological lessons on isoform selectivity, cell‐type targeting and biomarker‐guided stratification should be exchanged across fields. Second, disease specificity resides less in the enzymatic machinery than in the cell types, loci and metabolic environments on which it acts; cross‐disease epigenomic atlases of senescent cells would therefore help to distinguish shared, high‐priority targets from context‐specific ones. We anticipate that the senescence–chromatin framework developed here for AD will serve as a template for dissecting chromatin‐encoded inflammation in age‐associated inflammatory disease more broadly.

### 8.4 Outstanding Questions

The three evidentiary tests set out above define the standard; six concrete problems define the agenda. The first concerns causal hierarchy. Whether a histone‐mark change initiates, maintains, or merely accompanies an inflammatory state can be decided only by locus‐specific perturbation, and CRISPR–dCas9‐based editing has now demonstrated in vivo that targeting a single regulatory locus is sufficient to alter a learned behavior, reversibly within the same animal (Coda et al. 2025); the equivalent experiment at a senescence‐associated enhancer has not yet been performed. The second concerns coverage. Cell‐type‐ and region‐resolved epigenomic maps across disease stages remain incomplete, and the maps that exist concentrate on a small set of marks in bulk or mixed‐cell material (Liu, Zhang, et al. 2025). The third concerns persistence and reversibility. Direct evidence that a mark‐level state can outlive its trigger exists in the CNS but outside the senescence field: a single peripheral inflammatory challenge reprograms microglial enhancers in a manner that persists for at least 6 months and modifies amyloid pathology in an AD mouse model (Wendeln et al. 2018). That precedent shows persistence is achievable and, equally, that it must be measured directly rather than inferred from continued stimulation; longitudinal tracking with stimulus‐withdrawal designs is therefore required (Wu, He, et al. 2025). The fourth concerns network buffering. Crosstalk among marks, and the shared use of writers and erasers, may compensate for single‐target interventions, so network‐level strategies may be required (Lisek et al. 2025). The fifth concerns substrate attribution. The pleiotropy of p300/CBP and of the HDACs makes it difficult to separate histone from non‐histone effects of any pharmacological intervention, and conclusions drawn from enzyme inhibitors alone should be treated accordingly (Wang, Liu, et al. 2026). The sixth concerns combination design. The timing, sequencing, and safety of immunotherapy–metabolic–epigenetic combinations are undefined, and rational design will require pharmacodynamic readouts that report the state of the circuit rather than a single analyte (Ballard et al. 2025). None of these problems is unique to histone biology; what the senescence framework adds is a way to rank them because a mark that fails the persistence test cannot, by definition, be the substrate of the chronic inflammatory state.

## 9 Conclusion and Perspectives

Chronic neuroinflammation in AD is not merely a downstream consequence of amyloid‐β and tau pathology. In large part, it is the inflammatory signature of cellular senescence, written into and maintained by chromatin states that histone PTMs help establish. This epigenetic layer integrates metabolic dysfunction, mitochondrial impairment, innate immune signaling, and proteotoxic stress into self‐reinforcing transcriptional circuits. It also provides a unified explanation for three features of AD‐associated inflammation that protein‐centric models leave unresolved: its persistence over years, its cell‐type specificity, and its declining reversibility with disease stage.

The central challenge is therefore no longer descriptive annotation but causal attribution: distinguishing the chromatin marks that drive glial senescence from those that merely accompany it, and identifying the chromatin nodes that remain reversible. Three criteria—perturbation, persistence, and reversibility—provide the benchmark. When applied to the current literature, these criteria reveal a consistent pattern. Most AD evidence documents chromatin states under continued pathological stimulation; almost none demonstrates that a state survives withdrawal of the stimulus, and even fewer demonstrate that erasing the mark restores homeostatic function. Claims of irreversibility, or of locked inflammatory states, should therefore be treated as hypotheses, as we do throughout this Review.

Progress will require convergence across three experimental axes: single‐cell and spatial epigenomic mapping of senescent glia and neurons across disease trajectories; cell‐type‐specific perturbation, using CRISPR‐based epigenome editing and locus‐targeted tools, to establish causality at candidate loci; and translational biomarkers—extracellular vesicle cargo, metabolic intermediates and circulating nucleosomes—that report brain chromatin states in accessible biofluids. The same logic indicates where leverage is greatest. Because the marks that sustain senescence‐associated inflammation are deposited by metabolite‐dependent enzymes, the principal metabolic nodes—glycolysis and lactate, acetyl‐CoA, NAD^+^, and one‐carbon metabolism—are simultaneously mechanisms and measurement points. Likewise, the feedforward circuit linking proteinopathy, immune activation, and chromatin identifies the junctions at which intervention is most likely to be durable. Only through such a closed loop can histone PTMs move from correlative markers to actionable mechanistic nodes.

Therapeutically, epigenetic strategies should complement, not replace, amyloid‐ and tau‐directed approaches, with the goal of dismantling entrenched senescence–SASP circuits and restoring immune homeostasis. Among current options, metabolic–epigenetic interventions that modulate NAD^+^, lactate and acetyl‐CoA availability offer the most measurable near‐term path; isoform‐selective enzyme inhibitors have the strongest preclinical foundation; and cell‐type‐specific epigenome editing and senomorphic regimens represent the longer‐term precision strategy—although delivery, specificity, safety and monitoring remain substantial constraints in each case.

Ultimately, the question is not simply which marks change in AD, but which are functionally actionable, in which cell type and at which disease stage. If such causal maps can be drawn, chromatin‐encoded cellular senescence may emerge as a therapeutic axis running parallel to protein‐targeted strategies. Histone PTMs are not epigenetic noise: they form a structured molecular language through which the aging brain acquires and maintains its senescent, inflammatory state. Learning to rewrite that language is a central frontier for the biology of aging.

## Author Contributions

J.L. and H.C. contributed equally to this work. J.L., H.C., X.R., B.L. and W.Z. conceived the study and developed the conceptual framework. J.L., H.C., X.R., X.H. and L.J. performed literature investigation and data curation. J.L., H.C., X.R., N.S., X.H. and L.J. contributed to manuscript drafting, figure preparation and critical discussion of the literature. During revision, J.L. and H.C. led the major revisions and response to the reviewers. B.L. and W.Z. supervised the study, performed the final critical review and revised the manuscript. All authors reviewed and approved the final manuscript.

## Funding

This work was supported by the Natural Science Foundation of Heilongjiang Province, China (No. LH2023H073) and the Innovative Research Project for Postgraduate Students of Heilongjiang University of Chinese Medicine (No. 2026yjscx001).

## Ethics Statement

The authors have nothing to report.

## Consent

All authors have read and approved the final version of the manuscript and agree to its publication.

## Conflicts of Interest

The authors declare no conflicts of interest.

## Data Availability

Data sharing not applicable to this article as no datasets were generated or analyzed during the current study.

## References

[acel70785-bib-0001] Ai, H. , Z. He , Z. Deng , et al. 2024. “Structural and Mechanistic Basis for Nucleosomal H2AK119 Deubiquitination by Single‐Subunit Deubiquitinase USP16.” Nature Structural & Molecular Biology 31, no. 11: 1745–1755. 10.1038/s41594-024-01342-2. 38918638

[acel70785-bib-0002] Aird, K. M. , O. Iwasaki , A. V. Kossenkov , et al. 2016. “HMGB2 Orchestrates the Chromatin Landscape of Senescence‐Associated Secretory Phenotype Gene Loci.” Journal of Cell Biology 215, no. 3: 325–334. 10.1083/jcb.201608026. PMC5100296 27799366

[acel70785-bib-0003] Akakura, S. , and S. Tabibzadeh . 2026. “AKG‐TET Axis Is Central to Senescence Plasticity.” iScience 29, no. 1: 114298. 10.1016/j.isci.2025.114298. PMC12767804 41497192

[acel70785-bib-0004] AlHayani, D. A. , A. Kubaev , S. Uthirapathy , et al. 2025. “Insights Into the Therapeutic Potential of SIRT1‐Modifying Compounds for Alzheimer's Disease: A Focus on Molecular Mechanisms.” Journal of Molecular Neuroscience 75, no. 1: 29. 10.1007/s12031-025-02324-9. 40000535

[acel70785-bib-0005] Alkhammash, A. , and G. Alotaibi . 2025. “Epigenetic Reprogramming as a Therapeutic Strategy for Neurodegenerative Diseases: A Complex and Novel Approach.” European Journal of Pharmacology 1002: 177853. 10.1016/j.ejphar.2025.177853. 40541595

[acel70785-bib-0006] Amaral, M. L. , S. Mamde , M. Miller , et al. 2026. “Single‐Cell Epigenomics Uncovers Heterochromatin Instability and Transcription Factor Dysfunction During Mouse Brain Aging.” Cell Reports 45, no. 3: 117073. 10.1016/j.celrep.2026.117073. PMC13189690 41824460

[acel70785-bib-0007] Amniouel, S. , J. Suh , W. Zheng , and Q. Zhang . 2025. “Beyond Amyloid: Nanobody‐Mediated Neuroinflammatory Therapy for Alzheimer's Disease.” Transl Neurodegener 14, no. 1: 51. 10.1186/s40035-025-00513-5. PMC12516901 41084027

[acel70785-bib-0008] Anagho, H. A. , M. Mullari , A. G. Prósz , et al. 2024. “ADP‐Ribosylome Analysis Reveals Homogeneous DNA‐Damage‐Induced Serine ADP‐Ribosylation Across Wild‐Type and BRCA‐Mutant Breast Cancer Cell Lines.” Cell Reports 43, no. 7: 114433. 10.1016/j.celrep.2024.114433. 38985679

[acel70785-bib-0009] Arancibia‐Opazo, S. , J. S. Contreras‐Riquelme , M. Sánchez , et al. 2023. “Transcriptional and Histone Acetylation Changes Associated With CRE Elements Expose Key Factors Governing the Regulatory Circuit in the Early Stage of Huntington's Disease Models.” International Journal of Molecular Sciences 24, no. 13: 10848. 10.3390/ijms241310848. PMC10341483 37446028

[acel70785-bib-0010] Ardura‐Fabregat, A. , L. F. P. Bosch , E. Wogram , et al. 2025. “Response of Spatially Defined Microglia States With Distinct Chromatin Accessibility in a Mouse Model of Alzheimer's Disease.” Nature Neuroscience 28, no. 8: 1688–1703. 10.1038/s41593-025-02006-0. PMC12321583 40659845

[acel70785-bib-0011] Asadi, K. , R. K , S. Satarker , and P. C. Gurram . 2025. “Histone Deacetylase 2 in Alzheimer's Disease: A Comprehensive Molecular Blueprint for Therapeutic Targeting.” Molecular Neurobiology 62, no. 12: 16489–16511. 10.1007/s12035-025-05264-y. 40781362

[acel70785-bib-0012] Aydın, Ş. , S. Özdemir , and A. Adıgüzel . 2025. “The Potential of cfDNA as Biomarker: Opportunities and Challenges for Neurodegenerative Diseases.” Journal of Molecular Neuroscience 75, no. 1: 34. 10.1007/s12031-025-02317-8. PMC11906534 40080233

[acel70785-bib-0013] Balamurli, G. , A. Q. X. Liew , W. W. Tee , and S. Pervaiz . 2024. “Interplay Between Epigenetics, Senescence and Cellular Redox Metabolism in Cancer and Its Therapeutic Implications.” Redox Biology 78: 103441. 10.1016/j.redox.2024.103441. PMC11629570 39612910

[acel70785-bib-0014] Ballard, C. , J. Sultana , P. Doherty , G. Williams , and A. Corbett . 2025. “Identifying Synergistic Combinations of Repurposed Treatments for Alzheimer's Disease.” Journal of Prevention of Alzheimer's Disease 12, no. 10: 100329. 10.1016/j.tjpad.2025.100329. PMC12627884 41145339

[acel70785-bib-0015] Barberis, M. , and J. Xie . 2026. “Spatial Dynamics in Health and Disease: From Neurodevelopment to Therapeutic Target Identification for Inflammatory Diseases.” Signal Transduction and Targeted Therapy 11, no. 1: 49. 10.1038/s41392-026-02589-5. PMC12883626 41656282

[acel70785-bib-0016] Barthélemy, N. R. , G. Salvadó , S. E. Schindler , et al. 2024. “Highly Accurate Blood Test for Alzheimer's Disease Is Similar or Superior to Clinical Cerebrospinal Fluid Tests.” Nature Medicine 30, no. 4: 1085–1095. 10.1038/s41591-024-02869-z. PMC11031399 38382645

[acel70785-bib-0017] Bellver‐Sanchis, A. , D. Valle‐Garcia , C. Barbaraci , et al. 2026. “First‐In‐Class SAM‐Competitive G9a Inhibitor FLAV‐27 as a Disease‐Modifying Therapy for Alzheimer Disease.” Molecular Therapy 34, no. 4: 2372–2407. 10.1016/j.ymthe.2025.12.038. PMC13069314 41445192

[acel70785-bib-0018] Bondarev, A. D. , M. M. Attwood , J. Jonsson , V. N. Chubarev , V. V. Tarasov , and H. B. Schiöth . 2021. “Recent Developments of HDAC Inhibitors: Emerging Indications and Novel Molecules.” British Journal of Clinical Pharmacology 87, no. 12: 4577–4597. 10.1111/bcp.14889. 33971031

[acel70785-bib-0019] Butovsky, O. , M. P. Jedrychowski , C. S. Moore , et al. 2014. “Identification of a Unique TGF‐β‐Dependent Molecular and Functional Signature in Microglia.” Nature Neuroscience 17, no. 1: 131–143. 10.1038/nn.3599. PMC4066672 24316888

[acel70785-bib-0020] Butovsky, O. , N. Rosenzweig , K. L. Kleemann , et al. 2026. “Immune Dysfunction in Alzheimer Disease.” Nature Reviews Neuroscience 27, no. 3: 196–218. 10.1038/s41583-025-00997-0. PMC13010386 41315874

[acel70785-bib-0021] Cai, C. , Y. S. Chong , H. Liang , et al. 2026. “Simvastatin Rescues Cognitive Impairment in an Aβ(1‐42)‐Induced Model of Alzheimer's Disease Through the HDAC2‐BDNF Signaling Pathway.” Neuropsychopharmacology 51: 1946–1957. 10.1038/s41386-026-02440-z. PMC13598118 42141074

[acel70785-bib-0022] Cao, Q. , W. Wang , J. B. Williams , F. Yang , Z. J. Wang , and Z. Yan . 2020. “Targeting Histone K4 Trimethylation for Treatment of Cognitive and Synaptic Deficits in Mouse Models of Alzheimer's Disease.” Science Advances 6, no. 50: eabc8096. 10.1126/sciadv.abc8096. PMC7725456 33298440

[acel70785-bib-0023] Capell, B. C. , A. M. Drake , J. Zhu , et al. 2016. “MLL1 Is Essential for the Senescence‐Associated Secretory Phenotype.” Genes & Development 30, no. 3: 321–336. 10.1101/gad.271882.115. PMC4743061 26833731

[acel70785-bib-0024] Chai, G. S. , T. L. Gao , S. G. Bi , et al. 2026. “Aerobic Exercise Facilitates p300 Nuclear Translocation via ADRB2‐AMPKα Signaling, Leading to Enhanced Histone Acetylation and Mitigation of Cognitive Decline in APP/PS1 Mice.” Alzheimer's Research & Therapy 18, no. 1: 62. 10.1186/s13195-026-01983-z. PMC12990585 41668187

[acel70785-bib-0025] Chai, G. S. , J. Gong , Y. M. Mao , et al. 2024. “H3K4 Trimethylation Mediate Hyperhomocysteinemia Induced Neurodegeneration via Suppressing Histone Acetylation by ANP32A.” Molecular Neurobiology 61, no. 9: 6788–6804. 10.1007/s12035-024-03995-y. 38351418

[acel70785-bib-0026] Chandra, T. , K. Kirschner , J.‐Y. Thuret , et al. 2012. “Independence of Repressive Histone Marks and Chromatin Compaction During Senescent Heterochromatic Layer Formation.” Molecular Cell 47, no. 2: 203–214. 10.1016/j.molcel.2012.06.010. PMC3701408 22795131

[acel70785-bib-0027] Chatila, Z. K. , J. Mares , B. N. Vardarajan , W. Elyaman , V. Menon , and E. M. Bradshaw . 2024. “Microglial Activation Alters Chromatin Accessibility of AD‐Associated Transcription Factors.” Alzheimer's & Dementia 20, no. S1: e085990. 10.1002/alz.085990.

[acel70785-bib-0028] Chaubey, K. , E. Vázquez‐Rosa , S. J. Tripathi , et al. 2026. “Pharmacologic Reversal of Advanced Alzheimer's Disease in Mice and Identification of Potential Therapeutic Nodes in Human Brain.” Cell Reports Medicine 7, no. 1: 102535. 10.1016/j.xcrm.2025.102535. PMC12866132 41435831

[acel70785-bib-0029] Chen, H. , L. Ni , J. Zhang , et al. 2025. “Neutrophil Extracellular Traps Promote AIM2‐Dependent Microglial Pyroptosis Following Stroke.” Aging and Disease 17, no. 3: 1616–1633. 10.14336/ad.2024.1733. PMC13061539 40479571

[acel70785-bib-0030] Chen, H. , P. D. Ruiz , W. M. McKimpson , L. Novikov , R. N. Kitsis , and M. J. Gamble . 2015. “MacroH2A1 and ATM Play Opposing Roles in Paracrine Senescence and the Senescence‐Associated Secretory Phenotype.” Molecular Cell 59, no. 5: 719–731. 10.1016/j.molcel.2015.07.011. PMC4548812 26300260

[acel70785-bib-0031] Chen, J. , Y. Zhou , S. Mueller‐Steiner , et al. 2005. “SIRT1 Protects Against Microglia‐Dependent Amyloid‐Beta Toxicity Through Inhibiting NF‐kappaB Signaling.” Journal of Biological Chemistry 280, no. 48: 40364–40374. 10.1074/jbc.M509329200. 16183991

[acel70785-bib-0032] Chen, L. , Y. Guan , S. Wang , X. Han , F. Guo , and Y. Wang . 2025. “Engineered Nanoplatforms for Brain‐Targeted Co‐Delivery of Phytochemicals in Alzheimer's Disease: Rational Design, Blood‐Brain Barrier Penetration, and Multi‐Target Therapeutic Synergy.” Neurotherapeutics 22, no. 6: e00722. 10.1016/j.neurot.2025.e00722. PMC12664471 40813153

[acel70785-bib-0033] Chen, L. F. , Y. T. Lin , D. A. Gallegos , et al. 2019. “Enhancer Histone Acetylation Modulates Transcriptional Bursting Dynamics of Neuronal Activity‐Inducible Genes.” Cell Reports 26, no. 5: 1174–1188.e1175. 10.1016/j.celrep.2019.01.032. PMC6376993 30699347

[acel70785-bib-0034] Chen, S.‐J. , S.‐W. Yue , Y.‐P. Zhang , et al. 2026. “Post‐Translational Modifications in Metabolic Reprogramming: Implications for Metabolic Therapy and Immunotherapy in Cancer.” Signal Transduction and Targeted Therapy 11, no. 1: 314. 10.1038/s41392-026-02862-7. PMC13447842 42562812

[acel70785-bib-0035] Chen, X. , X. Meng , W. Zhang , et al. 2026. “Cell‐Free Chromatin State Tracing Reveals Disease Origin and Therapy Responses.” Nature 653, no. 8114: 599–610. 10.1038/s41586-026-10224-0. PMC13171458 41781618

[acel70785-bib-0036] Chen, Y. , C. Zhou , M. Abdelhamid , C. G. Jung , and M. Michikawa . 2023. “Inhibition of Sirt2 Decreases ApoE Secretion in Astrocytes and Microglial Cells.” Journal of Biochemistry 174, no. 5: 409–420. 10.1093/jb/mvad054. 37488092

[acel70785-bib-0037] Cheng, Y.‐H. , and M. S. Ho . 2025. “Disease‐Associated Microglia in Neurodegenerative Diseases: Friend or Foe?” PLoS Biology 23, no. 10: e3003426. 10.1371/journal.pbio.3003426. PMC12539720 41118343

[acel70785-bib-0038] Chibaya, L. , K. C. Murphy , K. D. DeMarco , et al. 2023. “EZH2 Inhibition Remodels the Inflammatory Senescence‐Associated Secretory Phenotype to Potentiate Pancreatic Cancer Immune Surveillance.” Nature Cancer 4, no. 6: 872–892. 10.1038/s43018-023-00553-8. PMC10516132 37142692

[acel70785-bib-0039] Chien, Y. , C. Scuoppo , X. Wang , et al. 2011. “Control of the Senescence‐Associated Secretory Phenotype by NF‐κB Promotes Senescence and Enhances Chemosensitivity.” Genes & Development 25, no. 20: 2125–2136. 10.1101/gad.17276711. PMC3205583 21979375

[acel70785-bib-0040] Chilton, P. M. , S. S. Ghare , B. T. Charpentier , et al. 2024. “Age‐Associated Temporal Decline in Butyrate‐Producing Bacteria Plays a Key Pathogenic Role in the Onset and Progression of Neuropathology and Memory Deficits in 3×Tg‐AD Mice.” Gut Microbes 16, no. 1: 2389319. 10.1080/19490976.2024.2389319. PMC11346541 39182227

[acel70785-bib-0041] Chung, S. , J.‐H. Jeong , J.‐C. Park , et al. 2024. “Blockade of STING Activation Alleviates Microglial Dysfunction and a Broad Spectrum of Alzheimer's Disease Pathologies.” Experimental & Molecular Medicine 56, no. 9: 1936–1951. 10.1038/s12276-024-01295-y. PMC11447230 39218977

[acel70785-bib-0042] Ciapponi, M. , E. Karlukova , S. Schkölziger , C. Benda , and J. Müller . 2024. “Structural Basis of the Histone Ubiquitination Read–Write Mechanism of RYBP–PRC1.” Nature Structural & Molecular Biology 31, no. 7: 1023–1027. 10.1038/s41594-024-01258-x. PMC11257959 38528151

[acel70785-bib-0043] Cirovic, B. , L. C. J. de Bree , L. Groh , et al. 2020. “BCG Vaccination in Humans Elicits Trained Immunity via the Hematopoietic Progenitor Compartment.” Cell Host & Microbe 28, no. 2: 322–334.e325. 10.1016/j.chom.2020.05.014. PMC7295478 32544459

[acel70785-bib-0044] Coda, D. M. , L. Watt , L. Glauser , et al. 2025. “Cell‐Type‐ and Locus‐Specific Epigenetic Editing of Memory Expression.” Nature Genetics 57, no. 11: 2661–2668. 10.1038/s41588-025-02368-y. PMC12597831 41162785

[acel70785-bib-0045] Contrepois, K. , C. Coudereau , B. A. Benayoun , et al. 2017. “Histone Variant H2A.J Accumulates in Senescent Cells and Promotes Inflammatory Gene Expression.” Nature Communications 8, no. 1: 14995. 10.1038/ncomms14995. PMC5436145 28489069

[acel70785-bib-0046] Datta, M. , O. Staszewski , E. Raschi , et al. 2018. “Histone Deacetylases 1 and 2 Regulate Microglia Function During Development, Homeostasis, and Neurodegeneration in a Context‐Dependent Manner.” Immunity 48, no. 3: 514–529.e516. 10.1016/j.immuni.2018.02.016. 29548672

[acel70785-bib-0047] Davis, N. , B. Taylor , L. Abelleira‐Hervas , et al. 2024. “Histone Deacetylase‐3 Regulates the Expression of the Amyloid Precursor Protein and Its Inhibition Promotes Neuroregenerative Pathways in Alzheimer's Disease Models.” FASEB Journal 38, no. 10: e23659. 10.1096/fj.202301762RR. 38733301

[acel70785-bib-0048] Deng, Z. , H. Chen , J. Chen , Z. Du , W. Zhou , and Z. Yuan . 2025. “Multifaceted Roles of Extracellular Vesicles in the Interplay of Neuroinflammation and Neurodegenerative Diseases.” Biochimica et Biophysica Acta (BBA) – Molecular Basis of Disease 1871, no. 7: 167960. 10.1016/j.bbadis.2025.167960. 40580685

[acel70785-bib-0049] Deng, Z. , S. Tao , Y. Du , et al. 2026. “Allosteric Activation of RNF20/RNF40–RAD6A‐Mediated H2BK120 Monoubiquitylation by H2BS112 GlcNAcylation.” Nature Chemical Biology 22, no. 5: 740–750. 10.1038/s41589-025-02109-6. 41495224

[acel70785-bib-0050] Di Fede, E. , E. Taci , S. Castiglioni , et al. 2025. “p300 Inhibition Delays Premature Cellular Senescence.” NPJ Aging 11, no. 1: 62. 10.1038/s41514-025-00251-y. PMC12246142 40640194

[acel70785-bib-0051] Di Giorgio, E. , H. Paluvai , E. Dalla , et al. 2021. “HDAC4 Degradation During Senescence Unleashes an Epigenetic Program Driven by AP‐1/p300 at Selected Enhancers and Super‐Enhancers.” Genome Biology 22, no. 1: 129. 10.1186/s13059-021-02340-z. PMC8108360 33966634

[acel70785-bib-0052] Diniz, L. P. , J. Morgado , A. P. Bergamo Araujo , et al. 2024. “Histone Deacetylase Inhibition Mitigates Cognitive Deficits and Astrocyte Dysfunction Induced by Amyloid‐Beta (Abeta) Oligomers.” British Journal of Pharmacology 181, no. 20: 4028–4049. 10.1111/bph.16439. 38936407

[acel70785-bib-0053] Diniz, L. P. , P. S. M. Pinheiro , L. S. Franco , and F. C. A. Gomes . 2025. “HDACs in the Brain: From Chromatin Remodeling to Neurodegenerative Disease.” Cells 14, no. 17: 1338. 10.3390/cells14171338. PMC12428036 40940748

[acel70785-bib-0054] Dou, D. , J. Lu , X. Chen , and X. Zhang . 2025. “Key Challenges and Optimization Practices for Non‐Clinical Evaluation of AAV Based Gene Therapy for Central Nervous System Disease.” Pharmaceutical Research 42, no. 10: 1661–1675. 10.1007/s11095-025-03931-2. 41034677

[acel70785-bib-0055] Dou, Z. , K. Ghosh , M. G. Vizioli , et al. 2017. “Cytoplasmic Chromatin Triggers Inflammation in Senescence and Cancer.” Nature 550, no. 7676: 402–406. 10.1038/nature24050. PMC5850938 28976970

[acel70785-bib-0056] Du, J. , Y. Yin , D. Wu , et al. 2024. “SIRT6 Modulates Lesion Microenvironment in LPC Induced Demyelination by Targeting Astrocytic CHI3L1.” Journal of Neuroinflammation 21, no. 1: 243. 10.1186/s12974-024-03241-1. PMC11438192 39342313

[acel70785-bib-0057] Duan, X. , Z. Xing , L. Qiao , et al. 2024. “The Role of Histone Post‐Translational Modifications in Cancer and Cancer Immunity: Functions, Mechanisms and Therapeutic Implications.” Frontiers in Immunology 15: 1495221. 10.3389/fimmu.2024.1495221. PMC11604627 39620228

[acel70785-bib-0058] Fabiani, R. , N. Vella , and P. Rosignoli . 2021. “Epigenetic Modifications Induced by Olive Oil and Its Phenolic Compounds: A Systematic Review.” Molecules 26, no. 2: 273. 10.3390/molecules26020273. PMC7826507 33430487

[acel70785-bib-0059] Fancy, N. N. , A. M. Smith , A. Caramello , et al. 2024. “Characterisation of Premature Cell Senescence in Alzheimer's Disease Using Single Nuclear Transcriptomics.” Acta Neuropathologica 147, no. 1: 78. 10.1007/s00401-024-02727-9. PMC11065703 38695952

[acel70785-bib-0060] Flamier, A. , J. El Hajjar , J. Adjaye , K. J. Fernandes , M. Abdouh , and G. Bernier . 2018. “Modeling Late‐Onset Sporadic Alzheimer's Disease Through BMI1 Deficiency.” Cell Reports 23, no. 9: 2653–2666. 10.1016/j.celrep.2018.04.097. 29847796

[acel70785-bib-0061] Fortier, M. , C. A. Castellano , V. St‐Pierre , et al. 2021. “A Ketogenic Drink Improves Cognition in Mild Cognitive Impairment: Results of a 6‐Month RCT.” Alzheimer's & Dementia 17, no. 3: 543–552. 10.1002/alz.12206. PMC8048678 33103819

[acel70785-bib-0062] Francia, M. , N. Ramesh , T. Boltz , et al. 2025. “Integrative Analysis of Cerebrospinal Fluid Biomarkers, Metabolomics, and Polygenic Risk Reveals Novel Metabolite Associations With Alzheimer's Disease.” Journal of Alzheimer's Disease 108, no. 4: 1677–1695. 10.1177/13872877251389924. PMC13247332 41201456

[acel70785-bib-0063] Frost, B. , M. Hemberg , J. Lewis , and M. B. Feany . 2014. “Tau Promotes Neurodegeneration Through Global Chromatin Relaxation.” Nature Neuroscience 17, no. 3: 357–366. 10.1038/nn.3639. PMC4012297 24464041

[acel70785-bib-0064] Galván‐Peña, S. , R. G. Carroll , C. Newman , et al. 2019. “Malonylation of GAPDH Is an Inflammatory Signal in Macrophages.” Nature Communications 10, no. 1: 338. 10.1038/s41467-018-08187-6. PMC6338787 30659183

[acel70785-bib-0065] Gao, D. , C. Li , S.‐Y. Liu , et al. 2024. “P300 Regulates Histone Crotonylation and Preimplantation Embryo Development.” Nature Communications 15, no. 1: 6418. 10.1038/s41467-024-50731-0. PMC11289097 39080296

[acel70785-bib-0066] García Fernández, F. , J. Park , C. Chapuis , et al. 2025. “Single Nucleosome Imaging Reveals Principles of Transient Multiscale Chromatin Reorganization Triggered by Histone ADP‐Ribosylation at DNA Lesions.” Nature Communications 16, no. 1: 6652. 10.1038/s41467-025-61834-7. PMC12276361 40683884

[acel70785-bib-0067] Ge, W. , C. Yu , J. Li , et al. 2023. “Basis of the H2AK119 Specificity of the Polycomb Repressive Deubiquitinase.” Nature 616, no. 7955: 176–182. 10.1038/s41586-023-05841-y. 36991118

[acel70785-bib-0068] Gomez‐Pinilla, F. , and P. Thapak . 2024. “Exercise Epigenetics Is Fueled by Cell Bioenergetics: Supporting Role on Brain Plasticity and Cognition.” Free Radical Biology and Medicine 220: 43–55. 10.1016/j.freeradbiomed.2024.04.237. PMC11144461 38677488

[acel70785-bib-0069] Gong, H. , H. Qian , R. Ertl , et al. 2015. “Histone Modifications Change With Age, Dietary Restriction and Rapamycin Treatment in Mouse Brain.” Oncotarget 6, no. 18: 15882–15890. 10.18632/oncotarget.4137. PMC4599244 26021816

[acel70785-bib-0070] Gopalakrishna, P. K. , C. M. N. Che Mohd Nassir , S. Anandan , et al. 2026. “The Immuno‐Glial Connectome in Alzheimer's Disease: Integrating Central and Peripheral Inflammatory Networks.” Cellular and Molecular Neurobiology 46, no. 1: 31. 10.1007/s10571-026-01671-4. PMC12891327 41569436

[acel70785-bib-0071] Gosselin, D. , D. Skola , N. G. Coufal , et al. 2017. “An Environment‐Dependent Transcriptional Network Specifies Human Microglia Identity.” Science 356, no. 6344: eaal3222. 10.1126/science.aal3222. PMC5858585 28546318

[acel70785-bib-0072] Gräff, J. , D. Rei , J.‐S. Guan , et al. 2012. “An Epigenetic Blockade of Cognitive Functions in the Neurodegenerating Brain.” Nature 483, no. 7388: 222–226. 10.1038/nature10849. PMC3498952 22388814

[acel70785-bib-0073] Green, G. S. , M. Fujita , H.‐S. Yang , et al. 2024. “Cellular Communities Reveal Trajectories of Brain Ageing and Alzheimer's Disease.” Nature 633, no. 8030: 634–645. 10.1038/s41586-024-07871-6. PMC11877878 39198642

[acel70785-bib-0074] Griñán‐Ferré, C. , L. Marsal‐García , A. Bellver‐Sanchis , et al. 2019. “Pharmacological Inhibition of G9a/GLP Restores Cognition and Reduces Oxidative Stress, Neuroinflammation and β‐Amyloid Plaques in an Early‐Onset Alzheimer's Disease Mouse Model.” Aging (Albany NY) 11, no. 23: 11591–11608. 10.18632/aging.102558. PMC6932909 31804189

[acel70785-bib-0075] Guo, Y. , A. A. Chomiak , Y. Hong , et al. 2022. “Histone H2A Ubiquitination Resulting From Brap Loss of Function Connects Multiple Aging Hallmarks and Accelerates Neurodegeneration.” iScience 25, no. 7: 104519. 10.1016/j.isci.2022.104519. PMC9213774 35754718

[acel70785-bib-0076] Gupta, R. , R. K. Ambasta , and P. Kumar . 2020. “Pharmacological Intervention of Histone Deacetylase Enzymes in the Neurodegenerative Disorders.” Life Sciences 243: 117278. 10.1016/j.lfs.2020.117278. 31926248

[acel70785-bib-0077] Hammond, T. R. , C. Dufort , L. Dissing‐Olesen , et al. 2019. “Single‐Cell RNA Sequencing of Microglia Throughout the Mouse Lifespan and in the Injured Brain Reveals Complex Cell‐State Changes.” Immunity 50, no. 1: 253–271.e256. 10.1016/j.immuni.2018.11.004. PMC6655561 30471926

[acel70785-bib-0078] Han, M. , W. He , W. Zhu , and L. Guo . 2025. “The Role of Protein Lactylation in Brain Health and Disease: Current Advances and Future Directions.” Cell Death Discovery 11, no. 1: 213. 10.1038/s41420-025-02408-w. PMC12043837 40307243

[acel70785-bib-0079] Hananya, N. , S. K. Daley , J. D. Bagert , and T. W. Muir . 2021. “Synthesis of ADP‐Ribosylated Histones Reveals Site‐Specific Impacts on Chromatin Structure and Function.” Journal of the American Chemical Society 143, no. 29: 10847–10852. 10.1021/jacs.1c05429. 34264659

[acel70785-bib-0080] Hayakawa, T. , M. Iwai , S. Aoki , et al. 2015. “SIRT1 Suppresses the Senescence‐Associated Secretory Phenotype Through Epigenetic Gene Regulation.” PLoS One 10, no. 1: e0116480. 10.1371/journal.pone.0116480. PMC4312089 25635860

[acel70785-bib-0081] He, L. , R. Yin , W. Hang , et al. 2024. “Oxygen Glucose Deprivation‐Induced Lactylation of H3K9 Contributes to M1 Polarization and Inflammation of Microglia Through TNF Pathway.” Biomedicine 12, no. 10: 2371. 10.3390/biomedicines12102371. PMC11504212 39457683

[acel70785-bib-0082] He, S. , X. Li , N. Mittra , et al. 2025. “Microglial cGAS Deletion Preserves Intercellular Communication and Alleviates Amyloid‐β‐Induced Pathogenesis of Alzheimer's Disease.” Advanced Science (Weinheim) 12, no. 12: e2410910. 10.1002/advs.202410910. PMC11948024 39908354

[acel70785-bib-0083] Heneka, M. T. , W. M. van der Flier , F. Jessen , et al. 2025. “Neuroinflammation in Alzheimer Disease.” Nature Reviews Immunology 25, no. 5: 321–352. 10.1038/s41577-024-01104-7. PMC13148177 39653749

[acel70785-bib-0084] Hernandez, V. G. , K. J. Lechtenberg , T. C. Peterson , et al. 2023. “Translatome Analysis Reveals Microglia and Astrocytes to Be Distinct Regulators of Inflammation in the Hyperacute and Acute Phases After Stroke.” Glia 71, no. 8: 1960–1984. 10.1002/glia.24377. PMC10330240 37067534

[acel70785-bib-0085] Hong, W. , H. Ma , Z. Li , et al. 2025. “Inhibition of EED‐Mediated Histone Methylation Alleviates Neuroinflammation by Suppressing WNT‐Mediated Dendritic Cell Migration.” Journal of Neuroinflammation 22, no. 1: 97. 10.1186/s12974-025-03429-z. PMC11963263 40169990

[acel70785-bib-0086] Hou, B. Y. , M. H. Wu , H. Y. Hsu , Y. C. Lin , and D. I. Yang . 2024. “Polysaccharides From *Basella alba* Protect Post‐Mitotic Neurons Against Cell Cycle re‐Entry and Apoptosis Induced by the Amyloid‐Beta Peptide by Blocking Sonic Hedgehog Expression.” International Journal of Molecular Sciences 25, no. 13: 7316. 10.3390/ijms25137316. PMC11242684 39000427

[acel70785-bib-0087] Hou, Y. , S. Lautrup , S. Cordonnier , et al. 2018. “NAD(+) Supplementation Normalizes Key Alzheimer's Features and DNA Damage Responses in a New AD Mouse Model With Introduced DNA Repair Deficiency.” Proceedings of the National academy of Sciences of the United States of America 115, no. 8: E1876–E1885. 10.1073/pnas.1718819115. PMC5828618 29432159

[acel70785-bib-0088] Hu, M. , X. Kang , Z. Liu , et al. 2025. “Senescent‐Like Border‐Associated Macrophages Regulate Cognitive Aging via Migrasome‐Mediated Induction of Paracrine Senescence in Microglia.” Nature Aging 5, no. 10: 2039–2054. 10.1038/s43587-025-00956-5. 40931113

[acel70785-bib-0089] Hu, X. , J. Huang , Z. Li , et al. 2024. “Lactate Promotes Microglial Scar Formation and Facilitates Locomotor Function Recovery by Enhancing Histone H4 Lysine 12 Lactylation After Spinal Cord Injury.” Journal of Neuroinflammation 21, no. 1: 193. 10.1186/s12974-024-03186-5. PMC11297795 39095832

[acel70785-bib-0090] Hu, Z. , Q. Sun , X. Liu , J. Wang , X. Jin , and J. Hu . 2026. “Lactylation in Post‐Stroke Fatigue: Linking Metabolic Dysregulation to Neuroinflammation.” Frontiers in Neuroscience 19: 1713149. 10.3389/fnins.2025.1713149. PMC12823806 41585085

[acel70785-bib-0091] Huang, M. , E. Malovic , A. Ealy , et al. 2023. “Microglial Immune Regulation by Epigenetic Reprogramming Through Histone H3K27 Acetylation in Neuroinflammation.” Frontiers in Immunology 14: 1052925. 10.3389/fimmu.2023.1052925. PMC10073546 37033967

[acel70785-bib-0092] Huang, X. , Y. Chen , Q. Xiao , X. Shang , and Y. Liu . 2024. “Chemical Inhibitors Targeting Histone Methylation Readers.” Pharmacology & Therapeutics 256: 108614. 10.1016/j.pharmthera.2024.108614. 38401773

[acel70785-bib-0093] Huang, Y. , T. A. P. Driedonks , L. Cheng , et al. 2022. “Brain Tissue‐Derived Extracellular Vesicles in Alzheimer's Disease Display Altered Key Protein Levels Including Cell Type‐Specific Markers.” Journal of Alzheimer's Disease 90, no. 3: 1057–1072. 10.3233/JAD-220322. PMC9741741 36213994

[acel70785-bib-0094] Irizarry‐Caro, R. A. , M. M. McDaniel , G. R. Overcast , V. G. Jain , T. D. Troutman , and C. Pasare . 2020. “TLR Signaling Adapter BCAP Regulates Inflammatory to Reparatory Macrophage Transition by Promoting Histone Lactylation.” Proceedings of the National academy of Sciences of the United States of America 117, no. 48: 30628–30638. 10.1073/pnas.2009778117. PMC7720107 33199625

[acel70785-bib-0095] Ito, T. , Y. V. Teo , S. A. Evans , N. Neretti , and J. M. Sedivy . 2018. “Regulation of Cellular Senescence by Polycomb Chromatin Modifiers Through Distinct DNA Damage‐ and Histone Methylation‐Dependent Pathways.” Cell Reports 22, no. 13: 3480–3492. 10.1016/j.celrep.2018.03.002. PMC5915310 29590617

[acel70785-bib-0096] Ivanov, A. , J. Pawlikowski , I. Manoharan , et al. 2013. “Lysosome‐Mediated Processing of Chromatin in Senescence.” Journal of Cell Biology 202, no. 1: 129–143. 10.1083/jcb.201212110. PMC3704985 23816621

[acel70785-bib-0097] Jang, J. , S. R. Kim , J. E. Lee , et al. 2023. “Molecular Mechanisms of Neuroprotection by Ketone Bodies and Ketogenic Diet in Cerebral Ischemia and Neurodegenerative Diseases.” International Journal of Molecular Sciences 25, no. 1: 124. 10.3390/ijms25010124. PMC10779133 38203294

[acel70785-bib-0098] Jiang, J. , S. DuBois‐Coyne , E. Nam , S. D. Whedon , K. Lee , and P. A. Cole . 2026. “Histone Modification Cross‐Talk: Analytical Tools and Molecular Mechanisms.” Biochemical Journal 483, no. 5: 713–740. 10.1042/bcj20250115. PMC13142947 41994991

[acel70785-bib-0099] Jiao, H. , X. Wang , D. Zhang , S. Zhou , and F. Gao . 2025. “Curcumin and Neuroplasticity: Epigenetic Mechanisms Underlying Cognitive Enhancement in Aging and Neurodegenerative Disorders.” Frontiers in Aging Neuroscience 17: 1592280. 10.3389/fnagi.2025.1592280. PMC12367793 40851668

[acel70785-bib-0100] Jo, J. , C. Y. Moon , J. Lee , and H. Kang . 2025. “Fisetin Attenuates Lipopolysaccharide‐Induced Neuroinflammation by Modulating the Histone Deacetylase 3–Nuclear Factor Erythroid 2–Related Factor 2 Axis.” Food Bioscience 71: 107264. 10.1016/j.fbio.2025.107264.

[acel70785-bib-0101] Joshi, N. , P. Khadse , S. Jadhav , and R. R. Alavala . 2025. “Histone Deacetylase Inhibition in Alzheimer's Disease: Molecular Mechanisms, Therapeutic Potential, and Future Perspectives.” Current Topics in Medicinal Chemistry 25, no. 28: 3215–3233. 10.2174/0115680266360664250606110220. 40530721

[acel70785-bib-0102] Jung, E. S. , H. Choi , and I. Mook‐Jung . 2025. “Decoding Microglial Immunometabolism: A New Frontier in Alzheimer's Disease Research.” Molecular Neurodegeneration 20, no. 1: 37. 10.1186/s13024-025-00825-0. PMC11948825 40149001

[acel70785-bib-0103] Kabbe, M. , E. Agirre , K. E. Carlström , et al. 2026. “Single‐Nucleus Epigenomic Profiling of the Adult Human Central Nervous System Unveils Epigenetic Memory of Developmental Programs.” Nature Neuroscience 29, no. 4: 992–1006. 10.1038/s41593-026-02208-0. PMC13061643 41857393

[acel70785-bib-0104] Kantor, B. , B. O'Donovan , J. Rittiner , et al. 2024. “The Therapeutic Implications of All‐In‐One AAV‐Delivered Epigenome‐Editing Platform in Neurodegenerative Disorders.” Nature Communications 15, no. 1: 7259. 10.1038/s41467-024-50515-6. PMC11344155 39179542

[acel70785-bib-0105] Katbe, A. , R. Hanna , A. Flamier , et al. 2026. “Epigenomic Alterations and Neural Development Anomalies in Induced Pluripotent Stem Cells From Sporadic Alzheimer's Disease.” Development 153, no. 1: dev204910. 10.1242/dev.204910. 41293951

[acel70785-bib-0106] Kawaf, R. R. , W. S. Ramadan , and R. El‐Awady . 2024. “Deciphering the Interplay of Histone Post‐Translational Modifications in Cancer: Co‐Targeting Histone Modulators for Precision Therapy.” Life Sciences 346: 122639. 10.1016/j.lfs.2024.122639. 38615747

[acel70785-bib-0107] Kaya‐Okur, H. S. , S. J. Wu , C. A. Codomo , et al. 2019. “CUT&Tag for Efficient Epigenomic Profiling of Small Samples and Single Cells.” Nature Communications 10, no. 1: 1930. 10.1038/s41467-019-09982-5. PMC6488672 31036827

[acel70785-bib-0108] Khan, M. , S. Arslanturk , and S. Draghici . 2025. “A Comprehensive Review of Spatial Transcriptomics Data Alignment and Integration.” Nucleic Acids Research 53, no. 12: gkaf536. 10.1093/nar/gkaf536. PMC12199153 40568931

[acel70785-bib-0109] Khan, M. S. , I. Zafar , A. Jamal , et al. 2026. “CRISPR‐Cas9 and Next‐Generation Gene Editing Strategies for Therapeutic Intervention of Neurodegenerative Pathways in Alzheimer's Disease: A State‐Of‐The‐Art Review.” Acta Neurologica Belgica. 10.1007/s13760-026-03033-9. 41931258

[acel70785-bib-0110] Khan, Z. U. , M. Carretero‐Rey , C. A. M. de León‐López , and I. Navarro‐Lobato . 2025. “Memory‐Associated Immediate Early Genes: Roles in Synaptic Function, Memory Processes, and Neurological Diseases.” Molecular Neurobiology 62, no. 12: 15885–15915. 10.1007/s12035-025-05203-x. PMC12559170 40715999

[acel70785-bib-0111] Kincaid, S. , G. Setenet , N. J. Preveza , et al. 2025. “Increasing H2B Monoubiquitination Improves the Transcriptome and Memory in the Aged Hippocampus.” eNeuro 12, no. 4: ENEURO.0037‐25.2025. 10.1523/eneuro.0037-25.2025. PMC11998965 40194842

[acel70785-bib-0112] Klein, H.‐U. , C. McCabe , E. Gjoneska , et al. 2019. “Epigenome‐Wide Study Uncovers Large‐Scale Changes in Histone Acetylation Driven by Tau Pathology in Aging and Alzheimer's Human Brains.” Nature Neuroscience 22, no. 1: 37–46. 10.1038/s41593-018-0291-1. PMC6516529 30559478

[acel70785-bib-0113] Kosoy, R. , J. F. Fullard , B. Zeng , et al. 2022. “Genetics of the Human Microglia Regulome Refines Alzheimer's Disease Risk Loci.” Nature Genetics 54, no. 8: 1145–1154. 10.1038/s41588-022-01149-1. PMC9388367 35931864

[acel70785-bib-0114] Krasemann, S. , C. Madore , R. Cialic , et al. 2017. “The TREM2‐APOE Pathway Drives the Transcriptional Phenotype of Dysfunctional Microglia in Neurodegenerative Diseases.” Immunity 47, no. 3: 566–581.e569. 10.1016/j.immuni.2017.08.008. PMC5719893 28930663

[acel70785-bib-0115] Kumar, V. , and S. Beck . 2025. “Cellular Insights Into Transposable Elements in Alzheimer's Disease.” Frontiers in Molecular Biosciences 12: 1642599. 10.3389/fmolb.2025.1642599. PMC12819740 41573740

[acel70785-bib-0116] Kumari, A. , and X. A. Zeng . 2026. “Dietary Bioactives in Alzheimer's Disease: A Critical Appraisal of Clinical Trials and Future Nutritional Strategies.” Nutrients 18, no. 6: 907. 10.3390/nu18060907. PMC13029159 41901082

[acel70785-bib-0117] Kumazawa, T. , Y. Xu , Y. Wang , et al. 2026. “Metformin Inhibits Nuclear Egress of Chromatin Fragments in Senescence and Aging.” Nature Aging 6, no. 2: 303–315. 10.1038/s43587-025-01048-0. PMC13148253 41545663

[acel70785-bib-0118] Kumbhar, P. S. , K. A. Kolekar , S. Vishwas , et al. 2025. “Plant‐Based Flavonoids and Their Nanoparticles: Latest Arsenal Against Alzheimer's Disease.” Drug Delivery and Translational Research 15, no. 12: 4411–4451. 10.1007/s13346-025-01906-9. 40587047

[acel70785-bib-0119] Lavin, Y. , D. Winter , R. Blecher‐Gonen , et al. 2014. “Tissue‐Resident Macrophage Enhancer Landscapes Are Shaped by the Local Microenvironment.” Cell 159, no. 6: 1312–1326. 10.1016/j.cell.2014.11.018. PMC4437213 25480296

[acel70785-bib-0120] Lee, H.‐G. , J. M. Rone , Z. Li , et al. 2024. “Disease‐Associated Astrocyte Epigenetic Memory Promotes CNS Pathology.” Nature 627, no. 8005: 865–872. 10.1038/s41586-024-07187-5. PMC11016191 38509377

[acel70785-bib-0121] Leonen, C. J. A. , M. Shimada , C. E. Weller , et al. 2021. “Sumoylation of the Human Histone H4 Tail Inhibits p300‐Mediated Transcription by RNA Polymerase II in Cellular Extracts.” eLife 10: e67952. 10.7554/eLife.67952. PMC8626089 34747692

[acel70785-bib-0122] Li, B. F. , X. Y. Chen , R. Xie , et al. 2026. “Targeting Microglia‐Mediated Neuroinflammation in Alzheimer's Disease: Mechanisms and Therapeutic Approaches.” Frontiers in Immunology 17: 1818802. 10.3389/fimmu.2026.1818802. PMC13212244 42212127

[acel70785-bib-0123] Li, C. , J. Ren , M. Zhang , et al. 2022. “The Heterogeneity of Microglial Activation and Its Epigenetic and Non‐Coding RNA Regulations in the Immunopathogenesis of Neurodegenerative Diseases.” Cellular and Molecular Life Sciences 79, no. 10: 511. 10.1007/s00018-022-04536-3. PMC11803019 36066650

[acel70785-bib-0124] Li, D. , X. Huo , L. Shen , et al. 2025. “Astrocyte Heterogeneity in Ischemic Stroke: Molecular Mechanisms and Therapeutic Targets.” Neurobiology of Disease 209: 106885. 10.1016/j.nbd.2025.106885. 40139279

[acel70785-bib-0125] Li, D. , J. Zhang , and Q. Liu . 2022. “Brain Cell Type‐Specific Cholesterol Metabolism and Implications for Learning and Memory.” Trends in Neurosciences 45, no. 5: 401–414. 10.1016/j.tins.2022.01.002. 35184896

[acel70785-bib-0126] Li, H. , B. Deng , M. Lin , L. Lai , J. Zhao , and Y. Li . 2025. “HDAC3 Epigenetic Suppression by Electroacupuncture Restores AMPA Receptor Function and Synaptic Plasticity in Alzheimer's Disease Models.” Neuromolecular Medicine 27, no. 1: 72. 10.1007/s12017-025-08892-8. 41186623

[acel70785-bib-0127] Li, J. , S. Wang , S. Zhang , et al. 2021. “Curcumin Slows the Progression of Alzheimer's Disease by Modulating Mitochondrial Stress Responses via JMJD3‐H3K27me3‐BDNF Axis.” American Journal of Translational Research 13, no. 12: 13380–13393. PMC8748089 35035682

[acel70785-bib-0128] Li, K. , X. Qiu , Q. Yang , and Z. Wang . 2025. “Histone Modifications: Key Players in Alzheimer's Disease.” Mechanisms of Ageing and Development 228: 112102. 10.1016/j.mad.2025.112102. 40907613

[acel70785-bib-0129] Li, K.‐Q. , S.‐Q. Ding , Y. Lei , et al. 2026. “Decoding the Crosstalk Between Ubiquitination and Other Post‐Translational Modifications in Cancer Immunity: From Mechanisms to Clinical Prospects.” npj Precision Oncology 10, no. 1: 306. 10.1038/s41698-026-01488-w. PMC13454567 42156901

[acel70785-bib-0130] Li, L. , T. Xiang , J. Guo , et al. 2024. “Inhibition of ACSS2‐Mediated Histone Crotonylation Alleviates Kidney Fibrosis via IL‐1β‐Dependent Macrophage Activation and Tubular Cell Senescence.” Nature Communications 15, no. 1: 3200. 10.1038/s41467-024-47315-3. PMC11016098 38615014

[acel70785-bib-0131] Li, N. , J. Du , Y. Yang , et al. 2025. “Microglial PCGF1 Alleviates Neuroinflammation Associated Depressive Behavior in Adolescent Mice.” Molecular Psychiatry 30, no. 3: 914–926. 10.1038/s41380-024-02714-2. PMC11835731 39215186

[acel70785-bib-0132] Li, P. , Y. Wang , Y. Xu , et al. 2025. “Histone H3 Lysine 18 Lactylation Attenuates Neuroinflammation and Neurological Damage by Regulating Microglial plxnb2 After Ischemic Stroke.” Neurobiology of Disease 215: 107061. 10.1016/j.nbd.2025.107061. 40835170

[acel70785-bib-0133] Li, Q. , and B. A. Barres . 2018. “Microglia and Macrophages in Brain Homeostasis and Disease.” Nature Reviews Immunology 18, no. 4: 225–242. 10.1038/nri.2017.125. 29151590

[acel70785-bib-0134] Li, X. , J. Zhang , D. Li , et al. 2021. “Astrocytic ApoE Reprograms Neuronal Cholesterol Metabolism and Histone‐Acetylation‐Mediated Memory.” Neuron 109, no. 6: 957–970.e958. 10.1016/j.neuron.2021.01.005. 33503410

[acel70785-bib-0135] Li, Y. , T. Izhar , and T. Kanekiyo . 2025. “HDAC3 as an Emerging Therapeutic Target for Alzheimer's Disease and Other Neurological Disorders.” Molecular Neurobiology 62, no. 8: 9573–9585. 10.1007/s12035-025-04866-w. PMC12289425 40126601

[acel70785-bib-0136] Li, Y. , A. Liu , K. Chen , et al. 2024. “Sodium Butyrate Alleviates Lead‐Induced Neuroinflammation and Improves Cognitive and Memory Impairment Through the ACSS2/H3K9ac/BDNF Pathway.” Environment International 184: 108479. 10.1016/j.envint.2024.108479. 38340407

[acel70785-bib-0137] Li, Y. , Y. Zhao , X. Li , et al. 2022. “Biological and Therapeutic Role of LSD1 in Alzheimer's Diseases.” Frontiers in Pharmacology 13: 1020556. 10.3389/fphar.2022.1020556. PMC9640401 36386192

[acel70785-bib-0138] Liddelow, S. A. , K. A. Guttenplan , L. E. Clarke , et al. 2017. “Neurotoxic Reactive Astrocytes Are Induced by Activated Microglia.” Nature 541, no. 7638: 481–487. 10.1038/nature21029. PMC5404890 28099414

[acel70785-bib-0139] Lin, P. , Y. Qi , H. Chu , et al. 2025. “PFKM Phosphorylates Histone H3 and Promotes Mitotic Progression by Sensing the Levels of Citrate.” Nature Communications 16, no. 1: 6736. 10.1038/s41467-025-62111-3. PMC12283967 40695785

[acel70785-bib-0140] Lisek, M. , N. Bochenska , J. Tomczak , J. Duraj , and T. Boczek . 2025. “Epigenetic Regulation in Ischemic Neuroprotection: The Dual Role of HDACs and HATs in Neuroinflammation and Recovery.” Antioxidants (Basel) 14, no. 8: 1015. 10.3390/antiox14081015. PMC12382868 40867911

[acel70785-bib-0141] Lithner, C. U. , P. N. Lacor , W.‐Q. Zhao , et al. 2013. “Disruption of Neocortical Histone H3 Homeostasis by Soluble Aβ: Implications for Alzheimer's Disease.” Neurobiology of Aging 34, no. 9: 2081–2090. 10.1016/j.neurobiolaging.2012.12.028. 23582659

[acel70785-bib-0142] Liu, P. , Y. Wang , Y. Sun , and G. Peng . 2022. “Neuroinflammation as a Potential Therapeutic Target in Alzheimer's Disease.” Clinical Interventions in Aging 17: 665–674. 10.2147/CIA.S357558. PMC9064449 35520949

[acel70785-bib-0143] Liu, R. , X. Ren , Y. E. Park , et al. 2025. “Nuclear GTPSCS Functions as a Lactyl‐CoA Synthetase to Promote Histone Lactylation and Gliomagenesis.” Cell Metabolism 37, no. 2: 377–394.e379. 10.1016/j.cmet.2024.11.005. PMC11798710 39642882

[acel70785-bib-0144] Liu, S. , W. Cao , Y. Niu , et al. 2021. “Single‐PanIN‐Seq Unveils That ARID1A Deficiency Promotes Pancreatic Tumorigenesis by Attenuating KRAS‐Induced Senescence.” eLife 10: e64204. 10.7554/eLife.64204. PMC8203294 33983114

[acel70785-bib-0145] Liu, Y. , V. Molchanov , Y. Zhao , et al. 2025. “H3K9me3 Loss and ERVs Activation as Hallmarks for Osteoarthritis Progression and Knee Joint Aging.” Osteoarthritis and Cartilage 33, no. 1: 128–133. 10.1016/j.joca.2024.08.004. PMC11663117 39153567

[acel70785-bib-0146] Liu, Y. , Y. Ye , M. Fan , H. Y. Cheng , S. Sun , and Z. Qiu . 2026. “Epigenetic Control of Microglial Mitochondrial Immunity by KAT7 Drives Alzheimer's Disease Pathogenesis.” Neuron. 10.1016/j.neuron.2026.05.015. PMC13256259 42263678

[acel70785-bib-0147] Liu, Z. , S. Zhang , B. T. James , et al. 2025. “Single‐Cell Multiregion Epigenomic Rewiring in Alzheimer's Disease Progression and Cognitive Resilience.” Cell 188, no. 18: 4980–5002.e4929. 10.1016/j.cell.2025.06.031. PMC12573303 40752494

[acel70785-bib-0148] Longarini, E. J. , H. Dauben , C. Locatelli , et al. 2023. “Modular Antibodies Reveal DNA Damage‐Induced Mono‐ADP‐Ribosylation as a Second Wave of PARP1 Signaling.” Molecular Cell 83, no. 10: 1743–1760.e1711. 10.1016/j.molcel.2023.03.027. PMC10205078 37116497

[acel70785-bib-0149] Love, L. , B. B. Jütte , B. Lindqvist , et al. 2025. “PADI4‐Mediated Citrullination of Histone H3 Stimulates HIV‐1 Transcription.” Nature Communications 16, no. 1: 5393. 10.1038/s41467-025-61029-0. PMC12198384 40562767

[acel70785-bib-0150] Lu, P. , Y.‐T. Chan , C.‐F. Kwok , et al. 2026. “Histone Modifications Across Cancers: Mechanisms, Therapy and Clinical Translation.” Molecular Cancer 25, no. 1: 193. 10.1186/s12943-026-02707-5. PMC13464851 42243927

[acel70785-bib-0151] Lu, X. , Y. Deng , D. Yu , et al. 2014. “Histone Acetyltransferase p300 Mediates Histone Acetylation of PS1 and BACE1 in a Cellular Model of Alzheimer's Disease.” PLoS One 9, no. 7: e103067. 10.1371/journal.pone.0103067. PMC4106886 25051175

[acel70785-bib-0152] Luchsinger, J. A. , T. Perez , H. Chang , et al. 2016. “Metformin in Amnestic Mild Cognitive Impairment: Results of a Pilot Randomized Placebo Controlled Clinical Trial.” Journal of Alzheimer's Disease 51, no. 2: 501–514. 10.3233/jad-150493. PMC5079271 26890736

[acel70785-bib-0153] Lv, M. , Z. Duan , J. Tan , et al. 2025. “PHGDH‐Mediated Serine Synthesis in Astrocytes Supports Neuroinflammation by Sustaining NADH Level to Promote Histone Acetylation.” Cell Death & Disease 16, no. 1: 397. 10.1038/s41419-025-07732-8. PMC12086227 40383841

[acel70785-bib-0154] Lv, P. , X. Yang , and J. Du . 2024. “LKRSDH‐Dependent Histone Modifications of Insulin‐Like Peptide Sites Contribute to Age‐Related Circadian Rhythm Changes.” Nature Communications 15, no. 1: 3336. 10.1038/s41467-024-47740-4. PMC11026460 38637528

[acel70785-bib-0155] Main, S. C. , S. D. De Michino , L. Penny , et al. 2025. “Simulating Cell‐Free Chromatin Using Preclinical Cancer Models for Liquid Biopsy Applications.” iScience 28, no. 12: 114113. 10.1016/j.isci.2025.114113. PMC12767190 41497400

[acel70785-bib-0156] Maity, S. , K. Farrell , S. Navabpour , S. N. Narayanan , and T. J. Jarome . 2021. “Epigenetic Mechanisms in Memory and Cognitive Decline Associated With Aging and Alzheimer's Disease.” International Journal of Molecular Sciences 22, no. 22: 12280. 10.3390/ijms222212280. PMC8618067 34830163

[acel70785-bib-0157] Malik, A. N. , T. Vierbuchen , M. Hemberg , et al. 2014. “Genome‐Wide Identification and Characterization of Functional Neuronal Activity‐Dependent Enhancers.” Nature Neuroscience 17, no. 10: 1330–1339. 10.1038/nn.3808. PMC4297619 25195102

[acel70785-bib-0158] Malone, K. L. , A. K. Singh , J. M. Lignos , et al. 2026. “Unraveling the Complexity of the Histone Code: Implications for Gene Regulation and Disease.” Genome Biology 27, no. 1: 150. 10.1186/s13059-026-04011-3. PMC13134275 41877173

[acel70785-bib-0159] Mancino, S. , M. Boraso , A. Galmozzi , et al. 2025. “Dose‐Dependent Dual Effects of HDAC Inhibitors on Glial Inflammatory Response.” Scientific Reports 15, no. 1: 12262. 10.1038/s41598-025-96241-x. PMC11986048 40211035

[acel70785-bib-0160] Martini, H. , J. Birch , F. D. M. Marques , et al. 2026. “Mitochondrial Metabolism and Epigenetic Crosstalk Drive SASP.” Nature 656, no. 8129: 980–992. 10.1038/s41586-026-10791-2. PMC13518223 42527602

[acel70785-bib-0161] Marzi, S. J. , S. K. Leung , T. Ribarska , et al. 2018. “A Histone Acetylome‐Wide Association Study of Alzheimer's Disease Identifies Disease‐Associated H3K27ac Differences in the Entorhinal Cortex.” Nature Neuroscience 21, no. 11: 1618–1627. 10.1038/s41593-018-0253-7. 30349106

[acel70785-bib-0162] Mattiroli, F. , and L. Penengo . 2021. “Histone Ubiquitination: An Integrative Signaling Platform in Genome Stability.” Trends in Genetics 37, no. 6: 566–581. 10.1016/j.tig.2020.12.005. 33485674

[acel70785-bib-0163] McClarty, B. , R. Monteiro , and H. Dong . 2026. “Histone Deacetylase Inhibitors Improve Memory Function and Decrease Neuropathology in APP/PS1 Mice.” Research Square. 10.21203/rs.3.rs-10816126/v1.

[acel70785-bib-0164] Mews, P. , G. Donahue , A. M. Drake , V. Luczak , T. Abel , and S. L. Berger . 2017. “Acetyl‐CoA Synthetase Regulates Histone Acetylation and Hippocampal Memory.” Nature 546, no. 7658: 381–386. 10.1038/nature22405. PMC5505514 28562591

[acel70785-bib-0165] Min, S.‐W. , X. Chen , T. E. Tracy , et al. 2015. “Critical Role of Acetylation in Tau‐Mediated Neurodegeneration and Cognitive Deficits.” Nature Medicine 21, no. 10: 1154–1162. 10.1038/nm.3951. PMC4598295 26390242

[acel70785-bib-0166] Miziak, P. , M. Baran , L. Borkiewicz , T. Trombik , and A. Stepulak . 2024. “Acetylation of Histone H3 in Cancer Progression and Prognosis.” International Journal of Molecular Sciences 25, no. 20: 10982. 10.3390/ijms252010982 PMC11507103 39456765

[acel70785-bib-0167] Mogol, A. N. , A. Z. Kaminsky , D. J. Dutton , and Z. Madak Erdogan . 2024. “Targeting NAD+ Metabolism: Preclinical Insights Into Potential Cancer Therapy Strategies.” Endocrinology 165, no. 5: bqae043. 10.1210/endocr/bqae043. 38565429

[acel70785-bib-0168] Monteiro‐Junior, R. S. , T. Cevada , B. R. R. Oliveira , et al. 2015. “We Need to Move More: Neurobiological Hypotheses of Physical Exercise as a Treatment for Parkinson's Disease.” Medical Hypotheses 85, no. 5: 537–541. 10.1016/j.mehy.2015.07.011. 26209418

[acel70785-bib-0169] Moraitis, S. , and C. Piperi . 2025. “Multi‐Faceted Role of Histone Methyltransferase Enhancer of Zeste 2 (EZH2) in Neuroinflammation and Emerging Targeting Options.” Biology‐Basel 14, no. 7: 749. 10.3390/biology14070749. PMC12292988 40723311

[acel70785-bib-0170] Morales Pantoja, I. E. , and C. D. Mintz . 2026. “Astrocyte Epigenetics as a Priority Area in Neuroscience Research.” Frontiers in Molecular Neuroscience 18: 1716805. 10.3389/fnmol.2025.1716805. PMC12833077 41602160

[acel70785-bib-0171] Moreno‐Yruela, C. , D. Zhang , W. Wei , et al. 2022. “Class I Histone Deacetylases (HDAC1‐3) Are Histone Lysine Delactylases.” Science Advances 8, no. 3: eabi6696. 10.1126/sciadv.abi6696. PMC8769552 35044827

[acel70785-bib-0172] Moussa, C. , M. Hebron , X. Huang , et al. 2017. “Resveratrol Regulates Neuro‐Inflammation and Induces Adaptive Immunity in Alzheimer's Disease.” Journal of Neuroinflammation 14, no. 1: 1. 10.1186/s12974-016-0779-0. PMC5234138 28086917

[acel70785-bib-0173] Mu, M. D. , Z. M. Qian , S. X. Yang , K. L. Rong , W. H. Yung , and Y. Ke . 2020. “Therapeutic Effect of a Histone Demethylase Inhibitor in Parkinson's Disease.” Cell Death & Disease 11, no. 10: 927. 10.1038/s41419-020-03105-5. PMC7595123 33116116

[acel70785-bib-0174] Murphy, K. B. , D. Hu , L. Wolfs , et al. 2025. “The APOE Isoforms Differentially Shape the Transcriptomic and Epigenomic Landscapes of Human Microglia Xenografted Into a Mouse Model of Alzheimer's Disease.” Nature Communications 16, no. 1: 4883. 10.1038/s41467-025-60099-4. PMC12106835 40419479

[acel70785-bib-0175] Murphy, K. B. , Y. Ye , M. Tsalenchuk , A. Nott , and S. J. Marzi . 2025. “CHAS Infers Cell Type‐Specific Signatures in Bulk Brain Histone Acetylation Studies of Neurological and Psychiatric Disorders.” Cell Reports Methods 5, no. 5: 101032. 10.1016/j.crmeth.2025.101032. PMC12146652 40300607

[acel70785-bib-0176] Nakamura, A. , S. Sakai , Y. Taketomi , et al. 2023. “PLA2G2E‐Mediated Lipid Metabolism Triggers Brain‐Autonomous Neural Repair After Ischemic Stroke.” Neuron 111, no. 19: 2995–3010.e2999. 10.1016/j.neuron.2023.06.024. 37490917

[acel70785-bib-0177] Narita, M. , S. Nuñez , E. Heard , et al. 2003. “Rb‐Mediated Heterochromatin Formation and Silencing of E2F Target Genes During Cellular Senescence.” Cell 113, no. 6: 703–716. 10.1016/S0092-8674(03)00401-X. 12809602

[acel70785-bib-0178] Narita, T. , B. T. Weinert , and C. Choudhary . 2019. “Functions and Mechanisms of Non‐Histone Protein Acetylation.” Nature Reviews Molecular Cell Biology 20, no. 3: 156–174. 10.1038/s41580-018-0081-3. 30467427

[acel70785-bib-0179] Nativio, R. , G. Donahue , A. Berson , et al. 2018. “Dysregulation of the Epigenetic Landscape of Normal Aging in Alzheimer's Disease.” Nature Neuroscience 21, no. 4: 497–505. 10.1038/s41593-018-0101-9. PMC6124498 29507413

[acel70785-bib-0180] Nativio, R. , Y. Lan , G. Donahue , et al. 2020. “An Integrated Multi‐Omics Approach Identifies Epigenetic Alterations Associated With Alzheimer's Disease.” Nature Genetics 52, no. 10: 1024–1035. 10.1038/s41588-020-0696-0. PMC8098004 32989324

[acel70785-bib-0181] Neal, M. , and J. R. Richardson . 2018. “Epigenetic Regulation of Astrocyte Function in Neuroinflammation and Neurodegeneration.” Biochimica et Biophysica Acta—Molecular Basis of Disease 1864, no. 2: 432–443. 10.1016/j.bbadis.2017.11.004. PMC5743548 29113750

[acel70785-bib-0182] Newman, A. G. , J. Sharif , P. Bessa , et al. 2025. “Glial Reactivity and Cognitive Decline Follow Chronic Heterochromatin Loss in Neurons.” Nature Communications 16, no. 1: 7325. 10.1038/s41467-025-61319-7. PMC12334701 40781223

[acel70785-bib-0183] Ng, P. Y. , C. Zhang , H. Li , and D. J. Baker . 2023. “Senescent Microglia Represent a Subset of Disease‐Associated Microglia in P301S Mice.” Journal of Alzheimer's Disease: JAD 95, no. 2: 493–507. 10.3233/jad-230109. PMC10848894 37545233

[acel70785-bib-0184] Nirakis, N. , S. Dimothyra , E. Karadima , and V. I. Alexaki . 2025. “Metabolic Regulation of Immune Memory and Function of Microglia.” eLife 14: e107552. 10.7554/eLife.107552. PMC12513719 41071595

[acel70785-bib-0185] Niu, Z. , C. Chen , S. Wang , et al. 2024. “HBO1 Catalyzes Lysine Lactylation and Mediates Histone H3K9la to Regulate Gene Transcription.” Nature Communications 15, no. 1: 3561. 10.1038/s41467-024-47900-6. PMC11053077 38670996

[acel70785-bib-0186] Niu, Z. , C. Liu , Y. Fan , and L. Gu . 2026. “Histone Modification Clocks for Robust Cross‐Species Biological Age Prediction and Elucidating Senescence Regulation.” Proceedings of the National academy of Sciences of the United States of America 123, no. 11: e2533687123. 10.1073/pnas.2533687123. PMC12993953 41805570

[acel70785-bib-0187] Noh, J. , J. Lee , C. You , and K. Kang . 2026. “Metabolic–Epigenetic Crosstalk in Innate Immune Cell Plasticity Within the Tumor Microenvironment.” Experimental & Molecular Medicine 58, no. 8: 2491–2506. 10.1038/s12276-026-01802-3. PMC13538618 42562887

[acel70785-bib-0188] Novakovic, B. , E. Habibi , S.‐Y. Wang , et al. 2016. “β‐Glucan Reverses the Epigenetic State of LPS‐Induced Immunological Tolerance.” Cell 167, no. 5: 1354–1368.e1314. 10.1016/j.cell.2016.09.034. PMC5927328 27863248

[acel70785-bib-0189] Olawade, D. B. , I. Rashad , E. Egbon , J. Teke , S. V. Ovsepian , and S. Boussios . 2025. “Reversing Epigenetic Dysregulation in Neurodegenerative Diseases: Mechanistic and Therapeutic Considerations.” International Journal of Molecular Sciences 26, no. 10: 4929. 10.3390/ijms26104929. PMC12112518 40430067

[acel70785-bib-0190] Onishi, S. , K. Uchiyama , K. Sato , et al. 2024. “Structure of the Human Bre1 Complex Bound to the Nucleosome.” Nature Communications 15, no. 1: 2580. 10.1038/s41467-024-46910-8. PMC10959955 38519511

[acel70785-bib-0191] Onohuean, H. , S. P. Naik Bukke , C. Thalluri , K. S. Abass , and Y. E. Choonara . 2025. “Exosome Engineering for Targeted Therapy of Brain‐Infecting Pathogens: Molecular Tools, Delivery Platforms, and Translational Advances.” Frontiers in Medical Technology 7: 1655471. 10.3389/fmedt.2025.1655471. PMC12528093 41113669

[acel70785-bib-0192] Orr, M. E. , E. Kotkowski , P. Ramirez , et al. 2024. “A Randomized Placebo‐Controlled Trial of Nicotinamide Riboside in Older Adults With Mild Cognitive Impairment.” Geroscience 46, no. 1: 665–682. 10.1007/s11357-023-00999-9. PMC10828186 37994989

[acel70785-bib-0193] Oss‐Ronen, L. , T. Sarusi , and I. Cohen . 2022. “Histone Mono‐Ubiquitination in Transcriptional Regulation and Its Mark on Life: Emerging Roles in Tissue Development and Disease.” Cells 11, no. 15: 2404. 10.3390/cells11152404 PMC9368181 35954248

[acel70785-bib-0194] O'Sullivan, R. J. , S. Kubicek , S. L. Schreiber , and J. Karlseder . 2010. “Reduced Histone Biosynthesis and Chromatin Changes Arising From a Damage Signal at Telomeres.” Nature Structural & Molecular Biology 17, no. 10: 1218–1225. 10.1038/nsmb.1897. PMC2951278 20890289

[acel70785-bib-0195] Özdemir, C. , L. R. Purkey , A. Sanchez , and K. M. Miller . 2024. “PARticular MARks: Histone ADP‐Ribosylation and the DNA Damage Response.” DNA Repair (Amst) 140: 103711. 10.1016/j.dnarep.2024.103711. PMC11877395 38924925

[acel70785-bib-0196] Paiva, I. , J. Seguin , I. Grgurina , et al. 2024. “Dysregulated Expression of Cholesterol Biosynthetic Genes in Alzheimer's Disease Alters Epigenomic Signatures of Hippocampal Neurons.” Neurobiology of Disease 198: 106538. 10.1016/j.nbd.2024.106538. 38789057

[acel70785-bib-0197] Palomer, E. , N. Martín‐Flores , S. Jolly , et al. 2022. “Epigenetic Repression of Wnt Receptors in AD: A Role for Sirtuin2‐Induced H4K16ac Deacetylation of Frizzled1 and Frizzled7 Promoters.” Molecular Psychiatry 27, no. 7: 3024–3033. 10.1038/s41380-022-01492-z. PMC9205772 35296808

[acel70785-bib-0198] Pan, R. Y. , L. He , J. Zhang , et al. 2022. “Positive Feedback Regulation of Microglial Glucose Metabolism by Histone H4 Lysine 12 Lactylation in Alzheimer's Disease.” Cell Metabolism 34, no. 4: 634–648.e636. 10.1016/j.cmet.2022.02.013. 35303422

[acel70785-bib-0199] Pang, Y. , J. Yang , J. Liu , Z. Xie , and J. Wang . 2026. “Metabolic Interactions in the Brain: The Crucial Roles of Neurons, Astrocytes, and Microglia in Health and Disease.” Frontiers in Neuroscience 20: 1731771. 10.3389/fnins.2026.1731771. PMC12909589 41710159

[acel70785-bib-0200] Paniri, A. , M. M. Hosseini , and H. Akhavan‐Niaki . 2024. “Alzheimer's Disease‐Related Epigenetic Changes: Novel Therapeutic Targets.” Molecular Neurobiology 61, no. 3: 1282–1317. 10.1007/s12035-023-03626-y. 37700216

[acel70785-bib-0201] Park, H. , J. Shin , Y. Kim , T. Saito , T. C. Saido , and J. Kim . 2022. “CRISPR/dCas9‐Dnmt3a‐Mediated Targeted DNA Methylation of APP Rescues Brain Pathology in a Mouse Model of Alzheimer's Disease.” Translational Neurodegeneration 11, no. 1: 41. 10.1186/s40035-022-00314-0. PMC9476342 36109806

[acel70785-bib-0202] Park, S. , J. H. Cho , J.‐H. Kim , and J.‐A. Kim . 2024. “Histone Lysine Methylation Modifiers Controlled by Protein Stability.” Experimental & Molecular Medicine 56, no. 10: 2127–2144. 10.1038/s12276-024-01329-5. PMC11541785 39394462

[acel70785-bib-0203] Parkhurst, C. N. , G. Yang , I. Ninan , et al. 2013. “Microglia Promote Learning‐Dependent Synapse Formation Through Brain‐Derived Neurotrophic Factor.” Cell 155, no. 7: 1596–1609. 10.1016/j.cell.2013.11.030. PMC4033691 24360280

[acel70785-bib-0204] Parry, A. J. , M. Hoare , D. Bihary , et al. 2018. “NOTCH‐Mediated Non‐Cell Autonomous Regulation of Chromatin Structure During Senescence.” Nature Communications 9, no. 1: 1840. 10.1038/s41467-018-04283-9. PMC5943456 29743479

[acel70785-bib-0205] Pasini, D. , M. Malatesta , H. R. Jung , et al. 2010. “Characterization of an Antagonistic Switch Between Histone H3 Lysine 27 Methylation and Acetylation in the Transcriptional Regulation of Polycomb Group Target Genes.” Nucleic Acids Research 38, no. 15: 4958–4969. 10.1093/nar/gkq244. PMC2926606 20385584

[acel70785-bib-0206] Peng, C. , Z. Lu , Z. Xie , et al. 2011. “The First Identification of Lysine Malonylation Substrates and Its Regulatory Enzyme.” Molecular & Cellular Proteomics 10, no. 12: M111.012658. 10.1074/mcp.M111.012658. PMC3237090 21908771

[acel70785-bib-0207] Perez J, M. J. , A. Lam , C. Weissleder , et al. 2026. “The Mitochondrial Unfolded Protein Response in Human Microglia Disrupts Neuronal–Glial Communication and Promotes Senescence.” Nature Neuroscience 29, no. 8: 1858–1872. 10.1038/s41593-026-02320-1. PMC13433254 42362883

[acel70785-bib-0208] Perluigi, M. , F. Di Domenico , and D. A. Butterfield . 2024. “Oxidative Damage in Neurodegeneration: Roles in the Pathogenesis and Progression of Alzheimer Disease.” Physiological Reviews 104, no. 1: 103–197. 10.1152/physrev.00030.2022. PMC11281823 37843394

[acel70785-bib-0209] Pinheiro, P. S. M. , F. de Chirico , M. Loi , et al. 2025. “Design, Synthesis and Pharmacological Evaluation of Multitarget GPR40 Agonists/HDAC6 Inhibitors for Alzheimer's Disease.” European Journal of Medicinal Chemistry 296: 117868. 10.1016/j.ejmech.2025.117868. 40541115

[acel70785-bib-0210] Pinheiro, P. S. M. , L. P. Diniz , L. S. Franco , M. Siqueira , and F. C. A. Gomes . 2025. “Histone Deacetylases in Neurodegenerative Diseases and Their Potential Role as Therapeutic Targets: Shedding Light on Astrocytes.” Pharmaceuticals 18, no. 10: 1471. 10.3390/ph18101471. PMC12567126 41155586

[acel70785-bib-0211] Policarpi, C. , M. Munafò , S. Tsagkris , V. Carlini , and J. A. Hackett . 2024. “Systematic Epigenome Editing Captures the Context‐Dependent Instructive Function of Chromatin Modifications.” Nature Genetics 56, no. 6: 1168–1180. 10.1038/s41588-024-01706-w. PMC11176084 38724747

[acel70785-bib-0212] Pourshafie, N. , D. C. Alexander , H. Xu , et al. 2026. “ACSS2 Upregulation Enhances Neuronal Resilience to Aging and Tau‐Associated Neurodegeneration.” Proceedings of the National academy of Sciences of the United States of America 123, no. 2: e2503834122. 10.1073/pnas.2503834122. PMC12799108 41512015

[acel70785-bib-0213] Pulya, S. , S. A. Amin , N. Adhikari , S. Biswas , T. Jha , and B. Ghosh . 2021. “HDAC6 as Privileged Target in Drug Discovery: A Perspective.” Pharmacological Research 163: 105274. 10.1016/j.phrs.2020.105274. 33171304

[acel70785-bib-0214] Puranik, N. , M. Kumari , S. Tiwari , T. Dhakal , and M. Song . 2025. “Resveratrol as a Therapeutic Agent in Alzheimer's Disease: Evidence From Clinical Studies.” Nutrients 17, no. 15: 2557. 10.3390/nu17152557. PMC12348645 40806141

[acel70785-bib-0215] Qiao, Y. , J. Yin , S. Lu , and L. Yin . 2026. “Research Progress on the Application of Mass Spectrometry Imaging Technology in Cerebral Disease.” Life (Basel) 16, no. 1: 168. 10.3390/life16010168. PMC12842893 41598322

[acel70785-bib-0216] Qin, Q. , D. Wang , Y. Qu , et al. 2025. “Enhanced Glycolysis‐Derived Lactate Promotes Microglial Activation in Parkinson's Disease via Histone Lactylation.” npj Parkinson's Disease 11, no. 1: 3. 10.1038/s41531-024-00858-0. PMC11698869 39753581

[acel70785-bib-0217] Qin, Y. , P. Yang , W. He , et al. 2024. “Novel Histone Post‐Translational Modifications in Alzheimer's Disease: Current Advances and Implications.” Clinical Epigenetics 16, no. 1: 39. 10.1186/s13148-024-01650-w. PMC10924326 38461320

[acel70785-bib-0218] Rachmian, N. , S. Medina , U. Cherqui , et al. 2024. “Identification of Senescent, TREM2‐Expressing Microglia in Aging and Alzheimer's Disease Model Mouse Brain.” Nature Neuroscience 27, no. 6: 1116–1124. 10.1038/s41593-024-01620-8. 38637622

[acel70785-bib-0219] Rahimpour, S. , B. L. Clary , S. Nasoohi , Y. S. Berhanu , and C. M. Brown . 2024. “Immunometabolism in Brain Aging and Neurodegeneration: Bridging Metabolic Pathways and Immune Responses.” Aging and Disease 16, no. 6: 3361–3380. 10.14336/ad.2024.1293. PMC12539545 39751865

[acel70785-bib-0220] Rahman, M. A. , M. T. Khatun , A. K. Mim , et al. 2025. “Oxidative Stress in DNA Damage and Neurodegenerative Diseases: Unveiling the Mechanisms and Therapeutic Opportunities.” Cell Biochemistry and Biophysics 83, no. 4: 4233–4252. 10.1007/s12013-025-01845-9. 40751885

[acel70785-bib-0221] Ravussin, E. , L. M. Redman , J. Rochon , et al. 2015. “A 2‐Year Randomized Controlled Trial of Human Caloric Restriction: Feasibility and Effects on Predictors of Health Span and Longevity.” Journals of Gerontology, Series A, Biological Sciences and Medical Sciences 70, no. 9: 1097–1104. 10.1093/gerona/glv057. PMC4841173 26187233

[acel70785-bib-0222] Rocha, S. M. , M. B. Brás , B. J. Pereira , and C. J. Maia . 2026. “From Bench to Bedside: Translating the Histone Deacetylase Inhibitors Into Prostate Cancer Therapies.” Clinica Chimica Acta 587: 120949. 10.1016/j.cca.2026.120949. 41812786

[acel70785-bib-0223] Rodrigues, D. A. , P. S. M. Pinheiro , F. S. Sagrillo , M. L. Bolognesi , and C. A. M. Fraga . 2020. “Histone Deacetylases as Targets for the Treatment of Neurodegenerative Disorders: Challenges and Future Opportunities.” Medicinal Research Reviews 40, no. 6: 2177–2211. 10.1002/med.21701. 32588916

[acel70785-bib-0224] Rodrigues, D. A. , A. Roe , D. Griffith , and T. N. Chonghaile . 2022. “Advances in the Design and Development of PROTAC‐Mediated HDAC Degradation.” Current Topics in Medicinal Chemistry 22, no. 5: 408–424. 10.2174/1568026621666211015092047. 34649488

[acel70785-bib-0225] Roy, K. , M. Syed , P. Narad , and A. Sengupta . 2025. “Chapter Two—Genetic and Epigenetic Drivers of Neurodegenerative Disorders.” In Progress in Brain Research, edited by S. R. Ahmad , vol. 297, 53–130. Elsevier. 10.1016/bs.pbr.2025.08.002 41314755

[acel70785-bib-0226] Ryu, H. Y. , and M. Hochstrasser . 2021. “Histone Sumoylation and Chromatin Dynamics.” Nucleic Acids Research 49, no. 11: 6043–6052. 10.1093/nar/gkab280. PMC8216275 33885816

[acel70785-bib-0227] Sabari, B. R. , Z. Tang , H. Huang , et al. 2015. “Intracellular Crotonyl‐CoA Stimulates Transcription Through p300‐Catalyzed Histone Crotonylation.” Molecular Cell 58, no. 2: 203–215. 10.1016/j.molcel.2015.02.029. PMC4501262 25818647

[acel70785-bib-0228] Sadaie, M. , R. Salama , T. Carroll , et al. 2013. “Redistribution of the Lamin B1 Genomic Binding Profile Affects Rearrangement of Heterochromatic Domains and SAHF Formation During Senescence.” Genes & Development 27, no. 16: 1800–1808. 10.1101/gad.217281.113. PMC3759696 23964094

[acel70785-bib-0229] Saeki, K. , and K. Ozato . 2025. “Transcription Factors That Define the Epigenome Structures and Transcriptomes in Microglia.” Experimental Hematology 149: 104814. 10.1016/j.exphem.2025.104814. PMC12229243 40425139

[acel70785-bib-0230] Scheijen, E. E. , N. Veeningen , S. Duwé , et al. 2025. “Temporal and Spatial Pattern of DNA Damage in Neurons Following Spinal Cord Injury in Mice.” Journal of Biomedical Science 32, no. 1: 12. 10.1186/s12929-024-01104-8. PMC11756142 39844195

[acel70785-bib-0231] Seol, S.‐I. , S.‐A. Oh , D. Davaanyam , and J.‐K. Lee . 2025. “Blocking Peptidyl Arginine Deiminase 4 Confers Neuroprotective Effect in the Post‐Ischemic Brain Through Both NETosis‐Dependent and ‐Independent Mechanisms.” Acta Neuropathologica Communications 13, no. 1: 33. 10.1186/s40478-025-01951-y. PMC11834679 39966968

[acel70785-bib-0232] Shah, P. P. , G. Donahue , G. L. Otte , et al. 2013. “Lamin B1 Depletion in Senescent Cells Triggers Large‐Scale Changes in Gene Expression and the Chromatin Landscape.” Genes & Development 27, no. 16: 1787–1799. 10.1101/gad.223834.113. PMC3759695 23934658

[acel70785-bib-0233] Sheng, X. , H. Lin , P. A. Cole , and Y. Zhao . 2026. “Biochemistry and Regulation of Histone Lysine l‐Lactylation.” Nature Reviews Molecular Cell Biology 27, no. 2: 95–109. 10.1038/s41580-025-00876-7. PMC12920031 40830268

[acel70785-bib-0234] Shi, C. , F. Yuan , X. Chen , et al. 2026. “Histone H3K9 Lactylation Activates the TXNIP/NLRP3 Pathway to Drive Macrophage Inflammation After Spinal Cord Injury.” Redox Biology 92: 104078. 10.1016/j.redox.2026.104078. PMC13010529 41856077

[acel70785-bib-0235] Shimazu, T. , M. D. Hirschey , J. Newman , et al. 2013. “Suppression of Oxidative Stress by β‐Hydroxybutyrate, an Endogenous Histone Deacetylase Inhibitor.” Science 339, no. 6116: 211–214. 10.1126/science.1227166. PMC3735349 23223453

[acel70785-bib-0236] Si, W. Y. , C. L. Yang , S. L. Wei , et al. 2024. “Therapeutic Potential of Microglial SMEK1 in Regulating H3K9 Lactylation in Cerebral Ischemia‐Reperfusion.” Communications Biology 7, no. 1: 1701. 10.1038/s42003-024-07425-6. PMC11671539 39725685

[acel70785-bib-0237] Siano, G. , M. Varisco , M. Terrigno , et al. 2025. “Tau Mediates the Reshaping of the Transcriptional Landscape Toward Intermediate Alzheimer's Disease Stages.” Frontiers in Cell and Developmental Biology 12: 1459573. 10.3389/fcell.2024.1459573. PMC11739074 39830212

[acel70785-bib-0238] Singh, A. , A. Rani , and A. Mazumder . 2026. “Proteolysis Targeting Chimeras (PROTAC) for Targeting Alzheimer's Disease.” Discover Neuroscience 21, no. 1: 41. 10.1186/s13064-026-00276-3.

[acel70785-bib-0239] Singh, P. , and V. Paramanik . 2024. “DNA Methylation, Histone Acetylation in the Regulation of Memory and Its Modulation During Aging.” Frontiers in Aging Neuroscience 5: 1480932. 10.3389/fragi.2024.1480932. PMC11743476 39835300

[acel70785-bib-0240] Sprenger, K. G. , E. E. Lietzke , J. T. Melchior , and K. D. Bruce . 2025. “Lipid and Lipoprotein Metabolism in Microglia: Alzheimer's Disease Mechanisms and Interventions.” Journal of Lipid Research 66, no. 10: 100872. 10.1016/j.jlr.2025.100872. PMC12538436 40769380

[acel70785-bib-0241] Subasinghe, K. , C. Hall , M. Rowe , Z. Zhou , R. Barber , and N. Phillips . 2025. “Neuronal Enriched Extracellular Vesicle miR‐122‐5p as a Potential Biomarker for Alzheimer's Disease.” Cells 14, no. 22: 1784. 10.3390/cells14221784. PMC12651308 41294837

[acel70785-bib-0242] Sun, S. , Z. Xu , L. He , et al. 2024. “Metabolic Regulation of Cytoskeleton Functions by HDAC6‐Catalyzed α‐Tubulin Lactylation.” Nature Communications 15, no. 1: 8377. 10.1038/s41467-024-52729-0. PMC11437170 39333081

[acel70785-bib-0243] Sun, W. , H. Samimi , M. Gamez , H. Zare , and B. Frost . 2018. “Pathogenic Tau‐Induced piRNA Depletion Promotes Neuronal Death Through Transposable Element Dysregulation in Neurodegenerative Tauopathies.” Nature Neuroscience 21, no. 8: 1038–1048. 10.1038/s41593-018-0194-1. PMC6095477 30038280

[acel70785-bib-0244] Suter, T. , M. J. Friedman , C. Tazearslan , et al. 2026. “A Histone Code Functionally Linked to Replicative Senescence.” Aging Cell 25, no. 1: e70343. 10.1111/acel.70343. PMC12759110 41482664

[acel70785-bib-0245] Tang, Y. , T. Li , J. Li , et al. 2014. “Jmjd3 Is Essential for the Epigenetic Modulation of Microglia Phenotypes in the Immune Pathogenesis of Parkinson's Disease.” Cell Death & Differentiation 21, no. 3: 369–380. 10.1038/cdd.2013.159. PMC3921590 24212761

[acel70785-bib-0246] Tasdemir, N. , A. Banito , J.‐S. Roe , et al. 2016. “BRD4 Connects Enhancer Remodeling to Senescence Immune Surveillance.” Cancer Discovery 6, no. 6: 612–629. 10.1158/2159-8290.Cd-16-0217. PMC4893996 27099234

[acel70785-bib-0247] Tian, L. , G. Long , S. Zhu , et al. 2025. “Nanoimmunomodulation of the Aβ‐STING Feedback Machinery in Microglia for Alzheimer's Disease Treatment.” Proceedings of the National academy of Sciences of the United States of America 122, no. 22: e2427257122. 10.1073/pnas.2427257122. PMC12146763 40434641

[acel70785-bib-0248] Tie, F. , R. Banerjee , C. A. Stratton , et al. 2009. “CBP‐Mediated Acetylation of Histone H3 Lysine 27 Antagonizes *Drosophila* Polycomb Silencing.” Development 136, no. 18: 3131–3141. 10.1242/dev.037127. PMC2730368 19700617

[acel70785-bib-0249] Traetta, M. E. , H. A. Vecchiarelli , and M. Tremblay . 2025. “Fundamental Neurochemistry Review: Lipids Across Microglial States.” Journal of Neurochemistry 169, no. 1: e16259. 10.1111/jnc.16259. PMC11655966 39696753

[acel70785-bib-0250] Turner, R. S. , R. G. Thomas , S. Craft , et al. 2015. “A Randomized, Double‐Blind, Placebo‐Controlled Trial of Resveratrol for Alzheimer Disease.” Neurology 85, no. 16: 1383–1391. 10.1212/WNL.0000000000002035. PMC4626244 26362286

[acel70785-bib-0251] Valiukas, Z. , K. Tangalakis , V. Apostolopoulos , and J. Feehan . 2025. “Microglial Activation States and Their Implications for Alzheimer's Disease.” Journal of Prevention of Alzheimer's Disease 12, no. 1: 100013. 10.1016/j.tjpad.2024.100013. PMC12184064 39800461

[acel70785-bib-0252] Villarreal, A. , C. Vidos , M. Monteverde Busso , M. B. Cieri , and A. J. Ramos . 2021. “Pathological Neuroinflammatory Conversion of Reactive Astrocytes Is Induced by Microglia and Involves Chromatin Remodeling.” Frontiers in Pharmacology 12: 689346. 10.3389/fphar.2021.689346. PMC8255379 34234677

[acel70785-bib-0253] Vinten, K. T. , M. M. Tretowicz , E. Coskun , et al. 2025. “NAD(+) Precursor Supplementation in Human Ageing: Clinical Evidence and Challenges.” Nature Metabolism 7, no. 10: 1974–1990. 10.1038/s42255-025-01387-7. 41083806

[acel70785-bib-0254] Vizioli, M. G. , T. Liu , K. N. Miller , et al. 2020. “Mitochondria‐to‐Nucleus Retrograde Signaling Drives Formation of Cytoplasmic Chromatin and Inflammation in Senescence.” Genes & Development 34, no. 5–6: 428–445. 10.1101/gad.331272.119. PMC7050483 32001510

[acel70785-bib-0255] Voukia, F. , L. Moesgaard , J. Kongsted , and J. Mecinović . 2026. “Inhibition of Human Histone Lysine Methyltransferases by a Redox‐Labile S‐Adenosyl‐L‐Homocysteine Analog.” Scientific Reports 16: 19173. 10.1038/s41598-026-52984-9. PMC13282376 42129275

[acel70785-bib-0256] Vreones, M. , M. Mustapic , R. Moaddel , et al. 2023. “Oral Nicotinamide Riboside Raises NAD+ and Lowers Biomarkers of Neurodegenerative Pathology in Plasma Extracellular Vesicles Enriched for Neuronal Origin.” Aging Cell 22, no. 1: e13754. 10.1111/acel.13754. PMC9835564 36515353

[acel70785-bib-0257] Wang, C. , D. Shen , Y. Hu , et al. 2023. “Selective Targeting of Class I HDAC Reduces Microglial Inflammation in the Entorhinal Cortex of Young APP/PS1 Mice.” International Journal of Molecular Sciences 24, no. 5: 4805. 10.3390/ijms24054805. PMC10003411 36902234

[acel70785-bib-0258] Wang, C. , H. Yue , Z. Hu , et al. 2020. “Microglia Mediate Forgetting via Complement‐Dependent Synaptic Elimination.” Science 367, no. 6478: 688–694. 10.1126/science.aaz2288. 32029629

[acel70785-bib-0259] Wang, H. , Y. Qiao , H. Lu , L. Xiao , and Q. Cheng . 2025. “Astrocyte Autophagy‐Neuroinflammation Axis in Ischemic Stroke: From Molecular Mechanisms to Translational Medicine.” Neuroscience 589: 128–139. 10.1016/j.neuroscience.2025.10.036. 41138968

[acel70785-bib-0260] Wang, H. , H. Xia , J. Bai , et al. 2025. “H4K12 Lactylation‐Regulated NLRP3 Is Involved in Cigarette Smoke‐Accelerated Alzheimer‐Like Pathology Through mTOR‐Regulated Autophagy and Activation of Microglia.” Journal of Hazardous Materials 488: 137310. 10.1016/j.jhazmat.2025.137310. 39862777

[acel70785-bib-0261] Wang, J. , P. Yang , T. Yu , et al. 2022. “Lactylation of PKM2 Suppresses Inflammatory Metabolic Adaptation in Pro‐Inflammatory Macrophages.” International Journal of Biological Sciences 18, no. 16: 6210–6225. 10.7150/ijbs.75434. PMC9682528 36439872

[acel70785-bib-0262] Wang, M. , J. Liang , X. Zhu , et al. 2026. “Natural Plant Bioactives as Regulators of Histone Modifications: Bridging Epigenetics and Anticancer Therapy.” Phytotherapy Research 40, no. 8: 5208–5254. 10.1002/ptr.70386. PMC13436294 42186257

[acel70785-bib-0263] Wang, R. , F. Sun , N. Wang , et al. 2025. “Potential Therapeutic Effects of Astragaloside IV in Parkinson's Disease Models via Modulation of α‐Syn‐γH2AX‐STING‐IFN‐I Axis.” Molecular Neurobiology 63, no. 1: 11. 10.1007/s12035-025-05377-4. 41204052

[acel70785-bib-0264] Wang, S. , J. Wang , Z. Hu , L. Wu , and L. Huang . 2026. “Role of Glycolysis‐Mediated Histone Lactylation in Microglial Activation and Progression of Neurodegenerative Diseases.” Experimental Neurology 399: 115663. 10.1016/j.expneurol.2026.115663. 41577113

[acel70785-bib-0265] Wang, X. , H. J. He , X. Xiong , et al. 2021. “NAD(+) in Alzheimer's Disease: Molecular Mechanisms and Systematic Therapeutic Evidence Obtained in Vivo.” Frontiers in Cell and Developmental Biology 9: 668491. 10.3389/fcell.2021.668491. PMC8369418 34414179

[acel70785-bib-0266] Wang, X. , C. Liu , Y. Chen , et al. 2026. “Lactate and Lactylation: Mechanisms, Function, Diseases, and Therapeutic Targets.” Molecular Biomedicine 7, no. 1: 71. 10.1186/s43556-026-00472-x. PMC13190961 42159990

[acel70785-bib-0267] Wang, X. , Q. Liu , H.‐t. Yu , et al. 2024. “A Positive Feedback Inhibition of Isocitrate Dehydrogenase 3β on Paired‐Box Gene 6 Promotes Alzheimer‐Like Pathology.” Signal Transduction and Targeted Therapy 9, no. 1: 105. 10.1038/s41392-024-01812-5. PMC11056379 38679634

[acel70785-bib-0268] Wang, X. , G. Zhou , J. Xiong , et al. 2025. “H4K12 Lactylation Activated‐Spp1 in Reprogrammed Microglia Improves Functional Recovery After Spinal Cord Injury.” CNS Neuroscience & Therapeutics 31, no. 2: e70232. 10.1111/cns.70232. PMC11821456 39939834

[acel70785-bib-0269] Wang, X. , Q. Zhu , J. Liu , et al. 2026. “Integrated Epigenetic Networks in Aging: From Histone to RNA Modifications.” Journal of Translational Medicine 24, no. 1: 566. 10.1186/s12967-026-07990-w. PMC13101344 41832537

[acel70785-bib-0270] Wang, Y. , B. Feng , R. Hong , D. Qiu , J. Zhao , and L. Zhao . 2026. “Glial Lactate Metabolism and Transport in Alzheimer's Disease.” Frontiers in Aging Neuroscience 18: 1731089. 10.3389/fnagi.2026.1731089. PMC13038885 41930368

[acel70785-bib-0271] Wang, Y. , Y. R. Guo , K. Liu , et al. 2017. “KAT2A Coupled With the α‐KGDH Complex Acts as a Histone H3 Succinyltransferase.” Nature 552, no. 7684: 273–277. 10.1038/nature25003. PMC5841452 29211711

[acel70785-bib-0272] Wang, Y. , H. Zhang , L. Hua , et al. 2022. “Curcumin Prevents Alzheimer's Disease Progression by Upregulating JMJD3.” American Journal of Translational Research 14, no. 8: 5280–5294. PMC9452350 36105064

[acel70785-bib-0273] Wei, L. , X. Yang , J. Wang , et al. 2023. “H3K18 Lactylation of Senescent Microglia Potentiates Brain Aging and Alzheimer's Disease Through the NFκB Signaling Pathway.” Journal of Neuroinflammation 20, no. 1: 208. 10.1186/s12974-023-02879-7. PMC10494370 37697347

[acel70785-bib-0274] Wendeln, A. C. , K. Degenhardt , L. Kaurani , et al. 2018. “Innate Immune Memory in the Brain Shapes Neurological Disease Hallmarks.” Nature 556, no. 7701: 332–338. 10.1038/s41586-018-0023-4. PMC6038912 29643512

[acel70785-bib-0275] Weng, Y. , T. He , M. Li , et al. 2026. “Microglial Histone H3K18 Crotonylation Promotes STAT1 Expression and Induces Cognitive Deficit in Alzheimer Disease.” Frontiers in Immunology 17: 1744375. 10.3389/fimmu.2026.1744375. PMC12886003 41676156

[acel70785-bib-0276] Weyh, M. , M.‐L. Jokisch , T.‐A. Nguyen , M. Fottner , and K. Lang . 2024. “Deciphering Functional Roles of Protein Succinylation and Glutarylation Using Genetic Code Expansion.” Nature Chemistry 16, no. 6: 913–921. 10.1038/s41557-024-01500-5. PMC11164685 38531969

[acel70785-bib-0277] White, M. , R. H. Kofoed , C. L. Dibia , et al. 2025. “Focused Ultrasound Enhances Targeted Gene Delivery of Intravenous AAV to the Brain.” Alzheimer's & Dementia 21, no. S5: e103550. 10.1002/alz70859_103550.

[acel70785-bib-0278] Williams, J. B. , Q. Cao , W. Wang , et al. 2023. “Inhibition of Histone Methyltransferase Smyd3 Rescues NMDAR and Cognitive Deficits in a Tauopathy Mouse Model.” Nature Communications 14, no. 1: 91. 10.1038/s41467-022-35749-6. PMC9822922 36609445

[acel70785-bib-0279] Wu, C. , J. Li , L. Lu , M. Li , Y. Yuan , and J. Li . 2024. “OGT and OGA: Sweet Guardians of the Genome.” Journal of Biological Chemistry 300, no. 4: 107141. 10.1016/j.jbc.2024.107141. PMC10981121 38447797

[acel70785-bib-0280] Wu, C.‐Y. , A. C. Kupferschmid , L. Chen , et al. 2025. “Cognitive and Alzheimer's Disease Biomarker Effects of Oral Nicotinamide Riboside (NR) Supplementation in Older Adults With Subjective Cognitive Decline and Mild Cognitive Impairment.” Alzheimer's & Dementia: Translational Research & Clinical Interventions 11, no. 1: e70023. 10.1002/trc2.70023. PMC11733434 39817194

[acel70785-bib-0281] Wu, D. , J. K. Sun , and K. H. Chow . 2024. “Neuronal Cell Cycle Reentry Events in the Aging Brain Are More Prevalent in Neurodegeneration and Lead to Cellular Senescence.” PLoS Biology 22, no. 4: e3002559. 10.1371/journal.pbio.3002559. PMC11037540 38652714

[acel70785-bib-0282] Wu, T. , D. Su , L. Zhang , et al. 2024. “Mitochondrial Control of Proteasomal Psmb5 Drives the Differentiation of Tissue‐Resident Memory T Cells in Patients With Rheumatoid Arthritis.” Arthritis and Rheumatology 76, no. 12: 1743–1757. 10.1002/art.42954. 39037181

[acel70785-bib-0283] Wu, X. , T. He , F. He , and L. Liu . 2025. “Is Postoperative Cognitive Dysfunction a Disease of Microglial Inflammatory Memory? A State‐Transition Model From Metabolic Stress to Epigenetic Lock‐In.” Frontiers in Molecular Neuroscience 18: 1648161. 10.3389/fnmol.2025.1648161. PMC12364888 40843245

[acel70785-bib-0284] Wu, Z. , J. Qu , and G.‐H. Liu . 2024. “Roles of Chromatin and Genome Instability in Cellular Senescence and Their Relevance to Ageing and Related Diseases.” Nature Reviews Molecular Cell Biology 25, no. 12: 979–1000. 10.1038/s41580-024-00775-3. 39363000

[acel70785-bib-0285] Xie, J.‐y. , J. Ju , P. Zhou , et al. 2024. “The Mechanisms, Regulations, and Functions of Histone Lysine Crotonylation.” Cell Death Discovery 10, no. 1: 66. 10.1038/s41420-024-01830-w. PMC10853258 38331935

[acel70785-bib-0286] Xie, L. , R. N. Sheehy , A. Muneer , et al. 2025. “Development of a Brain‐Penetrant G9a Methylase Inhibitor to Target Alzheimer's Disease‐Associated Proteopathology.” Nature Communications 16, no. 1: 4222. 10.1038/s41467-025-59128-z. PMC12056044 40328756

[acel70785-bib-0287] Xie, Z. , D. Zhang , D. Chung , et al. 2016. “Metabolic Regulation of Gene Expression by Histone Lysine β‐Hydroxybutyrylation.” Molecular Cell 62, no. 2: 194–206. 10.1016/j.molcel.2016.03.036. PMC5540445 27105115

[acel70785-bib-0288] Xu, C. , L. Wang , P. Fozouni , et al. 2020. “SIRT1 Is Downregulated by Autophagy in Senescence and Ageing.” Nature Cell Biology 22, no. 10: 1170–1179. 10.1038/s41556-020-00579-5. PMC7805578 32989246

[acel70785-bib-0289] Xu, D. C. , H. Sas‐Nowosielska , G. Donahue , et al. 2024. “Histone Acetylation in an Alzheimer's Disease Cell Model Promotes Homeostatic Amyloid‐Reducing Pathways.” Acta Neuropathologica Communications 12, no. 1: 3. 10.1186/s40478-023-01696-6. PMC10759377 38167174

[acel70785-bib-0290] Yamagishi, M. , Y. Kuze , S. Kobayashi , et al. 2024. “Mechanisms of Action and Resistance in Histone Methylation‐Targeted Therapy.” Nature 627, no. 8002: 221–228. 10.1038/s41586-024-07103-x. PMC10917674 38383791

[acel70785-bib-0291] Yan, X. , C. Georgopoulou , H.‐M. Lee , et al. 2026. “Microglia Activation by Derepression of Endogenous Retroviruses Drives Inflammation and Cellular Senescence.” Nature Neuroscience 29: 2480–2498. 10.1038/s41593-026-02404-y. PMC13633122 42608571

[acel70785-bib-0292] Yang, G. , Y. Ren , P. Zhong , et al. 2026. “Histone Demethylase PHF2 Regulates Inflammatory Genes in Alzheimer's Disease.” Molecular Psychiatry 31, no. 2: 845–859. 10.1038/s41380-025-03181-z. PMC12746590 40849543

[acel70785-bib-0293] Yang, M. , X. Tang , M. Iqfath , et al. 2025. “Multimodal Nano‐DESI Mass Spectrometry Imaging Reveals Phospholipids Accumulation in and Around Amyloid Plaques in Alzheimer's Disease.” ACS Chemical Neuroscience 16, no. 16: 3127–3137. 10.1021/acschemneuro.5c00144. 40741812

[acel70785-bib-0294] Yang, S. J. , A. A. Lin , H. Shen , et al. 2026. “Microfluidic Nanomagnetically Isolated Neuron‐ and Astrocyte‐Derived Extracellular Vesicles to Differentiate Lewy Body and Alzheimer's Disease.” NPJ Biosensors 3, no. 1: 19. 10.1038/s44328-026-00086-x. PMC12982126 41836608

[acel70785-bib-0295] Yao, W. , X. Hu , and X. Wang . 2024. “Crossing Epigenetic Frontiers: The Intersection of Novel Histone Modifications and Diseases.” Signal Transduction and Targeted Therapy 9, no. 1: 232. 10.1038/s41392-024-01918-w. PMC11403012 39278916

[acel70785-bib-0296] Yassine, H. N. , C. Hugo , B. O'Donovan , et al. 2025. “APOE‐Targeted Therapeutics for Alzheimer's Disease.” Journal of Neuroscience 45, no. 46: e1388252025. 10.1523/JNEUROSCI.1388-25.2025. PMC12614056 41224653

[acel70785-bib-0297] Ye, J. , S. Zhong , H. Wan , et al. 2025. “Upregulated Astrocyte HDAC7 Induces Alzheimer‐Like Tau Pathologies via Deacetylating Transcription Factor‐EB and Inhibiting Lysosome Biogenesis.” Molecular Neurodegeneration 20, no. 1: 5. 10.1186/s13024-025-00796-2. PMC11727263 39806423

[acel70785-bib-0298] Yu, H. , F. Wang , J. Q. Yuan , et al. 2026. “HMGCS2‐Dependent Beta‐OHB/H3K9bhb Ameliorates Synaptic Plasticity and Cognition in Alzheimer's Disease.” Experimental and Molecular Medicine 58, no. 3: 813–831. 10.1038/s12276-026-01664-9. PMC13049083 41792234

[acel70785-bib-0299] Yücel, A. D. , and V. N. Gladyshev . 2026. “Systemic Epigenetic Dysregulation as a Driver of Ageing and a Therapeutic Target.” Nature Reviews Molecular Cell Biology 27, no. 7: 528–542. 10.1038/s41580-026-00958-0. 41896334

[acel70785-bib-0300] Zaman, W. , A. Ayaz , and A. Ullah . 2026. “Decoding Lactate Signals in Alzheimer's Disease: Redox Control, Receptor Pharmacology, Lactylation Chemistry, and Neuroglial Vulnerability.” Biochemical Pharmacology 253, no. Pt 1: 118275. 10.1016/j.bcp.2026.118275. 42476266

[acel70785-bib-0301] Zeng, Q. , W. Wang , W. Tian , et al. 2026. “Cell‐Type‐Specific Transposon Demethylation and TAD Remodeling in Aging Mouse Brain.” Cell 189, no. 7: 2148–2166.e2127. 10.1016/j.cell.2026.02.015. PMC13007725 41819104

[acel70785-bib-0302] Zhai, G. , Z. Niu , Z. Jiang , et al. 2024. “DPF2 Reads Histone Lactylation to Drive Transcription and Tumorigenesis.” Proceedings of the National academy of Sciences of the United States of America 121, no. 50: e2421496121. 10.1073/pnas.2421496121. PMC11648877 39636855

[acel70785-bib-0303] Zhang, C. , L. Zhou , M. Zhang , et al. 2024. “H3K18 Lactylation Potentiates Immune Escape of Non–Small Cell Lung Cancer.” Cancer Research 84, no. 21: 3589–3601. 10.1158/0008-5472.Can-23-3513. 39137401

[acel70785-bib-0304] Zhang, D. , Z. Tang , H. Huang , et al. 2019. “Metabolic Regulation of Gene Expression by Histone Lactylation.” Nature 574, no. 7779: 575–580. 10.1038/s41586-019-1678-1. PMC6818755 31645732

[acel70785-bib-0305] Zhang, F. , J. Zhou , P. Lu , et al. 2024. “Lactylation of Histone by BRD4 Regulates Astrocyte Polarization After Experimental Subarachnoid Hemorrhage.” Journal of Neuroinflammation 21, no. 1: 186. 10.1186/s12974-024-03185-6. PMC11290164 39080649

[acel70785-bib-0306] Zhang, L. , C. Liu , J. Wu , et al. 2014. “Tubastatin A/ACY‐1215 Improves Cognition in Alzheimer's Disease Transgenic Mice.” Journal of Alzheimer's Disease 41, no. 4: 1193–1205. 10.3233/JAD-140066. 24844691

[acel70785-bib-0307] Zhang, M. , W. Wang , Q. Ye , et al. 2024. “Histone Deacetylase Inhibitors VPA and WT161 Ameliorate the Pathological Features and Cognitive Impairments of the APP/PS1 Alzheimer's Disease Mouse Model by Regulating the Expression of APP Secretases.” Alzheimer's Research & Therapy 16, no. 1: 15. 10.1186/s13195-024-01384-0. PMC10799458 38245771

[acel70785-bib-0308] Zhang, N. , R. Zhao , X. Zhong , et al. 2026. “Time‐Resolved Multiomics Profiling Reveals Chromatin O‐GlcNAc Modification Promotes Senescence‐Associated Transcriptional Program.” Nature Communications 17, no. 1: 1406. 10.1038/s41467-025-68143-z. PMC12881531 41507163

[acel70785-bib-0309] Zhang, R. , J. Bons , G. Scheidemantle , et al. 2023. “Histone Malonylation Is Regulated by SIRT5 and KAT2A.” iScience 26, no. 3: 106193. 10.1016/j.isci.2023.106193. PMC9985052 36879797

[acel70785-bib-0310] Zhang, R. , M. V. Poustovoitov , X. Ye , et al. 2005. “Formation of MacroH2A‐Containing Senescence‐Associated Heterochromatin Foci and Senescence Driven by ASF1a and HIRA.” Developmental Cell 8, no. 1: 19–30. 10.1016/j.devcel.2004.10.019. 15621527

[acel70785-bib-0311] Zhang, W.‐X. , Y.‐L. Yang , J.‐X. Yu , L.‐H. Jia , and H.‐C. Huang . 2025. “APOE4‐Driven Lipid Metabolic Dysregulation in Alzheimer's Disease: Multi‐Pathway Mechanisms and Therapeutic Perspectives.” Biochemical and Biophysical Research Communications 778: 152335. 10.1016/j.bbrc.2025.152335. 40669336

[acel70785-bib-0312] Zhang, X. , Y. Wang , J. Yuan , et al. 2018. “Macrophage/Microglial Ezh2 Facilitates Autoimmune Inflammation Through Inhibition of Socs3.” Journal of Experimental Medicine 215, no. 5: 1365–1382. 10.1084/jem.20171417. PMC5940261 29626115

[acel70785-bib-0313] Zhang, X. , and Z. Yin . 2026. “Epigenetic Activation of PDLIM7 via H3K27 Acetylation Mitigates Neuroinflammation and Neurodegeneration in Parkinson's Disease Models.” Journal of Bioenergetics and Biomembranes 58: 21. 10.1007/s10863-026-10088-z. 41874918

[acel70785-bib-0314] Zhang, Y.‐M. , F. Yang , Q. Li , and J.‐N. Zhang . 2025. “Role of Histone Lactylation in Neurological Disorders.” International Journal of Molecular Sciences 26, no. 16: 7949. 10.3390/ijms26167949 PMC12386771 40869269

[acel70785-bib-0315] Zhang, Z. , M. Tan , Z. Xie , L. Dai , Y. Chen , and Y. Zhao . 2011. “Identification of Lysine Succinylation as a New Post‐Translational Modification.” Nature Chemical Biology 7, no. 1: 58–63. 10.1038/nchembio.495. PMC3065206 21151122

[acel70785-bib-0316] Zhang, Z. , R. Wang , H. Zhou , et al. 2024. “Inhibition of EHMT1/2 Rescues Synaptic Damage and Motor Impairment in a PD Mouse Model.” Cellular and Molecular Life Sciences 81, no. 1: 128. 10.1007/s00018-024-05176-5. PMC10933175 38472451

[acel70785-bib-0317] Zhao, Y. , Z. Wang , Y. Mao , et al. 2020. “NEAT1 Regulates Microtubule Stabilization via FZD3/GSK3β/P‐Tau Pathway in SH‐SY5Y Cells and APP/PS1 Mice.” Aging 12, no. 22: 23233–23250. 10.18632/aging.104098. PMC7746375 33221742

[acel70785-bib-0318] Zheng, Y. , A. Liu , Z. J. Wang , et al. 2019. “Inhibition of EHMT1/2 Rescues Synaptic and Cognitive Functions for Alzheimer's Disease.” Brain 142, no. 3: 787–807. 10.1093/brain/awy354. PMC6391616 30668640

[acel70785-bib-0319] Zhong, Y. , L. Wei , and R. Xue . 2026. “GSK126 Mitigates Oxidative Stress in Alzheimer Disease Models via an Enhancer of Zeste Homolog 2‐H3 Lysine 27 Trimethylation‐Superoxide Dismutase 1 Axis.” Journal of Neuropathology and Experimental Neurology 85, no. 5: 464–474. 10.1093/jnen/nlag008. 41700920

[acel70785-bib-0320] Zhou, J. , Y. Yang , S. Liu , et al. 2026. “Microglial Serine Racemase Knockout Alleviates Alzheimer‐Like Neuropathology and Behavioral Deficit via Lactylation‐Mediated Anti‐Inflammation.” Communications Biology 9, no. 1: 493. 10.1038/s42003-026-09772-y. PMC13056901 41741759

[acel70785-bib-0321] Zhou, M.‐M. , and P. A. Cole . 2025. “Targeting Lysine Acetylation Readers and Writers.” Nature Reviews Drug Discovery 24, no. 2: 112–133. 10.1038/s41573-024-01080-6. PMC11798720 39572658

[acel70785-bib-0322] Zhou, T. , X. Cheng , Y. He , et al. 2022. “Function and Mechanism of Histone Beta‐Hydroxybutyrylation in Health and Disease.” Frontiers in Immunology 13: 981285. 10.3389/fimmu.2022.981285. PMC9511043 36172354

[acel70785-bib-0323] Zhu, R. , X. Ye , X. Lu , et al. 2025. “ACSS2 Acts as a Lactyl‐CoA Synthetase and Couples KAT2A to Function as a Lactyltransferase for Histone Lactylation and Tumor Immune Evasion.” Cell Metabolism 37, no. 2: 361–376.e367. 10.1016/j.cmet.2024.10.015. 39561764

[acel70785-bib-0324] Zong, B. , F. Yu , F. Li , P. Sun , and L. Li . 2025. “Beyond Fuel: Exercise‐Induced Lactate as a Metabolic‐Epigenetic Regulator in Central Nervous System Health and Disease.” Biomolecules 16, no. 1: 43. 10.3390/biom16010043. PMC12838923 41594585

[acel70785-bib-0325] Zorro Shahidian, L. , M. Haas , S. Le Gras , et al. 2021. “Succinylation of H3K122 Destabilizes Nucleosomes and Enhances Transcription.” EMBO Reports 22, no. 3: EMBR202051009. 10.15252/embr.202051009. PMC7926236 33512761

